# Causal associations of sleep traits with cancer incidence and mortality

**DOI:** 10.3389/fgene.2023.1309069

**Published:** 2023-11-23

**Authors:** Shanshan Tian, Longtao Huangfu, Yanping Bao, Sizhi Ai, Suhua Chang, Qianwen Wang, Ximei Zhu, Wei Yan, Jie Shi, Le Shi, Jiahui Deng, Lin Lu

**Affiliations:** ^1^ Peking University Sixth Hospital, Peking University Institute of Mental Health, NHC Key Laboratory of Mental Health (Peking University), National Clinical Research Center for Mental Disorders (Peking University Sixth Hospital), Beijing, China; ^2^ Key Laboratory of Carcinogenesis and Translational Research (Ministry of Education), Division of Gastrointestinal Cancer Translational Research Laboratory, Peking University Cancer Hospital & Institute, Beijing, China; ^3^ National Institute on Drug Dependence and Beijing Key Laboratory of Drug Dependence, Peking University, Beijing, China; ^4^ Department of Cardiology, Heart Center, The First Affiliated Hospital of Xinxiang Medical University, Xinxiang, Henan, China; ^5^ Center for Sleep and Circadian Medicine, The Affiliated Brain Hospital of Guangzhou Medical University, Xinxiang, Guangdong, China; ^6^ Peking-Tsinghua Center for Life Sciences, PKU-IDG/McGovern Institute for Brain Research, Beijing, China

**Keywords:** sleep traits, pan-cancer incidence, mortality, mendelian randomization, causal relationships

## Abstract

To explore the correlation and causality between multidimensional sleep traits and pan-cancer incidence and mortality among patients with cancer. The multivariable Cox regression, linear and nonlinear Mendelian randomization (MR), and survival curve analyses were conducted to assess the impacts of chronotype, sleep duration, and insomnia symptoms on pan-cancer risk (N = 326,417 from United Kingdom Biobank) and mortality (N = 23,956 from United Kingdom Biobank). In the Cox regression, we observed a linear and J-shaped association of sleep duration with pan-cancer incidence and mortality among cancer patients respectively. In addition, there was a positive association of insomnia with pan-cancer incidence (HR, 1.03, 95% CI: 1.00–1.06, *p* = 0.035), all-cause mortality (HR, 1.17, 95% CI: 1.06–1.30, *p* = 0.002) and cancer mortality among cancer patients (HR, 1.25, 95% CI: 1.11–1.41, *p* < 0.001). In the linear MR, there was supporting evidence of positive associations between long sleep duration and pan-cancer incidence (OR, 1.41, 95% CI: 1.08–1.84, *p* = 0.012), and there was a positive association between long sleep duration and all-cause mortality in cancer patients (OR, 5.56, 95% CI: 3.15–9.82, *p* = 3.42E-09). Meanwhile, a strong association between insomnia and all-cause mortality in cancer patients (OR, 1.41, 95% CI: 1.27–1.56, *p* = 4.96E-11) was observed in the linear MR. These results suggest that long sleep duration and insomnia play important roles in pan-cancer risk and mortality among cancer patients. In addition to short sleep duration and insomnia, our findings highlight the effect of long sleep duration in cancer prevention and prognosis.

## Introduction

In 2019, the World Health Organization (WHO) reported that cancer had become the first or second leading cause of death in more than 100 countries globally (Sung et al., 2021). Unhealthy lifestyle behaviors are associated with an increased risk of cancer. Emerging evidence also suggests that poor sleep is associated with the risk of cancer (Xiao et al., 2017; Wang J. et al., 2021), and poor sleep quality is persistent in cancer patients and survivors (Davidson et al., 2002; Irwin et al., 2013; Garland et al., 2014; Yoon et al., 2015). Several studies have shown that short and long sleep durations are associated with a higher risk of several cancers (Richmond et al., 2019; Wang J. et al., 2021; Tao et al., 2021; Xie et al., 2021; Lv et al., 2022; Peeri et al., 2022). Although several studies have explored the relationship between sleep duration and cancer mortality, inconsistent results have been found in observational studies (Chien et al., 2010; Phipps et al., 2016; Marinac et al., 2017; Trudel-Fitzgerald et al., 2017; Wong et al., 2017; Khan et al., 2018; Svensson et al., 2021; Tao et al., 2021). Some studies showed a U-shaped relationship (Wong et al., 2017), while others only found a relationship for long sleep duration (Marinac et al., 2017; Trudel-Fitzgerald et al., 2017) or no relationship (Phipps et al., 2016). In addition, no study has explored sleep traits and 5-year cancer mortality in cancer survivors, which is an important survival indicator among cancer patients. Because of self-reporting and residual confounding effects in observational studies, causal relationships are still unclear.

Mendelian randomization (MR) analysis provides a method to help clarify causal association by utilizing genetic variants of sleep traits as instrumental variables to improve inference in observational studies (Smith and Ebrahim, 2003; Davey Smith and Hemani, 2014; Davies et al., 2018; Sanderson, 2021). MR could overcome some limitations of traditional observational studies, because genetic variants are typically not associated with confounders. Thus differences between those who do not carry the variant and those who do can be attributed to the difference in the risk factor (Emdin et al., 2017). To date, several genome-wide association studies (GWAS) have found genetic variants that are robustly associated with sleep traits, including chronotype, sleep duration and insomnia symptoms (Dashti et al., 2019; Jansen et al., 2019; Jones et al., 2019). Recent studies have explored the causal associations between sleep duration and several cancers (Richmond et al., 2019; Wang J. et al., 2021; Hayes et al., 2022). However, sleep health is a multidimensional issue; several sleep traits are associated with disease risk and should be considered together. It is not convincing to consider only the causal association between one factor of sleep traits (e.g., chronotype, sleep duration, or insomnia symptoms) and the risk and mortality of cancer. In addition, we could accurately clarify the effects of sleep traits on cancer mortality only if we focused on cancer patients. Thus, there is a need to explore the potential causal associations between sleep traits and pan-cancer incidence and mortality among patients with cancer.

In the present study, we aimed to systematically explore the correlation and causality between multidimensional sleep traits and pan-cancer incidence and mortality among patients with cancer. We first used multivariable Cox proportional hazard models to estimate the associations between sleep traits and pan-cancer incidence. Furthermore, we investigated whether there was a causal relationship using linear and nonlinear MR analysis. We also analyzed the associations and causal relationship between sleep straits and mortality among cancer patients, including all-cause mortality of cancer patients, 5-year cancer mortality and cancer mortality, using multivariable Cox proportional hazard models and MR analyses. Finally, Kaplan‒Meier estimation was used to compare survival analyses across different groups of cancer patients.

## Materials and methods

### Study participants

**FIGURE 1:**
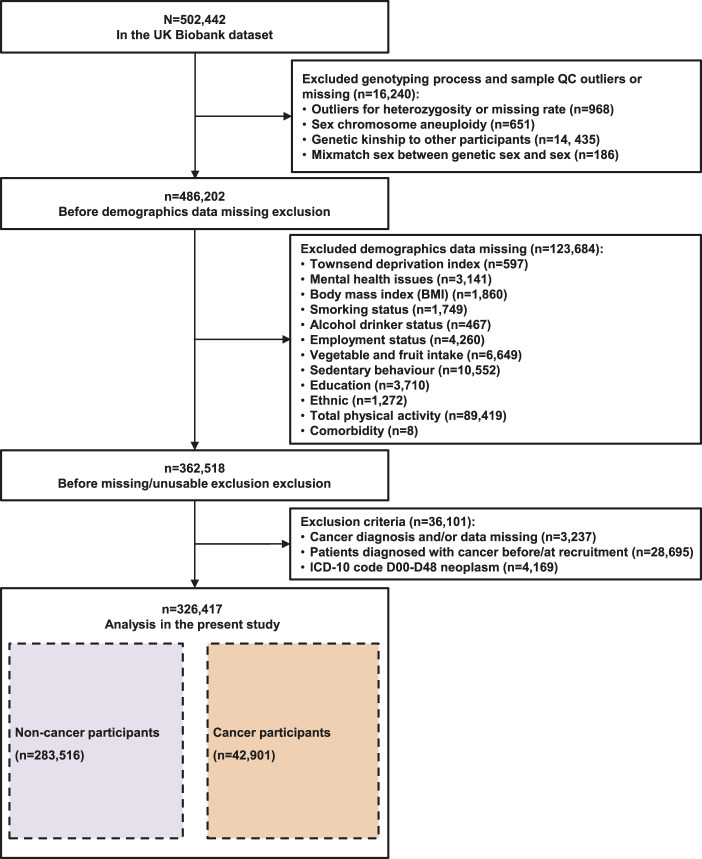
Participants selection flowchart.

The United Kingdom Biobank is a prospective cohort of more than 500,000 adults aged 37–63 years, recruited from 22 centers across the United Kingdom between 2006 and 2010 (Fry et al., 2017). We extracted all information needed for this study from the United Kingdom Biobank (Fry et al., 2017). Considering the confounding factor of ancestry, we analyzed only unrelated participants of European ancestry. Overall, 326,417 participants were included in pan-cancer incidence analysis. The detailed study protocol is shown in Figure 1. Among those participants, the average follow-up duration was 11.28 ± 2.64 years, in which 283,516 participants did not develop cancer and 42,901 participants developed cancer. In our study, 23,956 cancer patients were included in the mortality analysis. The detailed study protocol is shown in Supplementary Table S8. Among those with cancer, the average follow-up duration was 11.63 ± 2.70 years, in which 3,687 cancer patients were died (2,864 with cancer deaths and 823 with noncancer deaths). Descriptions and sources of the selected covariates, genotyping process and sample quality control in the United Kingdom Biobank are shown in Supplementary Table S1.

### Ascertainment of exposure and outcomes

The chronotype, sleep duration, and insomnia symptoms were assessed. Chronotype was assessed using the standardized question “Do you consider yourself to be?” with one of the six possible answers: “Definitely a ‘morning’ person,” “More a ‘morning’ than ‘evening’ person,” “More an ‘evening’ than ‘morning’ person,” “Definitely an ‘evening’ person,” “Do not know,” or “Prefer not to answer.” In the present study, we classified “Definitely a ‘morning’ person,” “More a ‘morning’ than ‘evening’ person,” “More an ‘evening’ than ‘morning’ person,” “Definitely an ‘evening’ person,” as definite morning, more morning, more evening and definite evening, respectively, and “Do not know” and “Prefer not to answer” responses were considered as missing data. Sleep duration was assessed by asking: “About how many hours of sleep do you get every 24 h (Please include naps).” The answer can contain only integer values. “Do not know” and “Prefer not to answer” responses were considered missing data. Binary variables for ≤5h, 6h, 7h, 8h, and ≥9 h were also derived. Insomnia symptoms were assessed using a single item: “Do you have trouble falling asleep at night or do you wake up in the middle of the night?” with answers: “Never/rarely,” “Sometimes,” “Usually,” or “Prefer not to answer.” Participants who chose “Prefer not to answer” were also considered missing data. Binary variables for insomnia symptoms were generated as “Never/rarely”, “Sometimes”, and “Usually”.

In this study, pan-cancer was defined as malignant neoplasms according to the International Classification of Diseases edition 10 (ICD-10), excluding *in situ* (ICD code: D00-D09), benign (ICD code: D10-D36), uncertain or non-well-defined cancers (ICD code: D37-D48), which is the same approach as that used by other studies (Huang et al., 2022; Zhou et al., 2022; Zhu et al., 2022). Thus, our study focused on malignant tumors (ICD code: C00-C97). A record of cancer diagnosis was needed, and the endpoints were the first diagnosis of these cancers using the ICD-10 codes. In the pan-cancer risk analysis, we excluded all participants with any cancer diagnosis records at baseline, and participants were excluded if they had missing data on covariates. The incident cases were defined as participants who were diagnosed with cancer during the follow-up period. Cancer case records were available through the National Health Service central and death registries. In the mortality analysis, we included participants with a cancer diagnosis (ICD code: C00-C97) at baseline and cancer patients were excluded if they had missing data on covariates. The primary and contributory causes of death were defined based on the ICD-10. All-cause mortality of cancer patients, 5-year cancer mortality, and cancer mortality were defined as the percentage of deaths caused by all-causes, cancer within 5 years (cancer patients with a diagnosis within 2 years prior to enrollment were included, and noncancer deaths were excluded) and cancer within follow-up years (excluding noncancer deaths) among cancer patients, respectively. Participants were followed up from their date of enrollment until the date of diagnosis of malignant tumors, date of withdrawal from the study, date of death, or until the end of follow-up, whichever came first. The definitions and sources of information for outcomes in the United Kingdom Biobank of cancer are shown in Supplementary Table S2.

### Multivariable Cox proportional hazard analysis

Multivariable Cox proportional hazard models were used to investigate the prospective associations of sleep traits with the pan-cancer incidence and mortality among patients with cancer. To minimize the interference of confounding factors, we adjusted for age, sex, assessment center, and the top 10 genetic principal components in the basic model of analyzing pan-cancer incidence and mortality. Additionally, we further adjusted for body mass index, employment status, Townsend deprivation index, smoking status, drinking status and mental health issues, vegetables and fruits intake, sedentary behavior, comorbidity, total physical activity, education, ethnicity and family history in the further adjusted model of analyzing pan-cancer incidence. Similarly, we further adjusted for body mass index, employment status, Townsend deprivation index, smoking status, drinking status, mental health issues, vegetables and fruit intake, sedentary behavior, comorbidity, total physical activity, education, ethnicity, family history, number of self-reported cancers, operation and treatments in a further adjusted model of analyzing mortality. To avoid interference from other sleep traits, we also adjusted for chronotype, sleep duration, and insomnia symptoms in the full model of analyzing pan-cancer incidence and mortality.

### Genetic variants and genetic risk score as instrumental variables

We used 12 SNPs associated with an evening chronotype (Lane et al., 2016). We also used 27 SNPs, 8 SNPs and 78 SNPs that were associated with short sleep duration, long sleep duration and continuous sleep duration, respectively (Dashti et al., 2019). In addition, 57 SNPs were associated with insomnia symptoms (Lane et al., 2019). Detailed information on the SNPs is provided in Supplementary Tables S3–S7. The unweighted GRS (Genetic risk score) was calculated as a summary of the number of risk alleles (0, 1, and 2) across the genetic variants of different sleep traits for each participant (Wang W. et al., 2021).

### Linear mendelian randomization analysis

For linear MR analysis, the genetic variants used were extracted from the United Kingdom Biobank imputation dataset. We used a two-stage method (Richmond et al., 2019; Ai et al., 2021) to assess the causal associations between genetically predicted sleep traits and outcomes by MR analysis. We first regressed the sleep traits on the GRS and then regressed cancer or death status on the fitted values of sleep traits from the first-stage regression, with adjustment for age, sex, assessment centers, top 10 genetic principal components, and genotyping arrays (basic model) in both stages. To eliminate the potential violation of MR assumptions, we tested the associations between potential confounders and GRSs. Then, we repeated our MR analyses with an adjustment of these included confounders in the fully adjusted model. In addition, to avoid the potential interference of other sleep traits, we also analyzed the causal associations after excluding participants with interferential sleep traits, which had strong associations (*p* < 0.05/groups) with the GRSs. For example, we found that insomnia symptoms were strongly associated with the GRSs of short sleep duration, and we excluded participants with insomnia symptoms while exploring the causal association between genetically predicted short sleep duration and the outcomes.

### Nonlinear mendelian randomization analysis

We used nonlinear MR to assess the potential nonlinear J- or U-shaped associations between genetically predicted continuous sleep durations and outcomes. We first divided our sample into three strata based on the residual variation in continuous sleep duration regressed on the GRS (Ai et al., 2021). We then calculated piecewise linear MR effects in each stratum and generated localized average causal effects in these strata. The *p* values from the quadratic test and Cochran’s Q test for nonlinearity are reported (Staley and Burgess, 2017).

### Survival curve analysis

Time-to-event clinical outcomes are common in medical research because they offer more information than simply an event. We used survival analysis methods to handle pan-cancer mortality and censored observations during follow-up. Kaplan‒Meier estimation was used to create survival curves, and the log-rank test was used to compare survival across different groups. Survival curves were also conducted from the Cox models across different groups after adjusting for all covariates.

### Sensitivity analysis

We first performed a sensitivity analysis by removing participants who reported currently working shifts (sometimes, usually, and always) in the multivariable Cox proportional hazard models. Then, participants who had extreme sleep duration (<4 h or >11 h) were removed. We also conducted sensitivity analysis in the multivariable Cox proportional hazard models and survival curve analysis based on age, which only included participants aged >50 years. A quadratic fit test of the results was performed on the association between sleep duration and mortality in cancer patients. To verify the MR assumption that the genetic variants should not be associated with relevant confounders, we investigated associations between allele scores and potential confounders in United Kingdom Biobank. In addition, we used the MR‒Egger, weighted median, and radial MR methods (Bowden et al., 2015; Bowden et al., 2016; Bowden et al., 2018; Ai et al., 2021) to evaluate potential pleiotropy and outliers in the sensitivity analysis. Once outliers were found, they were removed, and the results were reanalyzed. For further sensitivity analysis, we used a Bonferroni-corrected threshold of *p* < 0.05/groups in the MR analysis. *p* values were considered between the Bonferroni-corrected threshold and 0.05 as suggestive evidence in the MR analysis. All statistical analyses were performed using R software (version 4.0.2).

## Results

### Sample characteristics

A total of 326,417 United Kingdom Biobank participants without cancer diagnosis at baseline were included in the pan-cancer incidence analysis, and the detailed screening process is shown in Figure 1. The baseline characteristics of the sample are presented in Table 1. Participants with a cancer diagnosis during the follow-up period were more likely to be male and older, and half of those with a cancer diagnosis were retired or not in the workforce. They were also likely to be previous or current smokers and to have a family history and more comorbidities. Moreover, they had a higher percentage of long sleep duration and frequent insomnia symptoms.

**TABLE 1:** Baseline characteristics of people who had and had not developed cancer during the follow-up period in United Kingdom Biobank (n = 326,417).

Demographics	All Participants		Non-cancer diagnosis		Cancer diagnosis	
**N (%)**	326,417	100.00%	283,516	86.86%	42,901	13.14%
**Sex (n (%))**						
Female	165,914	50.83%	146,968	51.84%	18,946	44.16%
Male	160,503	49.17%	136,548	48.16%	23,955	55.84%
**Age (mean (SD))**	55.86	8.12	55.26	8.11	59.80	6.95
**Body mass index (kg/m** ^**2**^ **) (mean (SD))**	27.26	4.66	27.23	4.67	27.45	4.57
**Socioeconomic status (mean (SD))**	−1.45	3.00	−1.43	3.01	−1.63	2.93
**Mental health issues (n (%))**						
No	290,315	88.94%	251,922	88.86%	38,393	89.49%
Yes	36,102	11.06%	31,594	11.14%	4,508	10.51%
**Smoking status (n (%))**						
Never	180,699	55.36%	159,385	56.22%	21,314	49.68%
Previous	113,065	34.64%	96,000	33.86%	17,065	39.78%
Current	32,653	10.00%	28,131	9.92%	4,522	10.54%
**Alcohol drinker status (n (%))**						
Never	11,880	3.64%	10,529	3.71%	1,351	3.15%
Previous	10,565	3.24%	9,122	3.22%	1,443	3.36%
Current	303,972	93.12%	263,865	93.07%	40,107	93.49%
**Employment status (n (%))**						
In paid employment or self-employed	169,361	51.88%	151,789	53.54%	17,572	40.96%
Retired or not in the workforce	124,809	38.24%	102,510	36.16%	22,299	51.98%
Job involves shift work	32,247	9.88%	29,217	10.30%	3,030	7.06%
**Vegetable and fruit intake (serves/day) (mean (SD))**	4.43	2.94	4.43	2.96	4.39	2.85
**Sedentary behavior (hour/day) (mean (SD))**	4.81	2.43	4.79	2.44	4.91	2.35
**Comorbidity (mean (SD))**	1.76	1.80	1.72	1.78	2.02	1.91
**Total physical activity group (n (%))**						
Low	78,844	24.15%	68,548	24.18%	10,296	24.00%
Moderate	119,587	36.64%	104,018	36.69%	15,569	36.29%
High	127,986	39.21%	110,950	39.13%	17,036	39.71%
**Education level (n (%))**						
Low	190,642	58.40%	164,583	58.05%	26,059	60.74%
High	135,775	41.60%	118,933	41.95%	16,842	39.26%
**Ethnicity (n (%))**						
White	311,068	95.30%	269,176	94.94%	41,892	97.65%
Non-white	15,349	4.70%	14,340	5.06%	1,009	2.35%
**Family history (n (%))**						
No	213,283	65.34%	186,972	65.95%	26,311	61.33%
Yes	113,134	34.66%	96,544	34.05%	16,590	38.67%
**Sleep traits**						
**Chronotype (n (%))**						
Definite morning	79,125	24.24%	68,323	24.10%	10,802	25.18%
More morning	106,682	32.68%	92,550	32.64%	14,132	32.94%
More evening	83,668	25.63%	73,074	25.77%	10,594	24.69%
Definite evening	27,117	8.31%	23,691	8.36%	3,426	7.99%
Unknown/prefer not to answer	29,825	9.14%	25,878	9.13%	3,947	9.20%
**Sleep duration (n (%))**						
≤5 h	15,842	4.85%	13,867	4.89%	1,975	4.60%
6 h	61,683	18.90%	53,994	19.04%	7,689	17.92%
7 h	130,681	40.03%	114,289	40.31%	16,392	38.21%
8 h	94,627	28.99%	81,458	28.73%	13,169	30.70%
≥9 h	23,115	7.08%	19,493	6.88%	3,622	8.44%
Unknown/prefer not to answer	469	0.14%	415	0.15%	54	0.13%
**Insomnia symptom (n (%))**						
Never	84,711	25.95%	74,177	26.16%	10,534	24.55%
Sometimes	154,588	47.36%	134,302	47.37%	20,286	47.29%
Usually	86,999	26.65%	74,939	26.43%	12,060	28.11%
Unknown/prefer not to answer	119	0.04%	98	0.03%	21	0.05%

Sex was categorized as female and male. Mental health issues were categorized as no or yes (had ever seen a doctor or psychiatrist for nerves, anxiety or depression). Smoking status and alcohol drinker status were both categorized as never, previous and current. Employment status was categorized as in paid employment or self-employed, retired or not in the workforce and job involves shift work. Total physical activity (TPA) was categorized into low (<800 MET-min/week), moderate (800–2400 MET-min/week), and high (≥2400 MET, min/week); MET, metabolic equivalent task. Education level was categorized as low or high (college/university degree or other professional qualifications). Ethnicity was categorized as white and non-white. Family history was categorized as no or yes (family history of cancer from father/mother/siblings).

To analyze the relationship between sleep traits and the mortality of cancer patients, a total of 23,956 United Kingdom Biobank participants with a cancer diagnosis at baseline were included in the study, and the detailed screening process is shown in Supplementary Table S8 **.** Their baseline characteristics are listed in Table 2. Patients with cancer who died during the follow-up period were likely to be male, older and smokers, and they had a relatively higher prevalence of mental health issues and comorbidities. They were also likely to be retired or not in the workforce, and had lower vegetables and fruits intakes, increased sedentary behavior, higher BMI, and more treatments. In addition, they had a higher percentage of definite evening chronotype, unfavorable sleep duration and frequent insomnia symptoms.

**TABLE 2:** Baseline characteristics of non-deaths and deaths among cancer patients during the follow-up period in United Kingdom Biobank (n = 23,956).

Demographics	All cancers		Non-deaths		Total deaths	
**N (%)**	23,956	100.00%	20,269	84.61%	3,687	15.39%
**Sex (n (%))**						
Female	13,519	56.43%	11,835	58.39%	1,684	45.67%
Male	10,437	43.57%	8,434	41.61%	2,003	54.33%
**Age (mean (SD))**	53.55	9.32	53.17	9.3	55.64	9.2
**Body mass index (kg/m** ^**2**^ **) (mean (SD))**	27.16	4.61	27.06	4.53	27.73	4.97
**Socioeconomic status (mean (SD))**	−1.66	2.89	−1.75	2.84	−1.20	3.16
**Mental health issues (n (%))**						
No	21,209	88.53%	17,996	88.79%	3,213	87.14%
Yes	2,747	11.47%	2,273	11.21%	474	12.86%
**Smoking status (n (%))**						
Never	12,364	51.61%	10,854	53.55%	1,510	40.95%
Previous	9,561	39.91%	7,893	38.94%	1,668	45.24%
Current	2,031	8.48%	1,522	7.51%	509	13.81%
**Alcohol drinker status (n (%))**						
Never	870	3.63%	716	3.53%	154	4.18%
Previous	914	3.82%	687	3.39%	227	6.16%
Current	22,172	92.55%	18,866	93.08%	3,306	89.67%
**Employment status (n (%))**						
In paid employment or self-employed	8,804	36.75%	7,861	38.78%	943	25.58%
Retired or not in the workforce	13,766	57.46%	11,188	55.20%	2,578	69.92%
Job involves shift work	1,386	5.79%	1,220	6.02%	166	4.50%
**Vegetable and fruit intake (serves/day) (mean (SD))**	4.52	2.96	4.56	2.88	4.32	3.35
**Sedentary behavior (hour/day) (mean (SD))**	4.85	2.28	4.80	2.24	5.13	2.44
**Comorbidity (mean (SD))**	1.98	1.91	1.9	1.86	2.42	2.11
**Total physical activity group (n (%))**						
Low	6,014	25.10%	4,864	24.00%	1,150	31.19%
Moderate	8,636	36.05%	7,402	36.52%	1,234	33.47%
High	9,306	38.85%	8,003	39.48%	1,303	35.34%
**Education level (n (%))**						
Low	14,514	60.59%	12,052	59.46%	2,462	66.78%
High	9,442	39.41%	8,217	40.54%	1,225	33.22%
**Ethnicity (n (%))**						
White	23,505	98.12%	19,896	98.16%	3,609	97.88%
Non-white	451	1.88%	373	1.84%	78	2.12%
**Family history (n (%))**						
No	16,312	68.09%	13,858	68.37%	2,454	66.56%
Yes	7,644	31.91%	6,411	31.63%	1,233	33.44%
**No. of self-reported cancers (mean (SD))**	0.89	0.51	0.87	0.49	0.99	0.58
**Operation (n (%))**						
No	1,593	6.65%	1,360	6.71%	233	6.32%
Yes	22,363	93.35%	18,909	93.29%	3,454	93.68%
**No. of treatments taken (mean (SD))**	2.97	2.86	2.78	2.7	4.06	3.43
**Sleep traits**						
**Chronotype (n (%))**						
Definite morning	5,922	24.72%	4,969	24.52%	953	25.85%
More morning	8,057	33.63%	6,905	34.07%	1,152	31.24%
More evening	6,061	25.30%	5,119	25.26%	942	25.55%
Definite evening	1,815	7.58%	1,512	7.46%	303	8.22%
Unknown/prefer not to answer	2,101	8.77%	1,764	8.70%	337	9.14%
**Sleep duration (n (%))**						
≤5 h	1,206	5.03%	981	4.84%	215	5.83%
6 h	4,160	17.37%	3,489	17.21%	671	18.20%
7 h	8,712	36.37%	7,527	37.14%	1,185	32.14%
8 h	7,462	31.15%	6,386	31.51%	1,076	29.18%
≥9 h	2,376	9.92%	1,846	9.11%	530	14.37%
Unknown/prefer not to answer	40	0.17%	30	0.15%	10	0.27%
**Insomnia symptom (n (%))**						
Never	5,261	21.96%	4,540	22.40%	721	19.56%
sometimes	11,153	46.56%	9,515	46.94%	1,638	44.43%
Usually	7,536	31.46%	6,209	30.63%	1,327	35.99%
Unknown/prefer not to answer	6	0.03%	5	0.02%	1	0.03%

Sex was categorized as female and male. Mental health issues were categorized as no or yes (had ever seen a doctor or psychiatrist for nerves, anxiety or depression). Smoking status and alcohol drinker status were both categorized as never, previous and current. Employment status was categorized as in paid employment or self-employed, retired or not in the workforce and job involves shift work. Total physical activity (TPA) was categorized into low (<800 MET-min/week), moderate (800–2400 MET-min/week), and high (≥2400 MET, min/week); MET, metabolic equivalent task. Education level was categorized as low or high (college/university degree or other professional qualifications). Ethnicity was categorized as white and non-white. Family history was categorized as no or yes (family history of cancer from father/mother/siblings). Operation was categorized as no or yes.

### Multivariable Cox proportional hazard analysis

**FIGURE 2:**
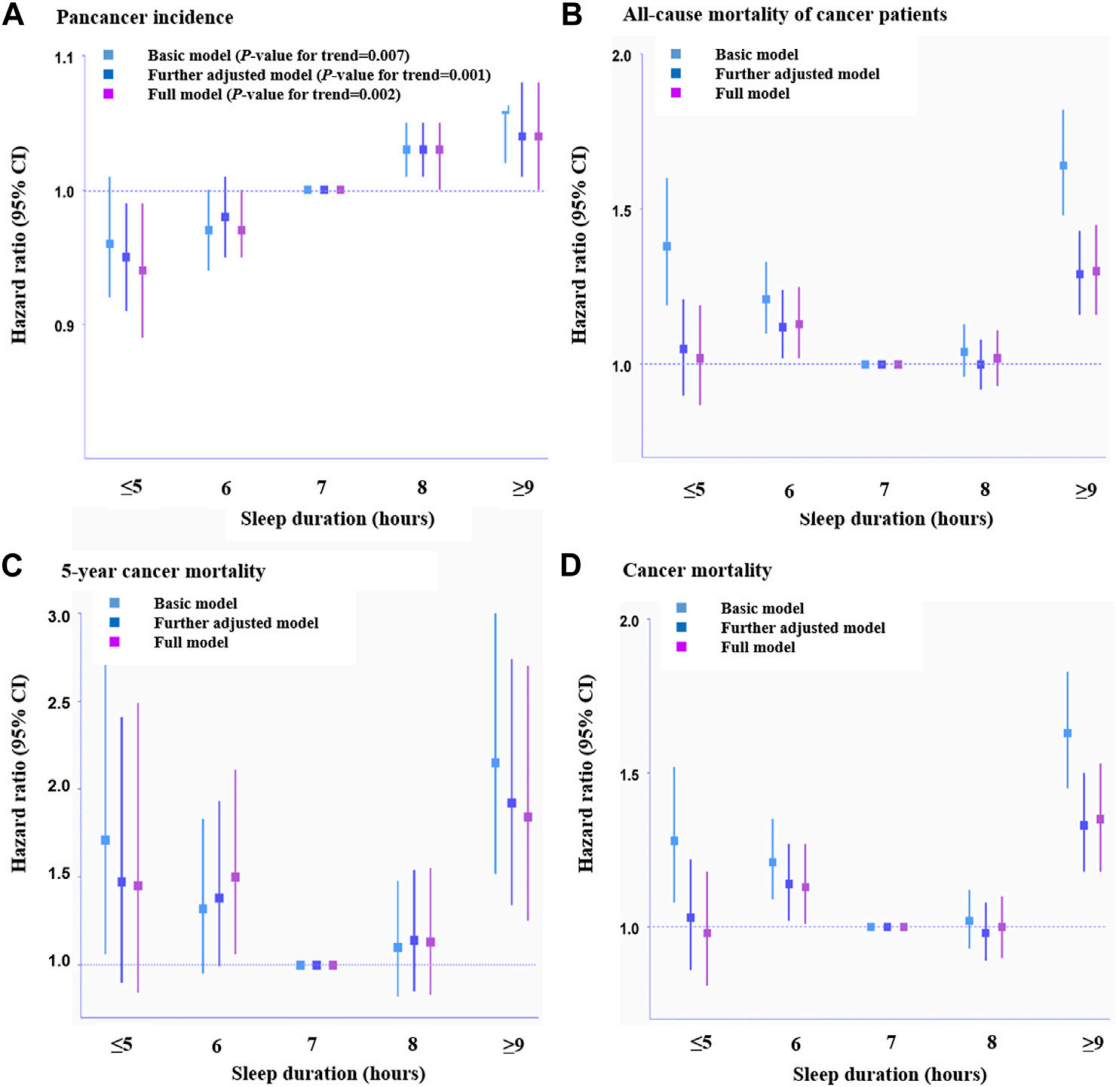
Association of sleep duration with pan-cancer incidence and mortality among cancer patients across different hours. **(A)**. Pan-cancer incidence (n = 326,417), **(B)**. All-cause mortality of cancer patients (n = 23,956), **(C)**. 5-Year cancer mortality (n = 4,962), and **(D)**. Cancer-caused mortality (n = 23,133). HRs across hours of habitual sleep duration were presented with 7 h serving as a reference group. Error bars are 95% CI. Basic model: adjusted for age, sex, assessment center, top 10 genetic principal components and genotyping array. Further adjusted model: adjusted for age, sex, assessment center, top 10 genetic principal components, genotyping array, body mass index, employment status, Townsend deprivation index, smoking status, drinking status and mental health issues, vegetables and fruit intake, sedentary behavior, comorbidity, total physical activity, education, ethnicity, family history, number of self-reported cancers, operation and treatments. Full adjusted model: adjusted for age, sex, assessment center, top 10 genetic principal components, genotyping array, body mass index, employment status, Townsend deprivation index, smoking status, drinking status and mental health issues, vegetables and fruit intake, sedentary behavior, comorbidity, total physical activity, education, ethnicity, family history, number of self-reported cancers, operation, treatments and other sleep traits. Statistical significance was defined as *p* < 0.05.

There was no association between definite evening chronotype and pan-cancer incidence after adjusting for demographic information and other sleep traits (HR, 1.03, 95% CI: 0.99–1.07, *p* = 0.137; Supplementary Table S9). However, we observed a positive association between sleep duration and pan-cancer incidence after adjusting for all the covariates (HR, 0.94, 95% CI: 0.89–0.99, *p* = 0.016, sleep duration≤5 h; HR, 0.97, 95% CI: 0.94–1.00, *p* = 0.038, sleep duration = 6 h; HR, 1.03, 95% CI: 1.00–1.05, *p* = 0.021, sleep duration = 8 h; HR, 1.04, 95% CI: 1.00–1.08, *p* = 0.031, sleep duration≥9 h; Supplementary Table S9). Thus, there could be a linear association between sleep duration and pan-cancer incidence (Figure 2A). In addition, there was a positive association between frequent insomnia symptoms and pan-cancer incidence in the fully adjusted model (HR, 1.03, 95% CI: 1.00–1.06, *p* = 0.035; Supplementary Table S9).

Regarding the relationship between chronotype and mortality (all-cause mortality, 5-year cancer mortality, and cancer mortality) among cancer patients, no association between definite evening chronotype and mortality of cancer patients was found (HR, 0.95, 95% CI: 0.83–1.08, *p* = 0.450, all-cause mortality, Supplementary Table S10; HR, 0.89, 95% CI: 0.56–1.43, *p* = 0.638, 5-year cancer mortality, Supplementary Table S11; HR, 0.91, 95% CI: 0.79–1.06, *p* = 0.241, cancer mortality, Supplementary Table S12). A positive association between shorter sleep duration (6 h) and all-cause mortality (HR, 1.13, 95% CI: 1.02–1.25, *p* = 0.019, Supplementary Table S10), 5-year cancer mortality (HR, 1.50, 95% CI: 1.06–2.22, *p* = 0.022, Supplementary Table S11) and cancer mortality (HR, 1.13, 95% CI: 1.01–1.27, *p* = 0.033, Supplementary Table S12) among cancer patients was observed in this study. Meanwhile, we also found a positive association between long sleep duration (≥9 h) and all-cause mortality (HR, 1.30, 95% CI: 1.16–1.45, *p* < 0.001, Supplementary Table S10), 5-year cancer mortality (HR, 1.84, 95% CI: 1.25–2.70, *p* = 0.002, Supplementary Table S11) and cancer mortality (HR, 1.35, 95% CI: 1.18–1.53, *p* < 0.001, Supplementary Table S12) among cancer patients. This indicated that there was a J-shaped association between sleep duration and mortality among cancer patients (Figures 2B–D). In addition, there was a positive association between frequent insomnia symptoms and all-cause mortality (HR, 1.17, 95% CI: 1.06–1.30, *p* = 0.002, Supplementary Table S10) and cancer mortality among cancer patients (HR, 1.25, 95% CI: 1.11–1.41, *p* < 0.001, Supplementary Table S12). However, there was no association between frequent insomnia symptoms and 5-year cancer mortality among cancer patients (HR, 1.14, 95% CI: 0.80–1.63, *p* = 0.475, Supplementary Table S11).

### Linear mendelian randomization analysis

**FIGURE 3:**
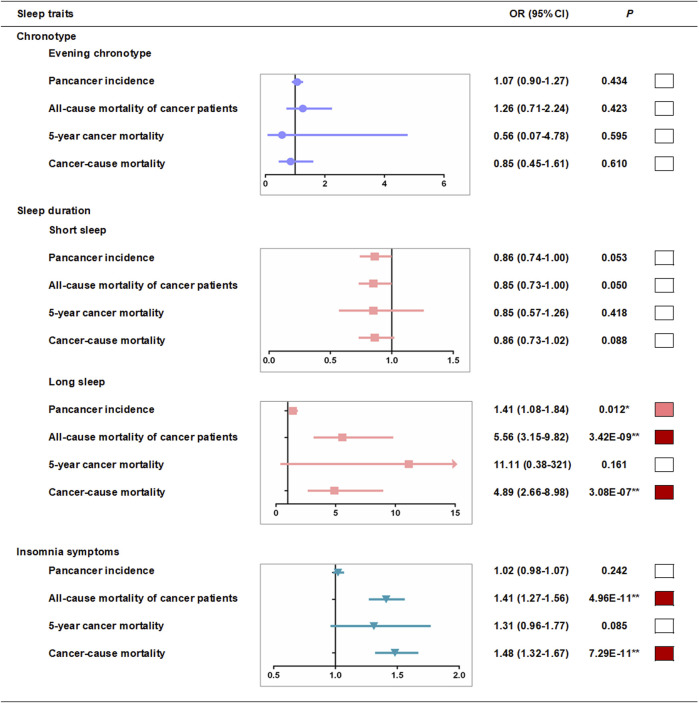
Linear-MR estimates for association between sleep traits and pan-cancer incidence and mortality among cancer patients. Odds ratios are per category in evening chronotype (definite morning [incidence, n = 79,023; all-cause mortality, n = 5,914; 5-year mortality, n = 1,235; cancer mortality, n = 5,703], intermediate morning [106,528; 8,047; 1,711; 7,813], intermediate evening [83,534; 6,045; 1,209; 5,811], and definite evening [27,034; 1,813; 358; 1,738]), per category in sleep duration (≤5 h [1,349; 1,099; 216; 1,046], 6 h [9,997; 3,845; 774; 3,714], 7 h [31,159; 7,905; 6,800; 2,170], 8 h [27,403; 6,800; 1,454; 6,565], ≥9 h [6,401; 2,170; 438; 2,056] in short sleep duration MR analysis and ≤5 h [9,161; 1,099; 216; 1,046], 6 h [35,570; 3,845; 774; 3,714], 7 h [73,791; 7,905; 6,800; 2,170], 8 h [54,817; 6,800; 1,454; 6,565], ≥9 h [12,212; 2,170; 438; 2,056] in long sleep duration analysis) and per category in insomnia risk (no [64,963; 4,729; 1,003; 4,566], some [115,628; 10,192; 2,127; 9,868], and frequent [45,165; 6,898; 1,383; 6,631] insomnia symptoms). ■*p < 0.05 and ■**P < bonferroni-corrected threshold of 0.05/groups. Further adjusted model adjusted for age, sex, assessment centers, top 10 genetic principal components, genotyping array and GRS association factors.

We used a two-stage least squares method to assess the associations between genetically predicted sleep traits and outcomes produced by the linear MR analyses. We first regressed the exposures on the genetic risk scores (GRSs) of sleep traits (Supplementary Tables S13–16), and then we regressed the outcome on the fitted values of the exposure from the first-stage regression (Supplementary Tables S17, 18). In the first-stage regression, no obvious associations of evening chronotype GRSs with other sleep traits were found (Supplementary Table S13), and we observed that there was no association between definite evening chronotype and pan-cancer incidence in the fully adjusted model of linear MR analysis (OR, 1.09, 95% CI: 0.92–1.30 per category increase, *p* = 0.306) (Supplementary Table S17 and Figure 3). However, a strong association (*p* < 0.05/24 groups = 2.08E-03) of short sleep duration GRSs with insomnia symptoms (Supplementary Table S14) was found in the pan-cancer incidence samples in the first-stage regression. To avoid the potential interference of other sleep traits, we excluded participants with insomnia symptoms and then repeated the MR analyses in the pan-cancer incidence samples and found that there was no association between short sleep duration and pan-cancer incidence in the fully adjusted model of linear MR analysis (OR, 0.86, 95% CI: 0.74–1.00 per category increase, *p* = 0.053) (Supplementary Table S18 and Figure 3). Meanwhile, the strong association (*p* < 0.05/24 groups = 2.08E-03) of long sleep duration GRSs with evening chronotype (Supplementary Table S16) was also found in the pan-cancer incidence samples in the first-stage regression. Hence, we excluded participants with the evening chronotype and then repeated the MR analyses in the pan-cancer incidence samples (Supplementary Table S18). After excluding participants with the evening chronotype, there was still supporting evidence of positive associations between long sleep duration and pan-cancer incidence (OR, 1.41, 95% CI: 1.08–1.84 per category increase, *p* = 0.012) (Supplementary Table S18 and Figure 3) in the fully adjusted model of linear MR analysis. In addition, a strong association (*p* < 0.05/24 groups = 2.08E-03) of insomnia symptom GRSs with short sleep duration (Supplementary Table S16) was found in the pan-cancer incidence samples in the first-stage regression. After excluding participants with short sleep duration, there was also no association between insomnia symptoms and pan-cancer incidence in the fully adjusted model of linear MR analysis (OR, 1.02, 95% CI: 0.98–1.07 per category increase, *p* = 0.242) (Supplementary Table S18 and Figure 3).

Similarly, we tested the associations between potential confounders and the sleep trait GRSs in cancer mortality samples, and no obvious association of sleep trait GRSs with other sleep traits in the cancer mortality samples was found (Supplementary Tables S19–22). Linear MR analyses suggested that there was a positive association between long sleep duration and all-cause mortality in cancer patients (OR, 5.56, 95% CI: 3.15–9.82 per category increase, *p* = 3.42E-09, Supplementary Table S24 and Figure 3) and a strong association between frequent insomnia symptoms and all-cause mortality in cancer patients (OR, 1.41, 95% CI: 1.27–1.56 per category increase, *p* = 4.96E-11, Supplementary Table S25 and Figure 3) in the fully adjusted model. We also observed a positive association between long sleep duration and cancer mortality in cancer patients (OR, 4.89, 95% CI: 2.66–9.82 per category increase, *p* = 3.08E-07, Supplementary Table S24 and Figure 3) and a strong association between frequent insomnia symptoms and cancer mortality in cancer patients (OR, 1.48, 95% CI: 1.32–1.67 per category increase, *p* = 7.29E-11, Supplementary Table S25 and Figure 3) in the fully adjusted linear MR analysis. However, we found that there was no association between long sleep duration (OR, 11.11, 95% CI: 0.38–321 per category increase, *p* = 0.161, Supplementary Table S24 and Figure 3), insomnia symptoms (OR, 1.31, 95% CI: 0.96–1.77 per category increase, *p* = 0.085, Supplementary Table S24 and Figure 3) and 5-year cancer mortality in the fully adjusted model of linear MR analysis. In addition, we found that there was no association between definite evening chronotype (OR, 1.26, 95% CI: 0.71–2.24 per category increase, *p* = 0.423, all-cause mortality, Supplementary Table S23 and Figure 3; OR, 0.56, 95% CI: 0.07–4.78 per category increase, *p* = 0.595,5-year cancer mortality, Supplementary Table S23 and Figure 3; OR, 0.85, 95% CI: 0.45–1.61 per category increase, *p* = 0.610, cancer mortality, Supplementary Table S23 and Figure 3) and the mortality of cancer patients in the fully adjusted model of linear MR analysis. There was also no association between short sleep duration (OR, 0.85, 95% CI: 0.73–1.00 per category increase, *p* = 0.050, all-cause mortality, Supplementary Table S24 and Figure 3; OR, 0.85, 95% CI: 0.57–1.26 per category increase, *p* = 0.419, 5-year cancer mortality, Supplementary Table S24 and Figure 3; OR, 0.86, 95% CI: 0.73–1.02 per category increase, *p* = 0.088, cancer mortality, Supplementary Table S24 and Figure 3) and mortality of cancer patients in the fully adjusted model of the linear MR analysis.

### Nonlinear mendelian randomization analysis

We observed no evidence favoring a nonlinear relationship between genetically continuous sleep duration and the study outcomes (pan-cancer incidence, all-cause mortality in cancer patients, 5-year cancer mortality, and cancer mortality**;** Supplementary Table S26). To avoid the potential interference of extreme sleep duration (<4 h or >11 h), there was still no statistical evidence for nonlinear associations between genetically predicted continuous sleep durations and the risk of cancer and mortality using the piecewise linear method after excluding participants with extreme sleep duration (Supplementary Table S27). This means that there were no potential nonlinear J- or U-shaped associations between genetically predicted continuous sleep duration and outcomes.

### Mortality and survival curve analysis

**FIGURE 4:**
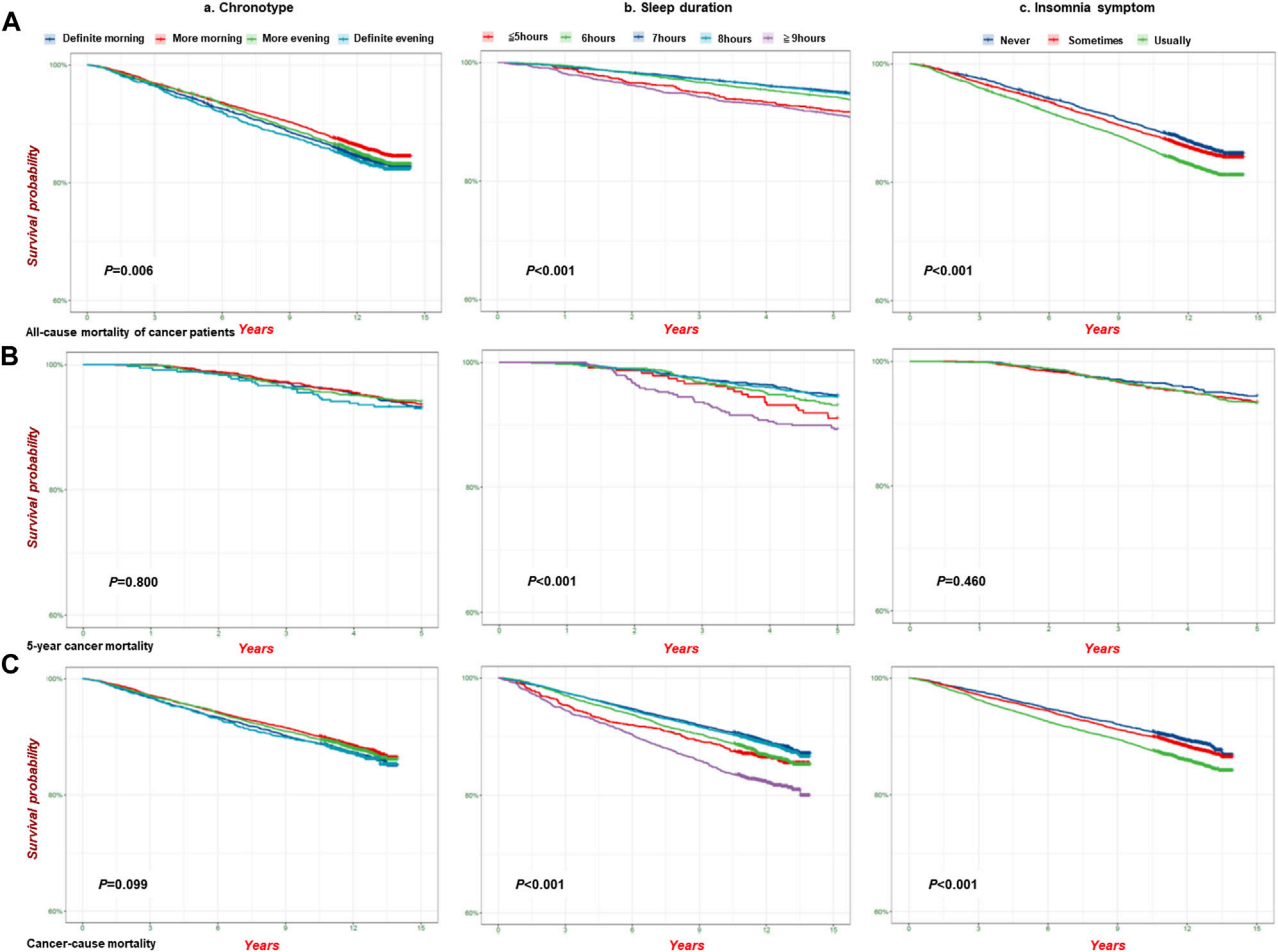
The analysis of survival probability of sleep traits on pan-cancer patients in the UKB cohort. Kaplan–Meier plots for the survival probability of pan-cancer patients in different group of sleep traits. **(A)** all-cause mortality (n = 23,956); **(B)** 5-Year cancer mortality (n = 4,962); **(C)**. Cancer-cause mortality (n = 23,133) in the UKB cohort. *P* calculated by the log-rank test, and *p* < 0.05 for trend across different groups. **(a)**. Chronotype; **(b)**. Sleep duration; **(c)**. Insomnia.

We also conducted an analysis of mortality among cancer patients (all-cause mortality of cancer patients, 5-year cancer mortality, and cancer mortality) associated with sleep traits and found that the highest mortality occurred in the definite evening chronotype, long sleep duration (≥9 h), and insomnia symptom subgroups (Supplementary Table S28). In addition, Figure 4 shows the analysis of the survival probability of sleep traits with *p* < 0.05 across different groups. The lowest survival probability occurred in the definite evening chronotype, long sleep duration (≥9 h), and insomnia symptoms subgroups in all-cause mortality (Figure 4A), 5-year cancer mortality (Figure 4B) and cancer-cause mortality (Figure 4C) among cancer patients, respectively. We also observed a similar survival curve from the Cox models by adjusting for additional covariates (Supplementary Table S29).

### Sensitivity analyses

In the sensitivity analyses, we first removed participants who reported currently working shifts (sometimes, usually, and always) and participants who had extreme sleep durations (<4 h or >11 h). Then, we obtained the same results as in the fully adjusted model before the removal (Supplementary Tables S30, S31). We also conducted a sensitivity analysis based on age>50, and the multivariable Cox regression results for pan-cancer incidence and mortality associated with sleep traits were similar (Supplementary Table S32). Considering the underlying impact of age on survival probability, we conducted a stratified analysis and obtained similar results in the survival probability of sleep traits in cancer patients (age>50 years) (Supplementary Table S33, S34). After adjusting for potential confounders and other sleep traits that were potentially associated with GRSs (Supplementary Tables S13–S16 and Supplementary Tables S19–S22), the results were generally consistent with the basic model in the linear MR analysis (Supplementary Tables S17, S18 and Supplementary Tables S23–S25). Furthermore, we excluded those with interferential sleep traits that had strong associations with GRSs (*p* < 0.05/24 groups = 2.08E-03) and then repeated the linear and nonlinear MR analyses (Supplementary Tables S18–S27). In addition, we used a Bonferroni-corrected threshold in our MR analysis to indicate a strong association. We also performed the test for a quadratic fit of the results on the association between sleep duration and mortality of cancer patients (Figures 2B–D) (Supplementary Tables S35–S37). Scatter plots of individual single nucleotide polymorphisms (SNPs) of sleep traits and SNP effects on pan-cancer incidence and mortality among patients with cancer are shown in Supplementary Tables S38–S41. Radial MR analysis (Bowden et al., 2015; Bowden et al., 2016; Bowden et al., 2018; Ai et al., 2021) was used to identify outlying genetic variants in pan-cancer incidence and mortality among cancer patients (Supplementary Tables S42–S45). An MR leave-one-out sensitivity analysis for the effect of the sleep trait SNPs on outcomes was also conducted (Supplementary Tables S46–S49). Moreover, we used MR‒Egger and weighted median analyses to account for any potential pleiotropy (Supplementary Tables S50–S52), and the results showed that the SNPs used for instrumental variables had no obvious pleiotropy.

## Discussion

The present study found that long sleep duration and frequent insomnia symptoms were associated with a higher pan-cancer incidence, and long sleep duration had a causal relationship with the incidence of pan-cancer. Among cancer patients, we observed that long sleep duration and frequent insomnia symptoms were relevant risk factors and even potential causal risk factors for mortality. The highest mortality could occur in the definite evening chronotype, long sleep duration, and frequent insomnia symptoms subgroups. The survival analysis confirmed that the lowest survival probability could occur in the definite evening chronotype, long sleep duration, and frequent insomnia symptoms subgroups. In summary, this study provided convincing evidence of the causal relationships between multiple sleep traits and pan-cancer incidence and mortality among cancer patients through the use of multiple methods (**Graphical Abstract**) and might help in the management of cancer based on sleep behaviors.

Sleep duration has been found to be associated with cancer risk, including breast, lung, and prostate cancer (Richmond et al., 2019; Wang J. et al., 2021; Tao et al., 2021; Xie et al., 2021; Peeri et al., 2022; Wilunda et al., 2022). A U-shaped association was observed between sleep duration and lung cancer risk (Xie et al., 2021). A MR study observed suggestive evidence of a causal association between both short and long sleep duration and risk of some site-specific cancers (Titova et al., 2021). However, our study found a covariate relationship between short sleep duration and frequent insomnia symptoms, and therefore, we adjusted for multiple sleep traits with possible covariates instead of individual sleep trait in the analysis model. Finally, our study confirmed that long sleep duration was associated with a higher cancer incidence, which was consistent with several studies (Tao et al., 2021; Peeri et al., 2022; Wilunda et al., 2022). Furthermore, this study (Titova et al., 2021) only explored the causal relationship between sleep duration and cancer risk, and it did not consider the influence of other sleep traits on cancer risk and mortality. Thus, our study provided more evidence for the relationship between several sleep traits and cancer risk. In addition, inconsistent results have been reported regarding the relationship between insomnia and cancer risk (Sen et al., 2017; Richmond et al., 2019; Wang J. et al., 2021; Huo et al., 2021; Peeri et al., 2022). In the present study, we found that frequent insomnia symptoms were associated with a higher pan-cancer incidence in the Cox proportional hazard analysis, which was also confirmed by other studies (Sen et al., 2017; Peeri et al., 2022). However, the causal association of frequent insomnia symptoms with pan-cancer incidence was not found by linear MR analysis, which was consistent with the findings of another study (Richmond et al., 2019). This indicates that there is no causal relationship between insomnia symptoms and pan-cancer risk and that insomnia symptoms might be related to the increased pan-cancer incidence by interfering with multiple factors. Many conventional observational studies suggest that the evening chronotype is associated with an increased risk of some types of cancer (Erren et al., 2015; Hurley et al., 2019; Richmond et al., 2019; Wang J. et al., 2021; Sun et al., 2021; Von Behren et al., 2021; Xie et al., 2021; Costas et al., 2022; Peeri et al., 2022; Yu et al., 2022; Yuan et al., 2023). However, only a few studies analysed the causal association between chronotype and cancer risk (Richmond et al., 2019; Wang J. et al., 2021; Sun et al., 2021; Yu et al., 2022; Yuan et al., 2023). Our study explored the correlations and causal associations between evening chronotype and pan-cancer risk using multivariable Cox proportional hazard and MR analyses simultaneously. However, definite evening chronotype could not increase the pan-cancer incidence in the multivariable Cox proportional hazard analysis, which was also confirmed by the MR analysis.

Our study found consistent results for the association between unfavorable sleep duration, especially long sleep duration and mortality among cancer patients in both the multivariable Cox proportional hazard and MR analyses. Several cohort studies have revealed a relationship between sleep duration and mortality (Yeo et al., 2013; Xiao et al., 2014; Tao et al., 2021; Wilunda et al., 2022). However, no study has focused on the population with cancer. Only when focusing on patients with cancer can we accurately elucidate the effects of sleep duration on cancer mortality. Other sleep traits, such as insomnia (Li et al., 2014; Bertisch et al., 2018; Garfield et al., 2019; Hedstrom et al., 2019; Sogawa et al., 2022), are associated with all-cause mortality, cardiovascular disease mortality, and cancer mortality, which could support our findings that frequent insomnia symptoms were a relevant risk factor and even a potential causal risk factor for mortality among cancer patients. However, the results were usually confounded by self-reported exposures and residual confounding effects in previous studies, and MR analysis methods could overcome this limitation. In addition, although several studies have explored the association between chronotype and mortality (Dickerman et al., 2016; Knutson and von Schantz, 2018; Quinn et al., 2022), few studies have focused on the causal relationship between chronotype and cancer mortality. Our study observed the phenotypic association between the definite evening chronotype and mortality, although no causal association of definite evening chronotype with mortality was found.

The current study integrated multiple analyses, including multivariable Cox proportional hazard analysis, survival curve analysis, linear MR analysis, and nonlinear MR analysis, to explore the correlations and causal relationships of multiple sleep traits (chronotype, sleep duration, and insomnia symptoms) on pan-cancer incidence and mortality in the United Kingdom Biobank cohort. In the multivariable Cox proportional hazard analysis, we used the basic, further adjusted, and fully adjusted models. However, some confounding factors, including measurement error, selection bias, and unmeasured factors, might reverse the effect estimates. Therefore, we used MR analysis to mitigate potential bias. We used genetic instruments of sleep traits identified in the GWAS with a Bonferroni-corrected statistical significance threshold as to repeat the results. Considering the inconsistent results of sleep duration and the study outcomes, we also used nonlinear MR analysis to further explore the causal relationship between sleep duration and pan-cancer incidence and mortality among cancer patients. Additionally, we conducted a series of sensitivity analyses to assess the main assumptions and results.

This study had several limitations. First, the baseline characteristics of the stage, grade, and treatment of cancers were unavailable, which could restrict the exploration of their potential influence on the results. However, we included several relevant variables, including the number of treatments/medications and operations among cancer patients to decrease bias. Second, self-reported measures were used in the multivariable Cox proportional hazard analysis rather than objective measures. However, previous studies suggest good concordance between self-reported measures and accelerometer measures in United Kingdom Biobank (Dashti et al., 2019; Jones et al., 2019). Third, the pan-cancer risk increased was small with *p* values close to borderline in the multivariable Cox proportional hazard analysis. However, it is still statistically significant, and therefore, it is still relevant. Fourth, the genetic variants are associated with some confounders, such as body mass index, which might be affected by pleiotropy. However, the results were consistent with those of the primary analyses after further adjustment for potential confounders. In addition, we repeated the analysis after excluding participants with interfering sleep traits. However, the limited number of SNPs associated with a definite evening chronotype and long sleep duration may cause these results to be biased due to weak instrumental variables. Weak instrumental variables could affect the direction of the observational association (Burgess et al., 2016). In most observational studies, the associations of being an evening chronotype or having a long sleep duration with cancer were largely consistent across different methods (Xie et al., 2021; Lotti et al., 2022; Peeri et al., 2022). Therefore, bias should be minimal. Finally, we used unweighted genetic risk scores to minimize bias caused by potentially weak instruments in the MR analysis.

In summary, long sleep duration, and frequent insomnia symptoms were associated with a higher pan-cancer risk, and long sleep duration had a causal relationship with pan-cancer incidence. Among cancer patients, long sleep duration and frequent insomnia symptoms were relevant and potential causal risk factors for mortality, respectively. The present study also detected the highest risk of death and the lowest survival probability in unfavorable sleep trait subgroups, which further emphasizes the effect of unfavorable sleep traits on mortality outcomes in cancer patients. Therefore, attention should be given not only to the relationship between sleep issues and the risk of cancer in society but also to cancer patients with sleep issues to manage the prognosis of patients with cancer.

## Data Availability

The datasets presented in this study can be found in online repositories. The names of the repository/repositories and accession number(s) can be found in the article/Supplementary Material.
