# Supplementary material for: Causal associations of sleep traits with cancer incidence and mortality
Source: Front Genet. 2023 Nov 23;14:1309069. doi: 10.3389/fgene.2023.1309069 (PMC10710159; doi:10.3389/fgene.2023.1309069)
Supplement: Supplementary file 1 [file Table1.DOCX]

**Supplementary Tables**

**Contents**

**Table S1. The descriptions and sources of selected covariates, genotyping process and sample quality control in UK Biobank.**

**Table S2. The descriptions and sources of information for outcomes in UK Biobank.**

**Table S3. 12 single-nucleotide polymorphisms list of evening chronotype identified in UK Biobank.**

**Table S4. 27 single-nucleotide polymorphisms list of short sleep duration identified in UK Biobank.**

**Table S5. 8 single-nucleotide polymorphisms list of long sleep duration identified in UK Biobank.**

**Table S6. 78 single-nucleotide polymorphisms list of continuous sleep duration identified in UK Biobank.**

**Table S7. 57 single-nucleotide polymorphisms list of insomnia identified in UK Biobank.**

**Table S8. The flow chart of cancer patients for analyzing mortality.**

**Table S9. Multivariable Cox regression analysis for the pancancer incidence associated with sleep traits.**

**Table S10. Multivariable Cox regression analysis for the all-cause mortality among cancer patients associated with sleep traits.**

**Table S11. Multivariable Cox regression analysis for the cancer mortality among cancer patients associated with sleep traits in the 5 years.**

**Table S12. Multivariable Cox regression analysis for the cancer mortality associated with sleep traits among cancer patients during the follow-up years.**

**Table S13. Associations between evening chronotype unweighted GRS and potential confounders among pancancer incidence samples in UK Biobank (N=296,119).**

**Table S14. Associations between short sleep duration unweighted GRS and potential confounders among pancancer incidence samples in UK Biobank (N=296,119).**

**Table S15. Associations between long sleep duration unweighted GRS and potential confounders among pancancer incidence samples in UK Biobank (N=296,119).**

**Table S16. Associations between insomnia unweighted GRS and potential confounders among pancancer incidence samples in UK Biobank (N=296,119).**

**Table S17. Mendelian randomization estimates for genetically predicted sleep traits with adjustment for other confounders (pancancer incidence).**

**Table S18. Mendelian randomization estimates for genetically predicted sleep traits with adjustment for other confounders after excluding the disturbed participants (pancancer incidence).**

**Table S19. Associations between evening chronotype unweighted GRS and potential confounders among cancer samples in UK Biobank (N=21,819).**

**Table S20. Associations between short sleep duration unweighted GRS and potential confounders among cancer samples in UK Biobank (N=21,819).**

**Table S21. Associations between long sleep duration unweighted GRS and potential confounders among cancer samples in UK Biobank (N=21,819).**

**Table S22. Associations between long sleep duration unweighted GRS and potential confounders among cancer samples in UK Biobank (N=21,819).**

**Table S23. Mendelian randomization estimates for genetically predicted evening chronotype with adjustment for other confounders (mortality).**

**Table S24. Mendelian randomization estimates for genetically predicted short sleep duration and long sleep duration with adjustment for other confounders (mortality).**

**Table S25. Mendelian randomization estimates for genetically predicted insomnia symptom with adjustment for other sleep traits confounders (mortality).**

**Table S26. Non-linear Mendelian randomization results between genetically predicted continuous sleep durations and pancancer incidence and mortality among cancer patients using piecewise linear method.**

**Table S27. Non-linear Mendelian randomization results between genetically predicted continuous sleep durations and pancancer incidence and mortality among cancer patients using piecewise linear method (participants with extremely sleep duration were excluded).**

**Table S28. Analysis for mortality of cancer associated with sleep traits.**

**Table S29. The analysis of survival probability of sleep traits on cancer patients among the UKB cohort (additional adjustment of full covariates).**

**Table S30. Multivariable Cox regression analysis for pancancer incidence and mortality associated with sleep traits among cancer patients after removing participants who reported currently working shifts.**

**Table S31. Multivariable Cox regression analysis for pancancer incidence and mortality associated with sleep traits among cancer patients after removing participants who reported extremely sleep duration (<4h or >11h).**

**Table S32. Multivariable Cox regression analysis for pancancer incidence and mortality associated with sleep traits among age > 50 cancer patients.**

**Table S33. The analysis of survival probability of sleep traits on pancancer patients (age>50 years) among the UKB cohort.**

**Table S34. The analysis of survival probability of sleep traits on pancancer patients (age>50 years) among the UKB cohort (additional adjustment of full covariates).**

**Table S35. The test for a quadratic fit of the results on association between sleep duration and all-cause mortality among cancer patients (Figure 2b).**

**Table S36. The test for a quadratic fit of the results on association between sleep duration and 5-year cancer mortality among cancer patients (Figure 2c).**

**Table S37. The test for a quadratic fit of the results on association between sleep duration and cancer-cause mortality among cancer patients (Figure 2d).**

**Table S38. Scatter plot of individual SNP-sleep traits and SNP effect on pancancer incidence associations with overlay of causal estimate from each MR test using.**

**Table S39. Scatter plot of individual SNP-sleep traits and SNP effect on all-cause mortality of cancer patient associations with overlay of causal estimate from each MR test using.**

**Table S40. Scatter plot of individual SNP-sleep traits and SNP effect on 5-year cancer mortality of cancer patient associations with overlay of causal estimate from each MR test using.**

**Table S41. Scatter plot of individual SNP-sleep traits and SNP effect on cancer mortality of cancer patient associations with overlay of causal estimate from each MR test using.**

**Table S42. Radial MR plots for sleep traits in pancancer incidence.**

**Table S43. Radial MR plots for sleep traits in all-cause mortality among cancer patients.**

**Table S44. Radial MR plots for sleep traits in 5-year cancer mortality among cancer patients.**

**Table S45. Radial MR plots for sleep traits in cancer-cause mortality among cancer patients.**

**Table S46. MR leave-one-out sensitivity analysis for the effect of the evening chronotype SNPs on outcomes.**

**Table S47. MR leave-one-out sensitivity analysis for the effect of the short sleep duration SNPs on outcomes.**

**Table S48. MR leave-one-out sensitivity analysis for the effect of the long sleep duration SNPs on outcomes.**

**Table S49. MR leave-one-out sensitivity analysis for the effect of the insomnia SNPs on outcomes.**

**Table S50. Sensitivity analysis for evening chronotype and pancancer incidence and mortality of cancer patients using the Weighted median and MR-Egger methods.**

**Table S51. Sensitivity analysis for short/long sleep duration and pancancer incidence and mortality of cancer patients using the Weighted median and MR-Egger methods.**

**Table S52. Sensitivity analysis for insomnia and pancancer incidence and mortality of cancer patients using the Weighted median and MR-Egger methods.**

**Table S1. The descriptions and sources of selected covariates, genotyping process and sample quality control in UK Biobank.**

| **Field ID** | **Description** | **Category** |
| --- | --- | --- |
| 31 | Sex | Baseline characteristics |
| 21022 | Age at recruitment | Baseline characteristics |
| 21001 | Body mass index (BMI) | Body size measures |
| 189 | Townsend deprivation index at recruitment | Baseline characteristics |
| 2100 | Seen a psychiatrist for nerves, anxiety, tension or depression | Mental health |
| 20116 | Smoking status | Smoking |
| 6142 | Current employment status | Employment |
| 3426 | Job involves night shift work | Employment |
| 826 | Job involves shift work | Employment |
| 20117 | Alcohol drinker status | Alcohol |
| 1180 | Morning/evening person (chronotype) | Sleep |
| 1160 | Sleep duration | Sleep |
| 1200 | Insomnia | Sleep |
| 191 | Date lost to follow-up | Ongoing characteristics |
| 53 | Date of attending assessment center | Reception |
| 54 | UK Biobank assessment center | Reception |
| 1299 | Salad / raw vegTable Sintake | Diet |
| 1309 | Fresh fruit intake | Diet |
| 1070 | Time spent watching television (TV) | Physical activity |
| 1080 | Time spent using computer | Physical activity |
| 1090 | Time spent driving | Physical activity |
| 864 | Number of days/week walked 10+ minutes | Physical activity |
| 874 | Duration of walks | Physical activity |
| 884 | Number of days/week of moderate physical activity 10+ minutes | Physical activity |
| 894 | Duration of moderate activity | Physical activity |
| 904 | Number of days/week of vigorous physical activity 10+ minutes | Physical activity |
| 914 | Duration of vigorous activity | Physical activity |
| 6138 | Qualifications | Education |
| 21000 | Ethnicity background | Ethnicityity |
| 20107 | Illnesses of father | Family history |
| 20110 | Illnesses of mother | Family history |
| 20111 | Illnesses of siblings | Family history |
| 135 | Number of self-reported non-cancer illnesses | Medical conditions |
| 22027 | Outliers for heterozygosity or missing rate | Genotyping process and sample QC |
| 22019 | Sex chromosome aneuploidy | Genotyping process and sample QC |
| 22021 | Genetic kinship to other participants | Genotyping process and sample QC |
| 22009 | Genetic principal components | Genotyping process and sample QC |

**Table S2. The descriptions and sources of information for outcomes in UK Biobank.**

| **Field ID** | **Description** | **Category** |
| --- | --- | --- |
| 84 | Cancer year/age first occurred | Medical conditions |
| 134 | Number of self-reported cancers | Medical conditions |
| 136 | Number of operations, self-reported | Operations |
| 137 | Number of treatments/medications taken | Medications |
| 40005 | Date of cancer diagnosis | Cancer register |
| 40006 | Type of cancer: ICD10 | Cancer register |
|  | Overall cancer (The definition of total cancer excludes in ICD-10 code D00-D48) ICD-10 code: C0, C1, C2, C3, C4, C5, C6, C70, C71, C72, C73, C74, C75, C76, C8, C9) |  |
| 40008 | Age at cancer diagnosis | Cancer register |
| 40000 | Date of death | Death register |
| 40001 | Underlying (primary) cause of death: ICD10 | Death register |
| 40002 | Contributory (secondary) causes of death: ICD10 | Death register |
| 40023 | Records in death dataset | Death register |

ICD: International Classification of Disease

**Table S3. 12 single-nucleotide polymorphisms list of evening chronotype identified in UK Biobank.**

| **SNP** | **EA** | **NEA** | **EA_freq** | **Beta** | **P-value** | **N** |
| --- | --- | --- | --- | --- | --- | --- |
| rs141175086 | C | T | 0.998 | 0.64933 | 1.65E-05 | 73,778 |
| rs2050122 | C | T | 0.804 | 0.03342 | 4.12E-04 | 73,778 |
| rs76681500 | G | A | 0.841 | 0.04139 | 3.07E-05 | 73,778 |
| rs10157197 | A | G | 0.398 | 0.04532 | 2.33E-10 | 73,778 |
| rs1144566 | C | T | 0.970 | 0.06446 | 3.58E-03 | 73,778 |
| rs11895698 | T | C | 0.143 | 0.02119 | 1.53E-02 | 73,778 |
| rs11708779 | G | A | 0.648 | 0.01703 | 2.01E-02 | 73,778 |
| rs148750727 | T | G | 0.995 | 0.27416 | 1.40E-05 | 73,778 |
| rs372229746 | A | G | 0.448 | 0.02531 | 3.24E-03 | 73,778 |
| rs17311976 | C | T | 0.193 | 0.03342 | 9.49E-05 | 73,778 |
| rs542675489 | C | CA | 0.596 | 0.01284 | 7.66E-02 | 73,778 |
| rs4821940 | C | T | 0.552 | 0.01284 | 1.02E-01 | 73,779 |

SNP, single-nucleotide polymorphisms; Chr, chromosome; EA, effect allele; NEA, non-effect allele; EA_freq, effect allele frequency.

**Table S4. 27 single-nucleotide polymorphisms list of short sleep duration identified in UK Biobank.**

| SNP | EA | NEA | EA_freq | Beta | *P*-value | *N* |
| --- | --- | --- | --- | --- | --- | --- |
| rs11763750 | G | A | 0.814346 | 0.035 | 5.10E-09 | 411,934 |
| rs1229762 | T | C | 0.664501 | 0.037 | 1.00E-12 | 411,934 |
| rs12518468 | C | T | 0.328456 | 0.031 | 8.50E-09 | 411,934 |
| rs12567114 | G | A | 0.7246 | 0.036 | 4.10E-09 | 411,934 |
| rs12661667 | T | C | 0.263495 | 0.028 | 2.80E-08 | 411,934 |
| rs12963463 | C | T | 0.299425 | 0.029 | 1.90E-11 | 411,934 |
| rs13107325 | T | C | 0.074528 | 0.075 | 2.50E-13 | 411,934 |
| rs1380703 | G | A | 0.383531 | 0.035 | 1.60E-11 | 411,934 |
| rs142180737 | C | T | 0.009491 | 0.154 | 4.40E-09 | 411,934 |
| rs1607227 | G | T | 0.704938 | 0.031 | 1.50E-09 | 411,934 |
| rs17005118 | A | G | 0.264936 | 0.03 | 2.50E-09 | 411,934 |
| rs17388803 | C | A | 0.105648 | 0.053 | 6.50E-10 | 411,934 |
| rs2014830 | C | T | 0.698128 | 0.03 | 2.70E-08 | 411,934 |
| rs205024 | C | T | 0.616724 | 0.031 | 2.70E-08 | 411,934 |
| rs2186122 | T | A | 0.561566 | 0.024 | 4.80E-09 | 411,934 |
| rs2820313 | G | A | 0.341112 | 0.031 | 2.30E-09 | 411,934 |
| rs2863957 | C | A | 0.781508 | 0.054 | 2.60E-18 | 411,934 |
| rs3776864 | A | C | 0.66721 | 0.031 | 1.70E-08 | 411,934 |
| rs4585442 | G | A | 0.311023 | 0.031 | 8.10E-10 | 411,934 |
| rs5757675 | G | T | 0.259528 | 0.034 | 2.70E-09 | 411,934 |
| rs59779556 | T | G | 0.553827 | 0.025 | 2.00E-08 | 411,934 |
| rs60882754 | A | T | 0.938985 | 0.055 | 1.80E-08 | 411,934 |
| rs7524118 | C | T | 0.708376 | 0.03 | 4.90E-08 | 411,934 |
| rs75539574 | A | C | 0.914664 | 0.045 | 8.40E-11 | 411,934 |
| rs7939345 | T | G | 0.207569 | 0.035 | 4.00E-08 | 411,934 |
| rs9321171 | C | T | 0.540122 | 0.031 | 4.20E-08 | 411,934 |
| rs9367621 | T | A | 0.43104 | 0.024 | 1.60E-08 | 411,934 |

SNP, single-nucleotide polymorphisms; SE, standard error; EA, effect allele; NEA, non-effect allele; EA_freq, effect allele frequency.

**Table S5. 8 single-nucleotide polymorphisms list of long sleep duration identified in UK Biobank.**

| **SNP** | **EA** | **NEA** | **EA_freq** | **Beta** | ***P*-value** | ***N*** |
| --- | --- | --- | --- | --- | --- | --- |
| rs10899257 | A | G | 0.144473 | 0.067659 | 4.60E-08 | 339,926 |
| rs17688916 | T | A | 0.796267 | 0.07139 | 1.10E-11 | 339,926 |
| rs17817288 | A | G | 0.518127 | 0.039221 | 8.90E-09 | 339,926 |
| rs3751046 | G | A | 0.147342 | 0.069526 | 2.00E-08 | 339,926 |
| rs549961083 | T | C | 0.001432 | 0.533565 | 9.60E-09 | 339,926 |
| rs6737318 | G | A | 0.221841 | 0.076035 | 3.40E-13 | 339,926 |
| rs7534398 | A | T | 0.201382 | 0.046884 | 2.10E-08 | 339,926 |
| rs75458655 | T | C | 0.022973 | 0.184818 | 5.40E-12 | 339,926 |

SNP, single-nucleotide polymorphisms; SE, standard error; EA, effect allele; NEA, non-effect allele; EA_freq, effect allele frequency.

**Table S6. 78 single-nucleotide polymorphisms list of continuous sleep duration identified in UK Biobank.**

| **SNP** | **EA** | **NEA** | **EA_freq** | **Beta** | ***P*-value** | ***N*** |
| --- | --- | --- | --- | --- | --- | --- |
| rs10173260 | C | T | 0.606235 | 0.77 | 2.90E-08 | 446,118 |
| rs10421649 | A | T | 0.55697 | 0.798 | 6.90E-09 | 446,118 |
| rs10483350 | G | A | 0.195418 | 1.042 | 1.50E-09 | 446,118 |
| rs1057703 | G | T | 0.146608 | 1.164 | 1.10E-09 | 446,118 |
| rs10761674 | C | T | 0.477334 | 0.74 | 4.20E-08 | 446,118 |
| rs10973207 | T | G | 0.157677 | 1.226 | 6.00E-11 | 446,118 |
| rs11190970 | G | A | 0.798661 | 0.923 | 4.60E-08 | 446,118 |
| rs112230981 | A | G | 0.94984 | 1.892 | 2.20E-09 | 446,118 |
| rs113113059 | T | C | 0.78 | 0.968 | 8.40E-09 | 446,118 |
| rs11567976 | T | C | 0.570908 | 0.768 | 2.10E-08 | 446,118 |
| rs11602180 | C | T | 0.836621 | 1.095 | 2.30E-09 | 446,118 |
| rs11614986 | A | G | 0.820952 | 0.983 | 2.70E-08 | 446,118 |
| rs11621908 | C | T | 0.917141 | 1.446 | 5.60E-09 | 446,118 |
| rs11643715 | G | C | 0.290942 | 0.834 | 3.20E-08 | 446,118 |
| rs11885663 | T | C | 0.247809 | 0.973 | 8.60E-10 | 446,118 |
| rs12246842 | A | G | 0.459815 | 0.804 | 3.90E-09 | 446,118 |
| rs12567114 | A | G | 0.275802 | 0.89 | 4.30E-09 | 446,118 |
| rs12607679 | T | C | 0.737717 | 1.208 | 8.30E-15 | 446,118 |
| rs12611523 | A | G | 0.545244 | 0.758 | 3.10E-08 | 446,118 |
| rs1263056 | A | G | 0.519099 | 0.768 | 2.00E-08 | 446,118 |
| rs12791153 | T | A | 0.081089 | 1.413 | 1.90E-08 | 446,118 |
| rs13088093 | G | T | 0.336317 | 0.976 | 7.00E-12 | 446,118 |
| rs13109404 | T | G | 0.928024 | 1.872 | 1.40E-12 | 446,118 |
| rs151014368 | A | G | 0.206258 | 0.966 | 9.10E-09 | 446,118 |
| rs1517572 | C | A | 0.580536 | 0.879 | 1.50E-10 | 446,118 |
| rs1553132 | G | A | 0.258433 | 0.87 | 2.50E-08 | 446,118 |
| rs17427571 | A | G | 0.684313 | 0.83 | 1.30E-08 | 446,118 |
| rs174560 | C | T | 0.314215 | 0.815 | 2.80E-08 | 446,118 |
| rs17732997 | C | G | 0.569098 | 0.776 | 1.20E-08 | 446,118 |
| rs1776776 | T | C | 0.873832 | 1.198 | 4.90E-09 | 446,118 |
| rs180769 | T | C | 0.424698 | 0.763 | 2.30E-08 | 446,118 |
| rs1939455 | G | T | 0.879446 | 1.226 | 1.20E-08 | 446,118 |
| rs1991556 | G | A | 0.773765 | 0.994 | 1.00E-09 | 446,118 |
| rs205024 | T | C | 0.383735 | 0.83 | 3.90E-09 | 446,118 |
| rs2072727 | T | C | 0.43617 | 0.795 | 7.90E-09 | 446,118 |
| rs2079070 | C | G | 0.264613 | 1.053 | 7.50E-12 | 446,118 |
| **SNP** | **EA** | **NEA** | **EA_freq** | **Beta** | ***P*-value** | ***N*** |
| rs2139261 | G | C | 0.748584 | 1.122 | 8.50E-11 | 446,118 |
| rs2192528 | A | G | 0.480065 | 0.802 | 2.70E-09 | 446,118 |
| rs2231265 | G | A | 0.772289 | 0.897 | 2.70E-08 | 446,118 |
| rs269054 | A | T | 0.422076 | 0.819 | 2.10E-09 | 446,118 |
| rs3095508 | C | A | 0.593529 | 0.921 | 3.10E-11 | 446,118 |
| rs330088 | C | T | 0.547012 | 0.868 | 2.70E-10 | 446,118 |
| rs34354917 | C | A | 0.710472 | 0.825 | 3.90E-08 | 446,118 |
| rs34556183 | A | G | 0.719606 | 1.015 | 2.30E-11 | 446,118 |
| rs34731055 | T | C | 0.18089 | 1.168 | 3.70E-11 | 446,118 |
| rs35531607 | C | T | 0.474083 | 0.77 | 1.50E-08 | 446,118 |
| rs365663 | A | G | 0.545963 | 0.878 | 1.00E-10 | 446,118 |
| rs374153 | C | T | 0.158085 | 1.057 | 9.10E-09 | 446,118 |
| rs4128364 | C | T | 0.339025 | 0.876 | 1.40E-09 | 446,118 |
| rs4538155 | T | C | 0.647426 | 0.779 | 3.60E-08 | 446,118 |
| rs4592416 | G | A | 0.464407 | 0.881 | 9.30E-11 | 446,118 |
| rs460692 | C | T | 0.137484 | 1.263 | 3.60E-10 | 446,118 |
| rs4767550 | G | A | 0.414138 | 0.858 | 6.30E-10 | 446,118 |
| rs55658675 | C | T | 0.644938 | 0.788 | 2.00E-08 | 446,118 |
| rs56372231 | T | C | 0.334093 | 1.017 | 2.20E-12 | 446,118 |
| rs61796569 | T | C | 0.269583 | 0.927 | 1.50E-09 | 446,118 |
| rs61985058 | T | C | 0.143176 | 1.116 | 1.30E-08 | 446,118 |
| rs62120041 | T | C | 0.933902 | 1.567 | 9.60E-09 | 446,118 |
| rs6575005 | T | C | 0.757854 | 0.934 | 4.40E-09 | 446,118 |
| rs7115226 | A | C | 0.073525 | 1.594 | 1.70E-09 | 446,118 |
| rs72804080 | G | A | 0.149928 | 1.068 | 2.90E-08 | 446,118 |
| rs73219758 | G | A | 0.708064 | 0.984 | 5.60E-11 | 446,118 |
| rs7503199 | C | T | 0.734267 | 0.885 | 1.00E-08 | 446,118 |
| rs75539574 | C | A | 0.085792 | 2.175 | 6.90E-19 | 446,118 |
| rs7556815 | A | G | 0.219144 | 2.443 | 1.30E-49 | 446,118 |
| rs7616632 | T | G | 0.522135 | 0.792 | 4.30E-09 | 446,118 |
| rs7644809 | T | C | 0.421606 | 0.784 | 1.60E-08 | 446,118 |
| rs7806045 | T | C | 0.754703 | 0.887 | 1.40E-08 | 446,118 |
| rs7915425 | T | C | 0.174682 | 1.144 | 2.00E-10 | 446,118 |
| rs7951019 | G | T | 0.032227 | 2.213 | 1.20E-08 | 446,118 |
| rs80193650 | G | A | 0.162466 | 1.01 | 4.10E-08 | 446,118 |
| rs8038326 | A | G | 0.72691 | 0.955 | 2.80E-10 | 446,118 |
| rs8050478 | G | A | 0.500253 | 0.96 | 1.70E-12 | 446,118 |
| **SNP** | **EA** | **NEA** | **EA_freq** | **Beta** | ***P*-value** | ***N*** |
| rs915416 | C | G | 0.289947 | 1.156 | 9.90E-15 | 446,118 |
| rs9345234 | C | A | 0.578016 | 0.781 | 1.80E-08 | 446,118 |
| rs9382445 | T | C | 0.62305 | 0.872 | 4.80E-10 | 446,118 |
| rs9903973 | C | T | 0.46702 | 0.766 | 2.60E-08 | 446,118 |
| rs9940646 | C | G | 0.577569 | 1.017 | 1.20E-13 | 446,118 |

SNP, single-nucleotide polymorphisms; SE, standard error; EA, effect allele; NEA, non-effect allele; EA_freq, effect allele frequency.

**Table S7. 57 single-nucleotide polymorphisms list of insomnia identified in UK Biobank.**

| **SNP** | **EA** | **NEA** | **EA_freq** | **Beta** | **p-value** | **N** |
| --- | --- | --- | --- | --- | --- | --- |
| rs11184946 | T | C | 0.417 | 0.01284 | 2.90E-10 | 237,627 |
| rs6664467 | G | A | 0.863 | 0.02119 | 4.50E-08 | 237,627 |
| rs4751 | T | G | 0.425 | 0.01284 | 1.60E-08 | 237,627 |
| rs2644128 | G | C | 0.550 | 0.01703 | 1.00E-12 | 237,627 |
| rs12405761 | A | C | 0.571 | 0.01703 | 2.60E-11 | 237,627 |
| rs2613503 | A | C | 0.802 | 0.01703 | 2.80E-07 | 237,627 |
| rs11804386 | A | G | 0.332 | 0.01703 | 2.60E-08 | 237,627 |
| rs62158170 | A | G | 0.784 | 0.02119 | 5.70E-13 | 237,627 |
| rs4577309 | A | G | 0.468 | 0.01284 | 3.70E-09 | 237,627 |
| rs12713372 | C | T | 0.566 | 0.00860 | 1.20E-05 | 237,627 |
| rs35881094 | G | T | 0.426 | 0.02119 | 3.00E-15 | 237,627 |
| rs2192338 | C | G | 0.780 | 0.02119 | 1.20E-07 | 237,627 |
| rs113851554 | T | G | 0.058 | 0.07918 | 1.30E-41 | 237,627 |
| rs72826719 | A | G | 0.049 | 0.04139 | 2.20E-12 | 237,627 |
| rs9845387 | C | A | 0.959 | 0.04139 | 2.10E-08 | 237,627 |
| rs6785034 | A | G | 0.422 | 0.01284 | 9.60E-05 | 237,627 |
| rs4683301 | T | A | 0.600 | 0.01703 | 4.60E-08 | 237,627 |
| rs10865954 | T | C | 0.334 | 0.01703 | 1.70E-09 | 237,627 |
| rs4688760 | T | C | 0.690 | 0.02119 | 1.00E-12 | 237,627 |
| rs55946513 | C | T | 0.934 | 0.02119 | 7.30E-06 | 237,627 |
| rs11097861 | G | A | 0.715 | 0.01703 | 1.70E-09 | 237,627 |
| rs1841625 | G | A | 0.432 | 0.01284 | 3.60E-08 | 237,627 |
| rs28061 | A | G | 0.692 | 0.01703 | 2.10E-08 | 237,627 |
| rs1592757 | C | G | 0.357 | 0.01703 | 4.60E-10 | 237,627 |
| rs7711696 | T | G | 0.305 | 0.02119 | 9.90E-12 | 237,627 |
| rs1430205 | T | C | 0.458 | 0.01284 | 3.60E-08 | 237,627 |
| rs6932158 | C | T | 0.491 | 0.01284 | 2.80E-08 | 237,627 |
| rs314280 | G | A | 0.548 | 0.01703 | 3.50E-10 | 237,627 |
| rs10947690 | G | A | 0.261 | 0.01703 | 3.50E-08 | 237,627 |
| rs3824081 | T | C | 0.475 | 0.01703 | 1.10E-08 | 237,627 |
| rs10280045 | G | C | 0.574 | 0.01703 | 1.00E-10 | 237,627 |
| rs302165 | G | A | 0.216 | 0.01703 | 2.40E-06 | 237,627 |
| rs6593005 | G | A | 0.741 | 0.01284 | 8.60E-09 | 237,627 |
| rs17151854 | T | G | 0.154 | 0.02531 | 2.40E-08 | 237,627 |
| rs11793831 | G | T | 0.583 | 0.01703 | 1.50E-06 | 237,627 |
| rs11793074 | A | G | 0.853 | 0.01703 | 2.30E-07 | 237,627 |
| rs10156602 | A | G | 0.638 | 0.01703 | 3.40E-12 | 237,627 |
| rs2296580 | G | T | 0.702 | 0.02119 | 8.70E-12 | 237,627 |
| rs11191595 | A | C | 0.937 | 0.04139 | 1.50E-09 | 237,627 |
| rs10838708 | G | A | 0.541 | 0.01284 | 2.50E-09 | 237,627 |
| rs68094047 | T | C | 0.250 | 0.01703 | 3.30E-09 | 237,627 |
| rs324017 | A | C | 0.295 | 0.02119 | 1.10E-10 | 237,627 |
| rs2956278 | G | A | 0.215 | 0.02119 | 1.30E-08 | 237,627 |
| rs2147141 | G | C | 0.543 | 0.01703 | 8.20E-08 | 237,627 |
| rs1923770 | T | A | 0.383 | 0.02119 | 2.30E-12 | 237,627 |
| **SNP** | **EA** | **NEA** | **EA_freq** | **Beta** | **p-value** | **N** |
| rs1031654 | C | A | 0.202 | 0.02119 | 2.00E-09 | 237,627 |
| rs4886140 | G | A | 0.668 | 0.01703 | 3.10E-10 | 237,627 |
| rs11635495 | C | T | 0.515 | 0.01703 | 6.80E-09 | 237,627 |
| rs4886860 | G | C | 0.234 | 0.02119 | 6.10E-12 | 237,627 |
| rs1544637 | T | C | 0.488 | 0.01284 | 3.00E-08 | 237,627 |
| rs3104778 | A | G | 0.589 | 0.01284 | 4.20E-08 | 237,627 |
| rs2062113 | T | C | 0.430 | 0.01703 | 1.90E-10 | 237,627 |
| rs17139246 | C | T | 0.389 | 0.01284 | 4.10E-08 | 237,627 |
| rs17669584 | G | A | 0.195 | 0.01703 | 3.60E-08 | 237,627 |
| rs11651809 | G | C | 0.296 | 0.02119 | 2.10E-15 | 237,627 |
| rs1942262 | A | G | 0.292 | 0.01703 | 1.10E-13 | 237,627 |
| rs11673344 | G | A | 0.380 | 0.01703 | 9.00E-10 | 237,627 |

SNP, single-nucleotide polymorphisms; SE, standard error; EA, effect allele; NEA, non-effect allele; EA_freq, effect allele frequency.

**Table S8. The flow chart of cancer patients for analyzing mortality.**


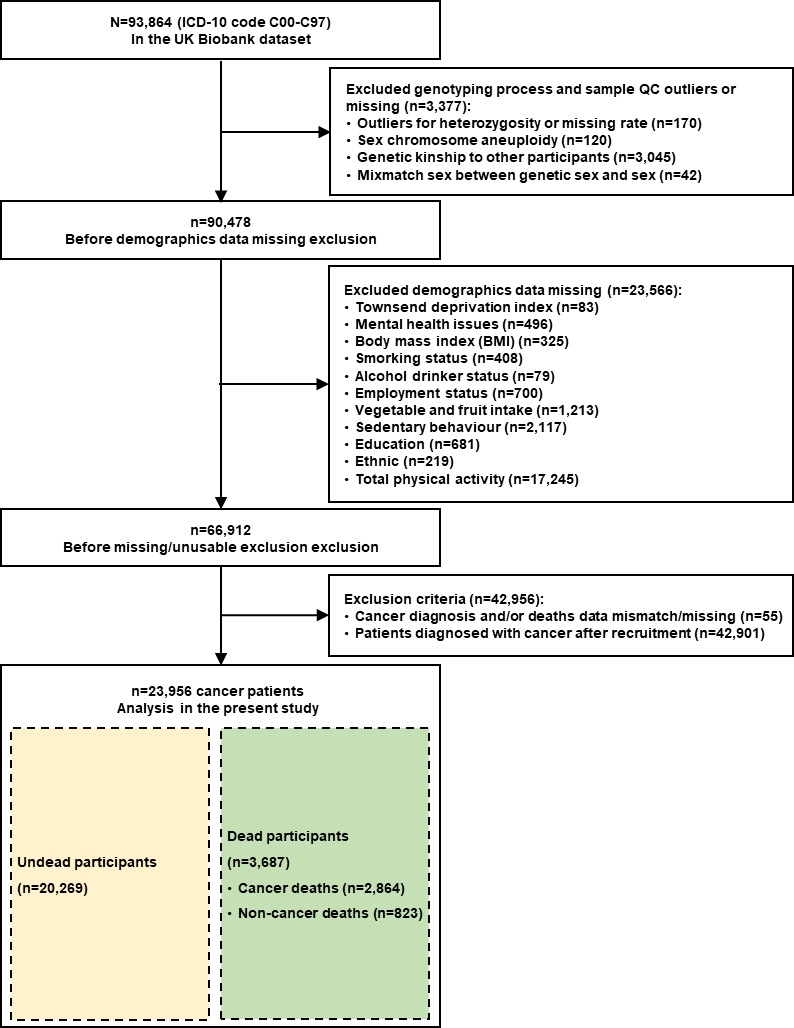


**Table S9. Multivariable Cox regression analysis for the pancancer incidence associated with sleep traits (n=326,417).**

|  | **Basic model^#^** | | **Further adjusted model*** | | **Full adjusted model^§^** | |
| --- | --- | --- | --- | --- | --- | --- |
| **Sleep traits** | **Hazard ratio (95%)** | **P value** | **Hazard ratio (95%)** | **P value** | **Hazard ratio (95%)** | **P value** |
| Chronotype |  |  |  |  |  |  |
| Definite morning | 1.00 (ref) | Ref | 1.00 (ref) | Ref | 1.00 (ref) | Ref |
| More morning | 0.99 (0.97-1.02) | 0.507 | 0.99 (0.97-1.02) | 0.443 | 0.99 (0.96-1.01) | 0.321 |
| More evening | 1.01 (0.98-1.03) | 0.713 | 0.99 (0.97-1.02) | 0.544 | 0.99 (0.96-1.02) | 0.396 |
| Definite evening | 1.06 (1.02-1.10) | 0.003 | 1.03 (0.99-1.07) | 0.121 | 1.03 (0.99-1.07) | 0.137 |
| Sleep duration |  |  |  |  |  |  |
| 7 hours | 1.00 (ref) | Ref | 1.00 (ref) | Ref | 1.00 (ref) | Ref |
| ≤5 hours | 0.96 (0.92-1.01) | 0.115 | 0.95 (0.91-0.99) | 0.030 | 0.94 (0.89-0.99) | 0.016 |
| 6 hours | 0.98 (0.95-1.01) | 0.131 | 0.97 (0.95-1.00) | 0.051 | 0.97 (0.94-1.00) | 0.038 |
| 8 hours | 1.03 (1.01-1.05) | 0.009 | 1.03 (1.01-1.05) | 0.015 | 1.03 (1.00-1.05) | 0.021 |
| ≥9 hours | 1.09 (1.03-1.16) | 0.003 | 1.04 (1.01-1.08) | 0.023 | 1.04 (1.00-1.08) | 0.031 |
| Insomnia symptom |  |  |  |  |  |  |
| Never | 1.00 (ref) | Ref | 1.00 (ref) | Ref | 1.00 (ref) | Ref |
| sometimes | 1.01 (0.99-1.04) | 0.322 | 1.01 (0.98-1.03) | 0.575 | 1.01 (0.99-1.04) | 0.400 |
| Usually | 1.03 (1.00-1.06) | 0.032 | 1.01 (0.99-1.04) | 0.307 | 1.03 (1.00-1.06) | 0.035 |

^#^ Basic model: Adjusted for age, sex, assessment center, top 10 genetic principal components and genotyping array.

* Further adjusted model: Adjusted for age, sex, assessment center, top 10 genetic principal components, genotyping array, body mass index, employment status, Townsend deprivation index, smoking status, drinking status and mental health issues, vegTable Sand fruit intake, sedentary behavior, comorbidity, total physical activity, education, ethnicity and family history.

^§^ Full adjusted model: Adjusted for age, sex, assessment center, top 10 genetic principal components, genotyping array, body mass index, employment status, Townsend deprivation index, smoking status, drinking status and mental health issues, vegTable Sand fruit intake, sedentary behavior, comorbidity, total physical activity, education, ethnicity, family history and other sleep traits. Statistical significance was defined as *P* < 0.05.

**Table S10. Multivariable Cox regression analysis for the all-cause mortality among cancer patients associated with sleep traits (n=23,956).**

|  | **Basic model^#^** | | **Further adjusted model*** | | **Full adjusted model^§^** | |
| --- | --- | --- | --- | --- | --- | --- |
| **Sleep traits** | **Hazard ratio (95%)** | **P value** | **Hazard ratio (95%)** | **P value** | **Hazard ratio (95%)** | **P value** |
| Chronotype |  |  |  |  |  |  |
| Definite morning | 1.00 (ref) | Ref | 1.00 (ref) | Ref | 1.00 (ref) | Ref |
| More morning | 0.88 (0.81-0.96) | 0.005 | 0.91 (0.83-0.99) | 0.029 | 0.91 (0.83-0.99) | 0.032 |
| More evening | 0.98 (0.89-1.01) | 0.620 | 0.95 (0.87-1.04) | 0.307 | 0.95 (0.87-1.04) | 0.302 |
| Definite evening | 1.08 (0.95-1.23) | 0.226 | 0.96 (0.84-1.09) | 0.545 | 0.95 (0.83-1.08) | 0.450 |
| Sleep duration |  |  |  |  |  |  |
| 7 hours | 1.00 (ref) | Ref | 1.00 (ref) | Ref | 1.00 (ref) | Ref |
| ≤5 hours | 1.38 (1.19-1.60) | <0.001 | 1.05 (0.90-1.21) | 0.558 | 1.02 (0.87-1.19) | 0.848 |
| 6 hours | 1.21 (1.10-1.33) | <0.001 | 1.12 (1.02-1.24) | 0.017 | 1.13 (1.02-1.25) | 0.019 |
| 8 hours | 1.04 (0.96-1.13) | 0.299 | 1.00 (0.92-1.08) | 0.230 | 1.02 (0.93-1.11) | 0.693 |
| ≥9 hours | 1.64 (1.48-1.82) | <0.001 | 1.29 (1.16-1.43) | <0.001 | 1.30 (1.16-1.45) | <0.001 |
| Insomnia symptom |  |  |  |  |  |  |
| Never | 1.00 (ref) | Ref | 1.00 (ref) | Ref | 1.00 (ref) | Ref |
| sometimes | 1.14 (1.04-1.24) | 0.004 | 1.06 (0.97-1.16) | 0.167 | 1.09 (0.99-1.20) | 0.064 |
| Usually | 1.41 (1.29-1.55) | <0.001 | 1.14 (1.04-1.25) | 0.005 | 1.17 (1.06-1.30) | 0.002 |

^#^ Basic model: Adjusted for age, sex, assessment center, top 10 genetic principal components and genotyping array.

^*^ Further adjusted model: Adjusted for age, sex, assessment center, top 10 genetic principal components, genotyping array, body mass index, employment status, Townsend deprivation index, smoking status, drinking status and mental health issues, vegTable Sand fruit intake, sedentary behavior, comorbidity, total physical activity, education, ethnicity, family history, number of self-reported cancers, operation and treatments.

^§^ Full adjusted model: Adjusted for age, sex, assessment center, top 10 genetic principal components, genotyping array, body mass index, employment status, Townsend deprivation index, smoking status, drinking status and mental health issues, vegTable Sand fruit intake, sedentary behavior, comorbidity, total physical activity, education, ethnicity, family history, number of self-reported cancers, operation, treatments and other sleep traits. Statistical significance was defined as *P* < 0.05.

**Table S11. Multivariable Cox regression analysis for the cancer mortality among cancer patients associated with sleep traits in the 5 years (n=4,962).**

|  | **Basic model^#^** | | **Further adjusted model*** | | **Full adjusted model^§^** | |
| --- | --- | --- | --- | --- | --- | --- |
| **Sleep traits** | **Hazard ratio (95%)** | **P value** | **Hazard ratio (95%)** | **P value** | **Hazard ratio (95%)** | **P value** |
| Chronotype |  |  |  |  |  |  |
| Definite morning | 1.00 (ref) | Ref | 1.00 (ref) | Ref | 1.00 (ref) | Ref |
| More morning | 0.93 (0.70-1.24) | 0.614 | 0.92 (0.69-1.23) | 0.573 | 0.95 (0.70-1.27) | 0.714 |
| More evening | 0.87 (0.63-1.20) | 0.392 | 0.85 (0.62-1.18) | 0.328 | 0.85 (0.61-1.18) | 0.338 |
| Definite evening | 1.06 (0.68-1.67) | 0.793 | 0.86 (0.54-1.38) | 0.541 | 0.89 (0.56-1.43) | 0.638 |
| Sleep duration |  |  |  |  |  |  |
| 7 hours | 1.00 (ref) | Ref | 1.00 (ref) | Ref | 1.00 (ref) | Ref |
| ≤5 hours | 1.71 (1.06-2.77) | 0.028 | 1.47 (0.90-2.41) | 0.127 | 1.45 (0.84-2.49) | 0.184 |
| 6 hours | 1.32 (0.95-1.83) | 0.102 | 1.38 (0.99-1.93) | 0.057 | 1.50 (1.06-2.22) | 0.022 |
| 8 hours | 1.10 (0.82-1.48) | 0.507 | 1.14 (0.85-1.54) | 0.382 | 1.13 (0.83-1.55) | 0.436 |
| ≥9 hours | 2.15 (1.52-3.03) | <0.001 | 1.92 (1.34-2.74) | <0.001 | 1.84 (1.25-2.70) | 0.002 |
| Insomnia |  |  |  |  |  |  |
| Never | 1.00 (ref) | Ref | 1.00 (ref) | Ref | 1.00 (ref) | Ref |
| sometimes | 1.27 (0.94-1.71) | 0.118 | 1.25 (0.92-1.68) | 0.152 | 1.28 (0.93-1.77) | 0.135 |
| Usually | 1.33 (0.97-1.84） | 0.079 | 1.11 (0.80-1.54） | 0.524 | 1.14 (0.80-1.63） | 0.475 |

^#^ Basic model: Adjusted for age, sex, assessment center, top 10 genetic principal components and genotyping array.

^*^ Further adjusted model: Adjusted for age, sex, assessment center, top 10 genetic principal components, genotyping array, body mass index, employment status, Townsend deprivation index, smoking status, drinking status and mental health issues, vegTable Sand fruit intake, sedentary behavior, comorbidity, total physical activity, education, ethnicity, family history, number of self-reported cancers, operation and treatments.

^§^ Full adjusted model: Adjusted for age, sex, assessment center, top 10 genetic principal components, genotyping array, body mass index, employment status, Townsend deprivation index, smoking status, drinking status and mental health issues, vegTable Sand fruit intake, sedentary behavior, comorbidity, total physical activity, education, ethnicity, family history, number of self-reported cancers, operation, treatments and other sleep traits.

Statistical significance was defined as *P* < 0.05.

**Table S12. Multivariable Cox regression analysis for the cancer mortality associated with sleep traits among cancer patients during the follow-up years (n=23,133).**

|  | **Basic model^#^** | | **Further adjusted model*** | | **Full adjusted model^§^** | |
| --- | --- | --- | --- | --- | --- | --- |
| **Sleep traits** | **Hazard ratio (95%)** | **P value** | **Hazard ratio (95%)** | **P value** | **Hazard ratio (95%)** | **P value** |
| Chronotype |  |  |  |  |  |  |
| Definite morning | 1.00 (ref) | Ref | 1.00 (ref) | Ref | 1.00 (ref) | Ref |
| More morning | 0.90 (0.82-0.99) | 0.032 | 0.91 (0.83-1.01) | 0.071 | 0.91 (0.83-1.01) | 0.073 |
| More evening | 0.94 (0.85-1.04) | 0.239 | 0.91 (0.82-1.01) | 0.090 | 0.91 (0.82-1.01) | 0.091 |
| Definite evening | 1.03 (0.89-1.20) | 0.653 | 0.92 (0.79-1.07) | 0.286 | 0.91 (0.79-1.06) | 0.241 |
| Sleep duration |  |  |  |  |  |  |
| 7 hours | 1.00 (ref) | Ref | 1.00 (ref) | Ref | 1.00 (ref) | Ref |
| ≤5 hours | 1.28 (1.08-1.52) | 0.004 | 1.03 (0.86-1.22) | 0.775 | 0.98 (0.81-1.18) | 0.826 |
| 6 hours | 1.21 (1.09-1.35) | <0.001 | 1.14 (1.02-1.27) | 0.019 | 1.13 (1.01-1.27) | 0.033 |
| 8 hours | 1.02 (0.93-1.12) | 0.672 | 0.98 (0.89-1.08) | 0.670 | 1.00 (0.90-1.10) | 0.991 |
| ≥9 hours | 1.63 (1.45-1.83) | <0.001 | 1.33 (1.18-1.50) | <0.001 | 1.35 (1.18-1.53) | <0.001 |
| Insomnia |  |  |  |  |  |  |
| Never | 1.00 (ref) | Ref | 1.00 (ref) | Ref | 1.00 (ref) | Ref |
| sometimes | 1.15 (1.04-1.27) | 0.006 | 1.08 (0.98-1.20) | 0.121 | 1.14 (1.02-1.27) | 0.020 |
| Usually | 1.43 (1.29-1.59) | <0.001 | 1.19 (1.07-1.32) | 0.002 | 1.25 (1.11-1.41) | <0.001 |

^#^ Basic model: Adjusted for age, sex, assessment center, top 10 genetic principal components and genotyping array.

^*^ Further adjusted model: Adjusted for age, sex, assessment center, top 10 genetic principal components, genotyping array, body mass index, employment status, Townsend deprivation index, smoking status, drinking status and mental health issues, vegTable Sand fruit intake, sedentary behavior, comorbidity, total physical activity, education, ethnicity, family history, number of self-reported cancers, operation and treatments.

^§^ Full adjusted model: Adjusted for age, sex, assessment center, top 10 genetic principal components, genotyping array, body mass index, employment status, Townsend deprivation index, smoking status, drinking status and mental health issues, vegTable Sand fruit intake, sedentary behavior, comorbidity, total physical activity, education, ethnicity, family history, number of self-reported cancers, operation, treatments and other sleep traits.

Statistical significance was defined as *P* < 0.05.

**Table S13. Associations between evening chronotype unweighted GRS and potential confounders among pancancer incidence samples in UK Biobank (N=296,119).**

| **Instrument** | **Confounder** | **Coefficients** | **SE** | ***P* value** |
| --- | --- | --- | --- | --- |
| Evening chronotype scores | Sex | 0.004 | 0.007 | 0.589 |
| Evening chronotype scores | Age | 8.78E-05 | 0.001 | 0.862 |
| Evening chronotype scores | Townsend deprivation index | 0.001 | 0.001 | 0.237 |
| Evening chronotype scores | Mental health issuess | 0.004 | 0.011 | 0.689 |
| Evening chronotype scores | Body mass index | -0.002 | 0.001 | **0.041** |
| Evening chronotype scores | Previous smoking | -0.007 | 0.007 | 0.371 |
| Evening chronotype scores | Current smoking | -0.029 | 0.012 | **0.011** |
| Evening chronotype scores | Previous alcohol drinker | 0.034 | 0.025 | 0.181 |
| Evening chronotype scores | Current alcohol drinker | -0.004 | 0.018 | 0.837 |
| Evening chronotype scores | Retired or not in the workforce | -4.89E-05 | 0.009 | 0.995 |
| Evening chronotype scores | Job involves shift work | 0.007 | 0.012 | 0.552 |
| Evening chronotype scores | Job involves night shift work | 0.124 | 0.286 | 0.664 |
| Evening chronotype scores | VegTable Sand fruit intake | -0.003 | 0.001 | **0.024** |
| Evening chronotype scores | Sedentary behavior | 0.001 | 0.001 | 0.345 |
| Evening chronotype scores | Comorbidity | 0.002 | 0.002 | 0.322 |
| Evening chronotype scores | Moderate physical activity | -0.003 | 0.009 | 0.712 |
| Evening chronotype scores | High physical activity | -0.001 | 0.009 | 0.929 |
| Evening chronotype scores | Education | -0.003 | 0.007 | 0.657 |
| Evening chronotype scores | Ethnicity | 0.099 | 0.044 | **0.024** |
| Evening chronotype scores | Family history | 0.005 | 0.007 | 0.474 |
| Evening chronotype scores | Short sleep duration | 0.016 | 0.009 | 0.078 |
| Evening chronotype scores | Long sleep duration | 0.011 | 0.014 | 0.400 |
| Evening chronotype scores | Sometimes insomnia symptom | 0.010 | 0.008 | 0.228 |
| Evening chronotype scores | Usually insomnia symptom | -0.012 | 0.010 | 0.230 |

Coefficients are in terms of an average-SNP increase in the allele score per unit/level increase in confounder. SE, standard error. *P*-value threshold of 0.05/24=2.08E-03 and *P*<0.05 as supporting evidence.

**Table S14. Associations between short sleep duration unweighted GRS and potential confounders among pancancer incidence samples in UK Biobank (N=296,119).**

| **Instrument** | **Confounder** | **Coefficients** | **SE** | ***P* value** |
| --- | --- | --- | --- | --- |
| Short sleep duration scores | Sex | 0.026 | 0.012 | **0.033** |
| Short sleep duration scores | Age | -0.003 | 0.001 | **0.002** |
| Short sleep duration scores | Townsend deprivation index | 0.000 | 0.002 | 0.957 |
| Short sleep duration scores | Mental health issuess | 0.029 | 0.019 | 0.117 |
| Short sleep duration scores | Body mass index | 0.003 | 0.001 | **0.011** |
| Short sleep duration scores | Previous smoking | 0.004 | 0.013 | 0.761 |
| Short sleep duration scores | Current smoking | 0.014 | 0.020 | 0.484 |
| Short sleep duration scores | Previous alcohol drinker | -0.020 | 0.045 | 0.655 |
| Short sleep duration scores | Current alcohol drinker | -0.059 | 0.032 | 0.066 |
| Short sleep duration scores | Retired or not in the workforce | 0.028 | 0.015 | 0.057 |
| Short sleep duration scores | Job involves shift work | -0.021 | 0.020 | 0.315 |
| Short sleep duration scores | Job involves night shift work | 0.167 | 0.501 | 0.740 |
| Short sleep duration scores | VegTable Sand fruit intake | -0.001 | 0.002 | 0.680 |
| Short sleep duration scores | Sedentary behavior | 0.009 | 0.003 | **3.01E-04** |
| Short sleep duration scores | Comorbidity | 0.012 | 0.004 | **4.51E-04** |
| Short sleep duration scores | Moderate physical activity | 0.019 | 0.015 | 0.209 |
| Short sleep duration scores | High physical activity | 0.019 | 0.015 | 0.218 |
| Short sleep duration scores | Education | -0.105 | 0.012 | **7.99E-18** |
| Short sleep duration scores | Ethnicity | -0.031 | 0.077 | 0.683 |
| Short sleep duration scores | Family history | 0.024 | 0.012 | 0.051 |
| Short sleep duration scores | Morning chronotype | -0.018 | 0.015 | 0.213 |
| Short sleep duration scores | Evening chronotype | 0.009 | 0.022 | 0.689 |
| Short sleep duration scores | Sometimes insomnia symptom | 0.058 | 0.014 | **5.27E-05** |
| Short sleep duration scores | Usually insomnia symptom | 0.091 | 0.017 | **7.90E-08** |

Coefficients are in terms of an average-SNP increase in the allele score per unit/level increase in confounder. SE, standard error. P-value threshold of 0.05/24=2.08E-03 and P<0.05 as supporting evidence.

**Table S15. Associations between long sleep duration unweighted GRS and potential confounders among pancancer incidence samples in UK Biobank (N=296,119).**

| **Instrument** | **Confounder** | **Coefficients** | **SE** | ***P* value** |
| --- | --- | --- | --- | --- |
| Long sleep duration scores | Sex | -0.006 | 0.006 | 0.326 |
| Long sleep duration scores | Age | -1.87E-04 | 4.12E-04 | 0.650 |
| Long sleep duration scores | Townsend deprivation index | -4.2E-05 | 0.001 | 0.967 |
| Long sleep duration scores | Mental health issuess | -0.014 | 0.009 | 0.097 |
| Long sleep duration scores | Body mass index | -0.008 | 0.001 | **1.44E-37** |
| Long sleep duration scores | Previous smoking | -0.005 | 0.006 | 0.384 |
| Long sleep duration scores | Current smoking | -0.009 | 0.009 | 0.359 |
| Long sleep duration scores | Previous alcohol drinker | 0.006 | 0.021 | 0.755 |
| Long sleep duration scores | Current alcohol drinker | -0.020 | 0.015 | 0.185 |
| Long sleep duration scores | Retired or not in the workforce | -0.011 | 0.007 | 0.126 |
| Long sleep duration scores | Job involves shift work | -0.008 | 0.009 | 0.373 |
| Long sleep duration scores | Job involves night shift work | 0.117 | 0.232 | 0.616 |
| Long sleep duration scores | VegTable Sand fruit intake | -0.002 | 0.001 | 0.099 |
| Long sleep duration scores | Sedentary behavior | 0.001 | 0.001 | 0.219 |
| Long sleep duration scores | Comorbidity | 4.84E-04 | 0.002 | 0.766 |
| Long sleep duration scores | Moderate physical activity | -0.008 | 0.007 | 0.236 |
| Long sleep duration scores | High physical activity | -0.005 | 0.007 | 0.510 |
| Long sleep duration scores | Education | 0.008 | 0.006 | 0.173 |
| Long sleep duration scores | Ethnicity | 0.023 | 0.036 | 0.523 |
| Long sleep duration scores | Family history | 0.006 | 0.006 | 0.261 |
| Long sleep duration scores | Morning chronotype | 0.007 | 0.007 | 0.334 |
| Long sleep duration scores | Evening chronotype | 0.042 | 0.010 | **4.97E-05** |
| Long sleep duration scores | Sometimes insomnia symptom | -0.012 | 0.007 | 0.062 |
| Long sleep duration scores | Usually insomnia symptom | 0.010 | 0.008 | 0.190 |

Coefficients are in terms of an average-SNP increase in the allele score per unit/level increase in confounder. SE, standard error. P-value threshold of 0.05/24=2.08E-03 and P<0.05 as supporting evidence.

**Table S16. Associations between insomnia unweighted GRS and potential confounders among pancancer incidence samples in UK Biobank (N=296,119).**

| **Instrument** | **Confounder** | **Coefficients** | **SE** | **P value** |
| --- | --- | --- | --- | --- |
| Insomnia symptom scores | Sex | 0.080 | 0.019 | **2.07E-05** |
| Insomnia symptom scores | Age | -0.007 | 0.001 | **4.43E-07** |
| Insomnia symptom scores | Townsend deprivation index | 0.001 | 0.003 | 0.732 |
| Insomnia symptom scores | Mental health issuess | 0.046 | 0.029 | 0.109 |
| Insomnia symptom scores | Body mass index | 0.002 | 0.002 | 0.284 |
| Insomnia symptom scores | Previous smoking | 0.051 | 0.020 | **0.010** |
| Insomnia symptom scores | Current smoking | 0.065 | 0.031 | **0.038** |
| Insomnia symptom scores | Previous alcohol drinker | 0.223 | 0.069 | **0.001** |
| Insomnia symptom scores | Current alcohol drinker | 0.078 | 0.049 | 0.113 |
| Insomnia symptom scores | Retired or not in the workforce | -0.018 | 0.023 | 0.432 |
| Insomnia symptom scores | Job involves shift work | -0.036 | 0.032 | 0.261 |
| Insomnia symptom scores | Job involves night shift work | -0.585 | 0.775 | 0.450 |
| Insomnia symptom scores | VegTable Sand fruit intake | -0.008 | 0.003 | **0.013** |
| Insomnia symptom scores | Sedentary behavior | 0.001 | 0.004 | 0.822 |
| Insomnia symptom scores | Comorbidity | 0.026 | 0.005 | **2.11E-06** |
| Insomnia symptom scores | Moderate physical activity | 0.068 | 0.024 | **0.004** |
| Insomnia symptom scores | High physical activity | 0.095 | 0.024 | **7.11E-05** |
| Insomnia symptom scores | Education | -0.062 | 0.019 | **0.001** |
| Insomnia symptom scores | Ethnicity | 0.087 | 0.119 | 0.463 |
| Insomnia symptom scores | Family history | -0.031 | 0.019 | 0.098 |
| Insomnia symptom scores | Morning chronotype | 0.039 | 0.023 | 0.090 |
| Insomnia symptom scores | Evening chronotype | -0.046 | 0.035 | 0.180 |
| Insomnia symptom scores | Short sleep duration | 0.224 | 0.045 | **4.78E-07** |
| Insomnia symptom scores | Long sleep duration | -0.033 | 0.037 | 0.372 |

Coefficients are in terms of an average-SNP increase in the allele score per unit/level increase in confounder. SE, standard error. P-value threshold of 0.05/24=2.08E-03 and P<0.05 as supporting evidence.

**Table S17. Mendelian randomization estimates for genetically predicted sleep traits with adjustment for other confounders (pancancer incidence).**

| **Instrument** | **Outcomes** | **N** | **Basic adjustment*** | | | **Full adjustment^#^** | | |
| --- | --- | --- | --- | --- | --- | --- | --- | --- |
|  |  |  | **OR** | **95% CI** | ***P* value** | **OR** | **95% CI** | ***P* value** |
| Evening chronotype | Pancancer incidence | 296,119 | 1.22 | (1.03,1.45) | **0.023*** | 1.09 | (0.92, 1.30) | 0.306 |
| Short sleep duration | Pancancer incidence | 296,119 | 0.87 | (0.82,0.93) | **7.18E-05**** | 0.87 | (0.81, 0.93) | **1.84E-05**** |
| Long sleep duration | Pancancer incidence | 296,119 | 1.62 | (1.28,2.03) | **4.13E-05**** | 1.64 | (1.31,2.06) | **2.11E-05**** |
| Insomnia symptom | Pancancer incidence | 296,119 | 1.03 | (1.00, 1.06) | 0.069 | 1.04 | (1.00, 1.08) | 0.050 |

* Adjusted for age, sex, assessment centers, top 10 genetic principal components and genotyping array. ^#^ Full adjusted for the GRS association factors. Odds ratios are per category increase in chronotype (from definite morning, intermediate morning, intermediate evening, and definite evening), per category increase in sleep duration (from ≤5 hours, 6 hours, 7 hours, 8 hours, and ≥9 hours), and per category increase in insomnia risk (from no, sometimes, and frequent insomnia symptoms). * p<0.05 and ** p< bonferroni-corrected threshold of 0.05/groups.

**Table S18. Mendelian randomization estimates for genetically predicted sleep traits with adjustment for other confounders after excluding the disturbed participants (pancancer incidence).**

| **Instrument** | **Outcomes** | **N** | **Basic adjustment*** | | | **Full adjustment ^#^** | | |
| --- | --- | --- | --- | --- | --- | --- | --- | --- |
|  |  |  | **OR** | **95% CI** | ***P* value** | **OR** | **95% CI** | ***P* value** |
| Short sleep duration (Participants with insomnia symptoms were excluded)  Pancancer incidence  0.82  (0.61,1.10)  0.18  0.79  (0.59, 1.07)  0.13 | | | | | | | | |
|  | Pancancer incidence | 76,309 | 0.87 | (0.75-1.01) | 0.071 | 0.86 | (0.74, 1.00) | 0.053 |
| Long sleep duration (Participants with evening chronotype were excluded)  Pancancer incidence  1.59  (1.19, 2.12)  **1.52E-03****  1.50  (1.14,1.99)  **3.97E-03**** | | | | | | | | |
|  | Pancancer incidence | 185,552 | 1.43 | (1.09-1.87) | **9.56E-03**** | 1.41 | (1.08, 1.84) | **0.012*** |
| Insomnia symptom (Participants with short sleep duration were excluded) | | | | | | | | |
|  | Pancancer incidence | 225,756 | 1.04 | (1.00-1.08) | **0.040*** | 1.02 | (0.98,1.07) | 0.242 |

* Adjusted for age, sex, assessment centers, top 10 genetic principal components and genotyping array. ^#^ Further adjusted for the GRS association factors. ^§^ Additionally adjusted for other sleep traits. Odds ratios are per category increase in sleep duration (from ≤5 hours, 6 hours, 7 hours, 8 hours, and ≥9 hours), and per category increase in insomnia risk (from no, sometimes, and frequent insomnia symptoms). * p<0.05 and ** p< bonferroni-corrected threshold of 0.05/groups.

**Table S19. Associations between evening chronotype unweighted GRS and potential confounders among cancer samples in UK Biobank (N=21,819).**

| **Instrument** | **Confounder** | **Coefficients** | **SE** | **P value** |
| --- | --- | --- | --- | --- |
| Evening chronotype scores | Sex | 0.072 | 0.027 | **0.007** |
| Evening chronotype scores | Age | -0.001 | 0.001 | 0.544 |
| Evening chronotype scores | Townsend deprivation index | 0.000 | 0.005 | 0.949 |
| Evening chronotype scores | Mental health issuess | 0.019 | 0.039 | 0.629 |
| Evening chronotype scores | Body mass index | -0.004 | 0.003 | 0.180 |
| Evening chronotype scores | Previous smoking | -0.007 | 0.026 | 0.785 |
| Evening chronotype scores | Current smoking | -0.030 | 0.046 | 0.514 |
| Evening chronotype scores | Previous alcohol drinker | -0.026 | 0.090 | 0.776 |
| Evening chronotype scores | Current alcohol drinker | -0.084 | 0.067 | 0.208 |
| Evening chronotype scores | Retired or not in the workforce | -0.016 | 0.029 | 0.587 |
| Evening chronotype scores | Job involves shift work | -0.049 | 0.070 | 0.488 |
| Evening chronotype scores | Job involves night shift work | -0.039 | 0.080 | 0.625 |
| Evening chronotype scores | VegTable Sand fruit intake | 0.005 | 0.004 | 0.195 |
| Evening chronotype scores | Sedentary behavior | -0.004 | 0.006 | 0.497 |
| Evening chronotype scores | Comorbidity | 0.000 | 0.008 | 0.974 |
| Evening chronotype scores | Moderate physical activity | 0.011 | 0.032 | 0.743 |
| Evening chronotype scores | High physical activity | 0.050 | 0.032 | 0.120 |
| Evening chronotype scores | Education | 0.017 | 0.026 | 0.502 |
| Evening chronotype scores | Ethnicity | 0.006 | 0.209 | 0.978 |
| Evening chronotype scores | Family history | -0.046 | 0.026 | 0.081 |
| Evening chronotype scores | Cancers | -0.001 | 0.024 | 0.975 |
| Evening chronotype scores | Operation | -0.065 | 0.051 | 0.199 |
| Evening chronotype scores | Treatments | 0.005 | 0.005 | 0.325 |
| Evening chronotype scores | Short sleep duration | 0.023 | 0.060 | 0.698 |
| Evening chronotype scores | Long sleep duration | -0.022 | 0.044 | 0.616 |
| Evening chronotype scores | Sometimes insomnia symptom | -0.019 | 0.032 | 0.544 |
| Evening chronotype scores | Usually insomnia symptom | -0.027 | 0.036 | 0.450 |

Coefficients are in terms of an average-SNP increase in the allele score per unit/level increase in confounder. SE, standard error. P-value threshold of 0.05/27=1.85E-03 and P<0.05 as supporting evidence.

**Table S20. Associations between short sleep duration unweighted GRS and potential confounders among cancer samples in UK Biobank (N=21,819).**

| **Instrument** | **Confounder** | **Coefficients** | **SE** | **P value** |
| --- | --- | --- | --- | --- |
| Short sleep duration scores | Sex | 0.057 | 0.047 | 0.224 |
| Short sleep duration scores | Age | -0.004 | 0.003 | 0.156 |
| Short sleep duration scores | Townsend deprivation index | 0.008 | 0.008 | 0.360 |
| Short sleep duration scores | Mental health issuess | 0.213 | 0.069 | **0.002** |
| Short sleep duration scores | Body mass index | 0.007 | 0.005 | 0.185 |
| Short sleep duration scores | Previous smoking | 0.017 | 0.047 | 0.714 |
| Short sleep duration scores | Current smoking | 0.055 | 0.082 | 0.503 |
| Short sleep duration scores | Previous alcohol drinker | -0.154 | 0.159 | 0.334 |
| Short sleep duration scores | Current alcohol drinker | -0.103 | 0.118 | 0.384 |
| Short sleep duration scores | Retired or not in the workforce | -0.001 | 0.051 | 0.988 |
| Short sleep duration scores | Job involves shift work | 0.071 | 0.124 | 0.567 |
| Short sleep duration scores | Job involves night shift work | -0.184 | 0.142 | 0.195 |
| Short sleep duration scores | VegTable Sand fruit intake | 0.003 | 0.007 | 0.694 |
| Short sleep duration scores | Sedentary behavior | -0.004 | 0.010 | 0.687 |
| Short sleep duration scores | Comorbidity | 0.001 | 0.014 | 0.919 |
| Short sleep duration scores | Moderate physical activity | -0.009 | 0.057 | 0.869 |
| Short sleep duration scores | High physical activity | 0.010 | 0.057 | 0.863 |
| Short sleep duration scores | Education | -0.099 | 0.046 | **0.032** |
| Short sleep duration scores | Ethnicity | 0.184 | 0.369 | 0.619 |
| Short sleep duration scores | Family history | 0.085 | 0.046 | 0.067 |
| Short sleep duration scores | Cancers | 0.104 | 0.043 | **0.016** |
| Short sleep duration scores | Operation | -0.066 | 0.090 | 0.463 |
| Short sleep duration scores | Treatments | -0.001 | 0.010 | 0.917 |
| Short sleep duration scores | Morning chronotype | -0.029 | 0.055 | 0.598 |
| Short sleep duration scores | Evening chronotype | -0.130 | 0.086 | 0.130 |
| Short sleep duration scores | Sometimes insomnia symptom | 0.103 | 0.057 | 0.068 |
| Short sleep duration scores | Usually insomnia symptom | 0.104 | 0.064 | 0.105 |

Coefficients are in terms of an average-SNP increase in the allele score per unit/level increase in confounder. SE, standard error. P-value threshold of 0.05/27=1.85E-03 and P<0.05 as supporting evidence.

**Table S21. Associations between long sleep duration unweighted GRS and potential confounders among cancer samples in UK Biobank (N=21,819).**

| **Instrument** | **Confounder** | **Coefficients** | **SE** | **P value** |
| --- | --- | --- | --- | --- |
| Long sleep duration scores | Sex | -0.022 | 0.021 | 0.303 |
| Long sleep duration scores | Age | -0.002 | 0.001 | 0.058 |
| Long sleep duration scores | Townsend deprivation index | 0.002 | 0.004 | 0.681 |
| Long sleep duration scores | Mental health issuess | -0.019 | 0.031 | 0.550 |
| Long sleep duration scores | Body mass index | -0.010 | 0.002 | **2.29E-05** |
| Long sleep duration scores | Previous smoking | 0.034 | 0.021 | 0.104 |
| Long sleep duration scores | Current smoking | -0.023 | 0.037 | 0.528 |
| Long sleep duration scores | Previous alcohol drinker | -0.089 | 0.073 | 0.219 |
| Long sleep duration scores | Current alcohol drinker | -0.045 | 0.054 | 0.403 |
| Long sleep duration scores | Retired or not in the workforce | 0.027 | 0.023 | 0.248 |
| Long sleep duration scores | Job involves shift work | -0.016 | 0.057 | 0.777 |
| Long sleep duration scores | Job involves night shift work | 0.001 | 0.065 | 0.993 |
| Long sleep duration scores | VegTable Sand fruit intake | 0.001 | 0.003 | 0.710 |
| Long sleep duration scores | Sedentary behavior | 0.009 | 0.005 | 0.063 |
| Long sleep duration scores | Comorbidity | -0.007 | 0.007 | 0.297 |
| Long sleep duration scores | Moderate physical activity | 0.021 | 0.026 | 0.426 |
| Long sleep duration scores | High physical activity | -0.008 | 0.026 | 0.768 |
| Long sleep duration scores | Education | -0.025 | 0.021 | 0.239 |
| Long sleep duration scores | Ethnicity | -0.018 | 0.168 | 0.913 |
| Long sleep duration scores | Family history | -0.036 | 0.021 | 0.085 |
| Long sleep duration scores | Cancers | 0.008 | 0.020 | 0.677 |
| Long sleep duration scores | Operation | 0.028 | 0.041 | 0.488 |
| Long sleep duration scores | Treatments | 0.005 | 0.004 | 0.288 |
| Long sleep duration scores | Morning chronotype | 0.010 | 0.025 | 0.696 |
| Long sleep duration scores | Evening chronotype | 0.121 | 0.039 | **0.002** |
| Long sleep duration scores | Sometimes insomnia symptom | 0.016 | 0.026 | 0.540 |
| Long sleep duration scores | Usually insomnia symptom | 0.024 | 0.029 | 0.420 |

Coefficients are in terms of an average-SNP increase in the allele score per unit/level increase in confounder. SE, standard error. P-value threshold of 0.05/27=1.85E-03 and P<0.05 as supporting evidence.

**Table S22. Associations between long sleep duration unweighted GRS and potential confounders among cancer samples in UK Biobank (N=21,819).**

| **Instrument** | **Confounder** | **Coefficients** | **SE** | **P value** |
| --- | --- | --- | --- | --- |
| Insomnia symptom scores | Sex | 0.207 | 0.072 | **0.004** |
| Insomnia symptom scores | Age | 0.000 | 0.004 | 0.970 |
| Insomnia symptom scores | Townsend deprivation index | -0.020 | 0.013 | 0.116 |
| Insomnia symptom scores | Mental health issuess | 0.294 | 0.106 | **0.005** |
| Insomnia symptom scores | Body mass index | 0.008 | 0.008 | 0.281 |
| Insomnia symptom scores | Previous smoking | -0.123 | 0.072 | 0.087 |
| Insomnia symptom scores | Current smoking | -0.006 | 0.126 | 0.962 |
| Insomnia symptom scores | Previous alcohol drinker | 0.215 | 0.246 | 0.382 |
| Insomnia symptom scores | Current alcohol drinker | 0.080 | 0.182 | 0.662 |
| Insomnia symptom scores | Retired or not in the workforce | -0.030 | 0.078 | 0.698 |
| Insomnia symptom scores | Job involves shift work | 0.229 | 0.191 | 0.232 |
| Insomnia symptom scores | Job involves night shift work | 0.161 | 0.218 | 0.460 |
| Insomnia symptom scores | VegTable Sand fruit intake | -0.033 | 0.011 | **0.004** |
| Insomnia symptom scores | Sedentary behaviour | 0.010 | 0.016 | 0.525 |
| Insomnia symptom scores | Comorbidity | 0.039 | 0.022 | 0.074 |
| Insomnia symptom scores | Moderate physical activity | 0.130 | 0.088 | 0.136 |
| Insomnia symptom scores | High physical activity | 0.190 | 0.088 | **0.030** |
| Insomnia symptom scores | Education | -0.091 | 0.071 | 0.200 |
| Insomnia symptom scores | Ethnicity | 0.879 | 0.569 | 0.122 |
| Insomnia symptom scores | Family history | -0.135 | 0.071 | 0.057 |
| Insomnia symptom scores | Cancers | 0.028 | 0.067 | 0.671 |
| Insomnia symptom scores | Operation | 0.002 | 0.138 | 0.990 |
| Insomnia symptom scores | Treatments | 0.009 | 0.015 | 0.524 |
| Insomnia symptom scores | Morning chronotype | 0.129 | 0.084 | 0.126 |
| Insomnia symptom scores | Evening chronotype | -0.181 | 0.133 | 0.173 |
| Insomnia symptom scores | Short sleep duration | -0.200 | 0.164 | 0.222 |
| Insomnia symptom scores | Long sleep duration | -0.199 | 0.121 | 0.100 |

Coefficients are in terms of an average-SNP increase in the allele score per unit/level increase in confounder. SE, standard error. P-value threshold of 0.05/27=1.85E-03 and P<0.05 as supporting evidence.

**Table S23. Mendelian randomization estimates for genetically predicted evening chronotype with adjustment for other confounders (mortality).**

| **Instrument** | **Outcomes** | **N** | **Basic adjustment*** | | | **Full adjustment ^#^** | | |
| --- | --- | --- | --- | --- | --- | --- | --- | --- |
|  |  |  | **OR** | **95% CI** | ***P* value** | **OR** | **95% CI** | ***P* value** |
| Evening chronotype | |  |  |  |  |  |  |  |
|  | All-cause mortality of cancer patients | 21,819 | 1.39 | (0.78, 2.49) | 0.262 | 1.26 | (0.71, 2.24) | 0.423 |
|  | 5-year cancer mortality  mortality | 4,513 | 0.63 | (0.07, 5.51) | 0.680 | 0.56 | (0.07, 4.78) | 0.595 |
|  | Cancer mortality | 21,065 | 0.92 | (0.48, 1.76) | 0.795 | 0.85 | (0.45, 1.61) | 0.610 |

* Adjusted for age, sex, assessment centers, top 10 genetic principal components and genotyping array. ^#^ Full adjusted for the GRS association factors. Odds ratios are per category increase in chronotype (from definite evening, intermediate evening, intermediate morning, and definite morning). * p<0.05 and ** p< bonferroni-corrected threshold of 0.05/groups.

**Table S24. Mendelian randomization estimates for genetically predicted short sleep duration and long sleep duration with adjustment for other confounders (mortality).**

| **Instrument** | **Outcomes** | **N** | **Basic adjustment*** | | | **Full adjustment ^#^** | | |
| --- | --- | --- | --- | --- | --- | --- | --- | --- |
|  |  |  | **OR** | **95% CI** | ***P* value** | **OR** | **95% CI** | ***P* value** |
| Short sleep duration | |  |  |  |  |  |  |  |
|  | All-cause mortality of cancer patients | 21,819 | 0.88 | (0.75,1.03) | 0.108 | 0.85 | (0.73, 1.00) | 0.050 |
|  | 5-year cancer mortality  mortality | 4,513 | 0.86 | (0.58,1.28) | 0.456 | 0.85 | (0.57, 1.26) | 0.419 |
|  | Cancer mortality | 21,065 | 0.88 | (0.74, 1.05) | 0.147 | 0.86 | (0.73, 1.02) | 0.088 |
| Long sleep duration | |  |  |  |  |  |  |  |
|  | All-cause mortality of cancer patients | 21,819 | 5.46 | (3.05, 9.79) | **1.18E-08**** | 5.56 | (3.15, 9.82) | **3.42E-09**** |
|  | 5-year cancer mortality  mortality | 4,513 | 5.97 | (0.23, 156) | 0.284 | 11.11 | (0.38, 321) | 0.161 |
|  | Cancer mortality | 21,065 | 4.83 | (2.60, 8.99) | **6.56E-07**** | 4.89 | (2.66,8.98) | **3.08E-07**** |

* Adjusted for age, sex, assessment centers, top 10 genetic principal components and genotyping array. ^#^ Full adjusted for the GRS association factors. Odds ratios are per category increase in sleep duration (from ≤5 hours, 6 hours, 7 hours, 8 hours, and ≥9 hours). * p<0.05 and ** p< bonferroni-corrected threshold of 0.05/groups.

**Table S25. Mendelian randomization estimates for genetically predicted insomnia symptom with adjustment for other sleep traits confounders (mortality).**

| **Instrument** | **Outcomes** | **N** | **Basic adjustment*** | | | **Further adjustment ^#^** | | |
| --- | --- | --- | --- | --- | --- | --- | --- | --- |
|  |  |  | **OR** | **95% CI** | ***P* value** | **OR** | **95% CI** | ***P* value** |
| Insomnia symptom | |  |  |  |  |  |  |  |
|  | All-cause mortality of cancer patients | 21,819 | 1.44 | (1.30, 1.59) | **7.65E-13**** | 1.41 | (1.27, 1.56) | **4.96E-11**** |
|  | 5-year cancer mortality  mortality | 4,513 | 1.35 | (1.00, 1.83) | 0.050 | 1.31 | (0.96, 1.77) | 0.085 |
|  | Cancer mortality | 21,065 | 1.51 | (1.34, 1.70) | **3.85E-12**** | 1.48 | (1.32, 1.67) | **7.29E-11**** |

* Adjusted for age, sex, assessment centers, top 10 genetic principal components and genotyping array. ^#^ Full adjusted for the GRS association factors. Odds ratios are per category increase in insomnia risk (from no, sometimes, and frequent insomnia symptoms). * p<0.05 and ** p< bonferroni-corrected threshold of 0.05/groups.

**Table S26. Non-linear Mendelian randomization results between genetically predicted continuous sleep durations and pancancer incidence and mortality among cancer patients using piecewise linear method.**


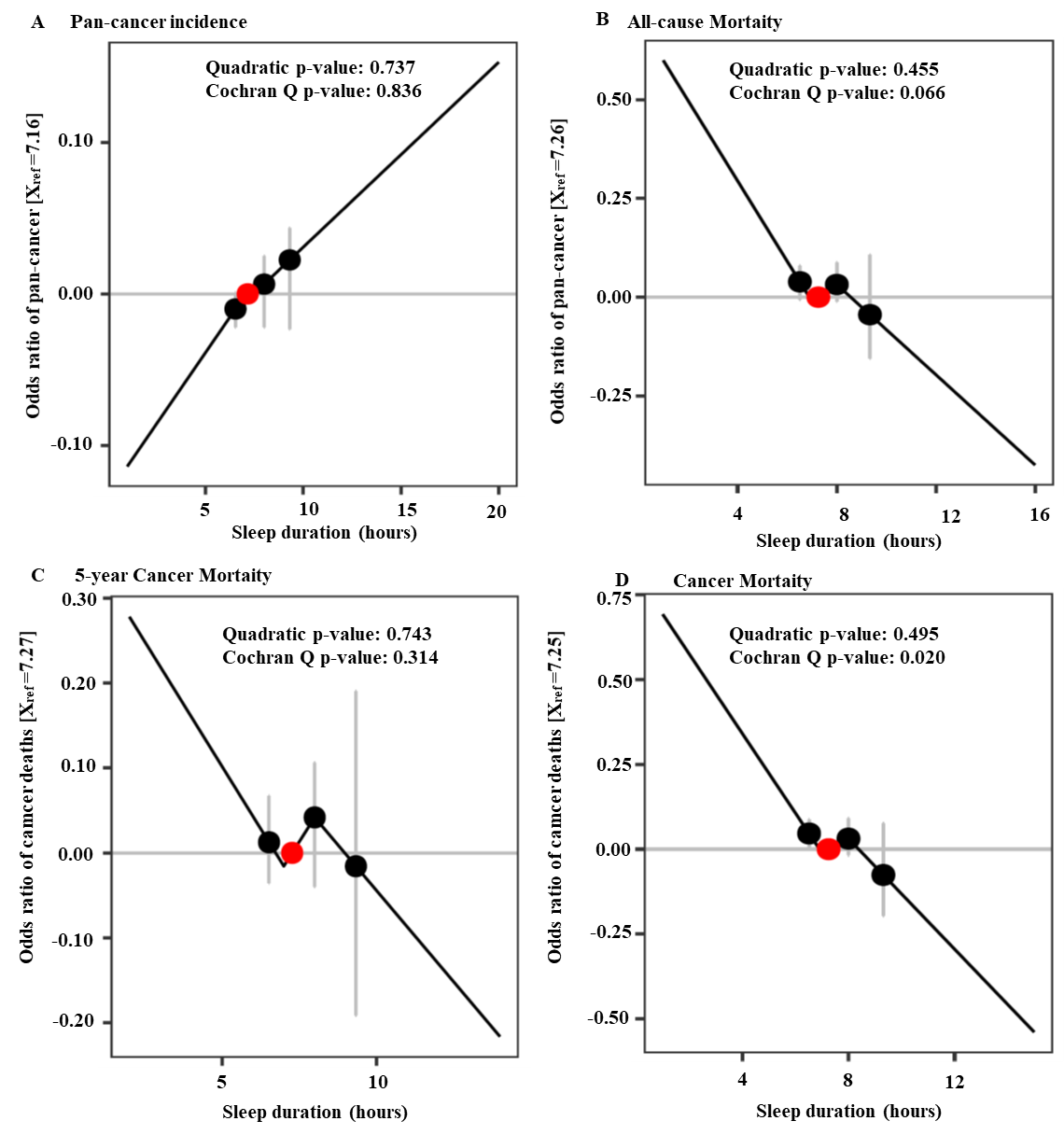


Localized average causal effects (LACE) for Pancancer incidence/mortality were estimated with piecewise linear method. Black dots (black vertical lines) mean the LACE (95% confidence interval) in each stratum; read dots represent reference point. P quadratic/Cochran Q = P-value for non-linearity from quadratic/Cochran Q test. A, Pancancer incidence; B, All-cause mortality of cancer patients; C, 5-year cancer mortality; D, Cancer mortality.

**Table S27. Non-linear Mendelian randomization results between genetically predicted continuous sleep durations and pancancer incidence and mortality among cancer patients using piecewise linear method (participants with extremely sleep duration were excluded).**

**
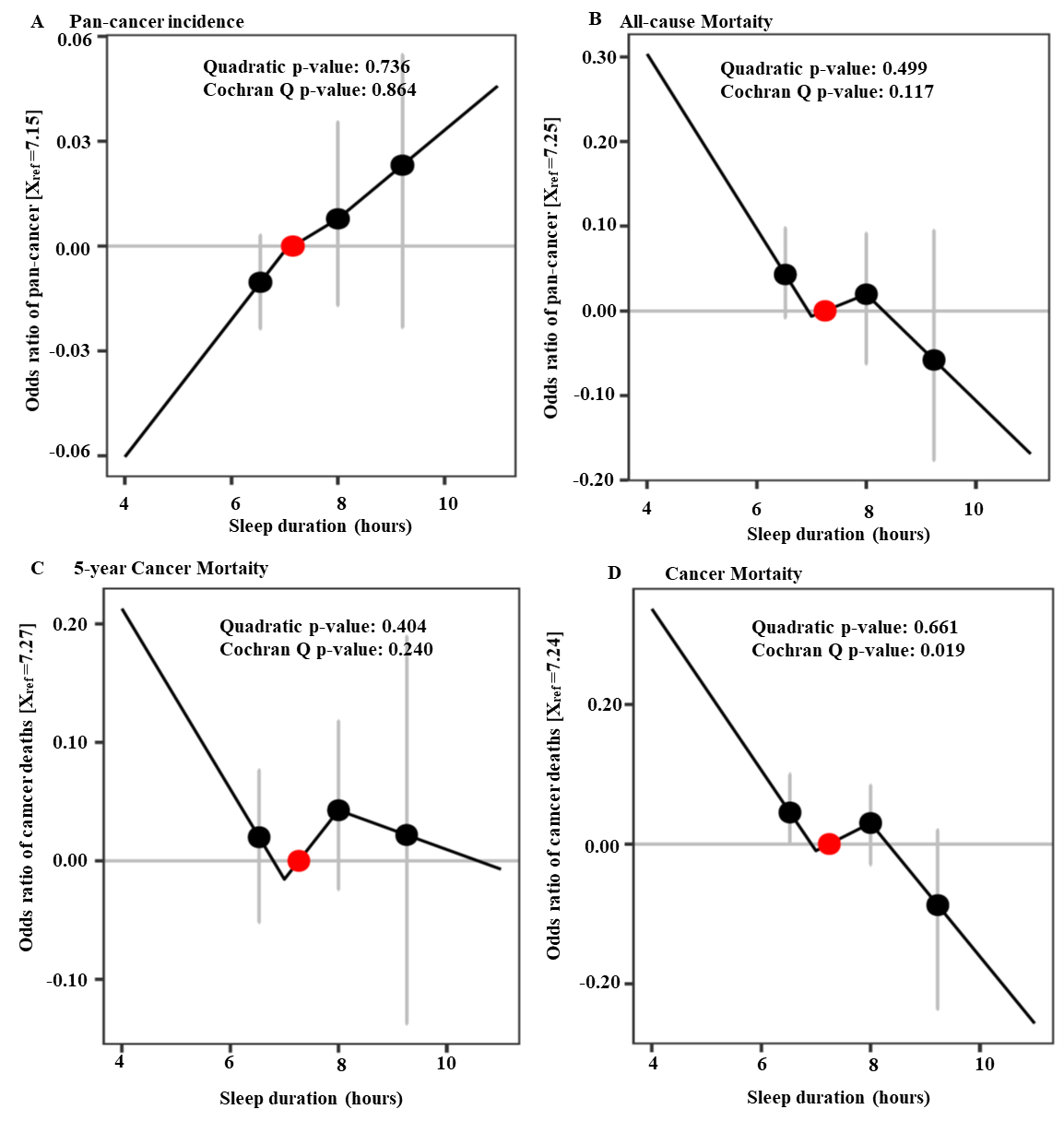
**

Localized average causal effects (LACE) for Pancancer incidence/mortality were estimated with piecewise linear method. Black dots (black vertical lines) mean the LACE (95% confidence interval) in each stratum; read dots represent reference point. P quadratic/Cochran Q = P-value for non-linearity from quadratic/Cochran Q test. Extremely sleep duration: <4h or >11h. A, Pancancer incidence; B, All-cause mortality of cancer patients; C, 5-year cancer mortality; D, Cancer mortality.

**Table S28. Analysis for mortality of cancer associated with sleep traits.**

|  | **All-cause mortality of cancer patients^§^** | | | **In 5-year cancer mortality** | | | **Cancer mortality^&^** | | |
| --- | --- | --- | --- | --- | --- | --- | --- | --- | --- |
| Sleep traits | No. | Deaths | % | No. | Deaths | % | No. | Deaths | % |
| Chronotype |  |  |  |  |  |  |  |  |  |
| Definite morning | 5,922 | 953 | 16.1% | 1,237 | 83 | 6.7% | 5,711 | 742 | 13.0% |
| More morning | 8,057 | 1,152 | 14.3% | 1,713 | 108 | 6.3% | 7,823 | 918 | 11.7% |
| More evening | 6,061 | 942 | 15.5% | 1,213 | 71 | 5.9% | 5,826 | 707 | 12.1% |
| Definite evening | 1,815 | 303 | 16.7% | 359 | 25 | 7.0% | 1,740 | 228 | 13.1% |
| Sleep duration |  |  |  |  |  |  |  |  |  |
| ≤5 hours | 1,206 | 215 | 17.8% | 235 | 21 | 8.9% | 1,148 | 157 | 13.7% |
| 6 hours | 4,160 | 671 | 16.1% | 833 | 57 | 6.8% | 4,021 | 532 | 13.2% |
| 7 hours | 8,712 | 1,185 | 13.6% | 1,805 | 95 | 5.3% | 8,465 | 938 | 11.1% |
| 8 hours | 7,462 | 1,076 | 14.4% | 1,592 | 88 | 5.5% | 7,211 | 825 | 11.4% |
| ≥9 hours | 2,376 | 530 | 22.3% | 487 | 52 | 10.7% | 2,250 | 404 | 18.0% |
| Insomnia symptom |  |  |  |  |  |  |  |  |  |
| Never | 5,261 | 721 | 13.7% | 1,115 | 62 | 5.6% | 5,090 | 550 | 10.8% |
| Sometimes | 11,153 | 1,638 | 14.7% | 2,338 | 152 | 6.5% | 10,794 | 1,279 | 11.8% |
| Usually | 7,536 | 1,327 | 17.6% | 1506 | 101 | 6.7% | 7,243 | 1,034 | 14.3% |

^§^ Mean (SD) of follow-up years: 11.63 (2.70); ^&^ Mean (SD) of follow-up years: 11.40 (2.44).

**Table S29. The analysis of survival probability of sleep traits on cancer patients among the UKB cohort (additional adjustment of full covariates).**


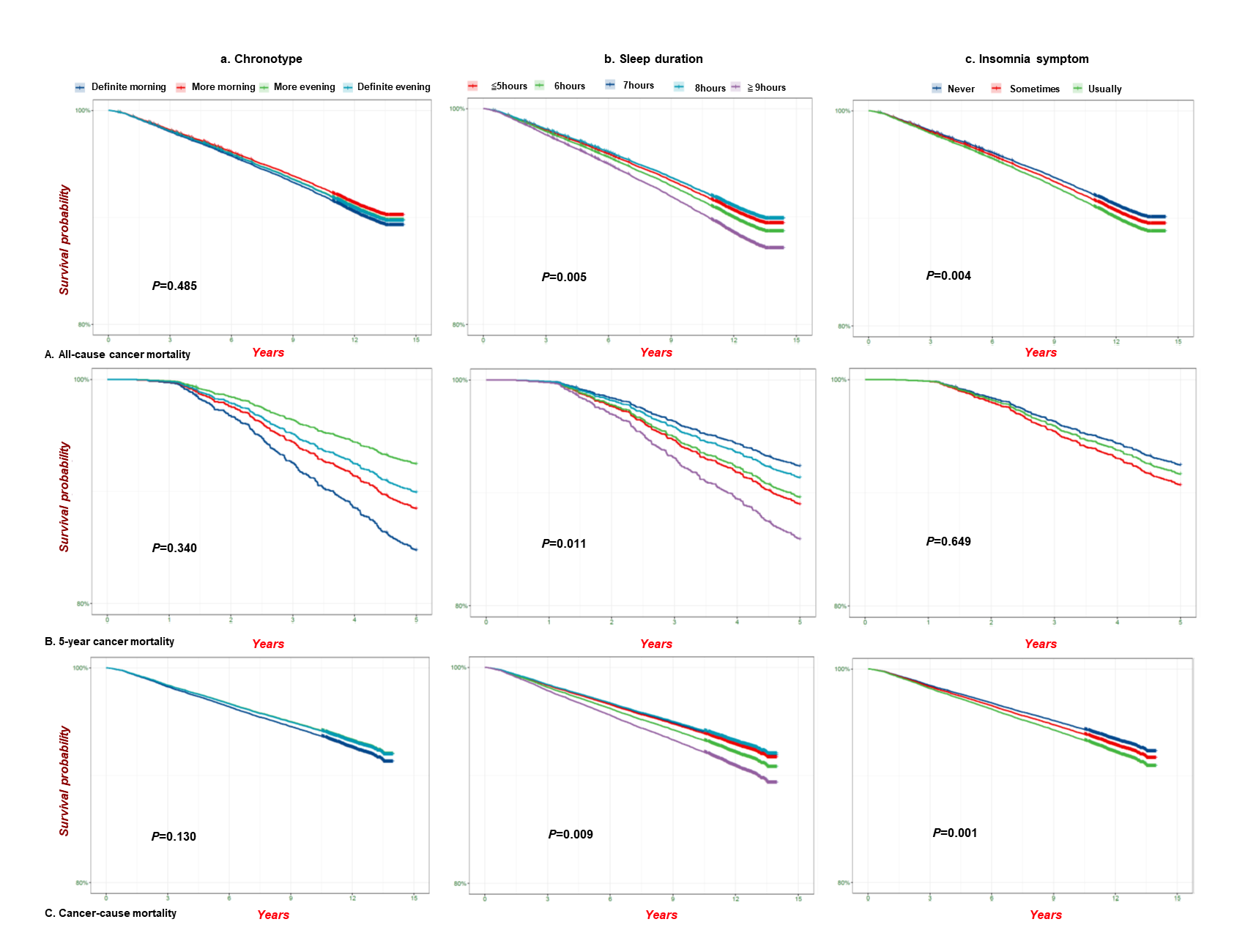


Kaplan–Meier plots for the survival probability of cancer patients in different group of sleep traits (chronotype, sleep duration and insomnia symptom) among the UKB cohort. P calculated by the log-rank test, and P < 0.05 for trend across different groups. Adjusted for age, sex, assessment center, top 10 genetic principal components, genotyping array, body mass index, employment status, Townsend deprivation index, smoking status, drinking status and mental health issues, vegTable Sand fruit intake, sedentary behavior, comorbidity, total physical activity, education, ethnicity, family history, number of self-reported cancers, operation, treatments and other sleep traits.

**Table S30. Multivariable Cox regression analysis for pancancer incidence and mortality associated with sleep traits among cancer patients after removing participants who reported currently working shifts.**

|  | **Pancancer incidence**  **(n=294,170)** | | **All-cause mortality**  **(n=22,570)** | | **5-year cancer mortality**  **(n=4,632)** | | **Cancer mortality**  **(n=21,774)** | |
| --- | --- | --- | --- | --- | --- | --- | --- | --- |
| Sleep traits | Hazard ratio (95%) | P value | Hazard ratio (95%) | P value | Hazard ratio (95%) | P value | Hazard ratio (95%) | P value |
| Chronotype |  |  |  |  |  |  |  |  |
| Definite morning | 1.00 (ref) | Ref | 1.00 (ref) | Ref | 1.00 (ref) | Ref | 1.00 (ref) | Ref |
| More morning | 0.99 (0.97-1.02) | 0.509 | 0.90 (0.82-0.98) | 0.020 | 0.91 (0.67-1.23) | 0.544 | 0.90 (0.82-1.00) | 0.047 |
| More evening | 0.99 (0.96-1.02) | 0.558 | 0.95 (0.86-1.04) | 0.280 | 0.81 (0.58-1.13) | 0.212 | 0.91 (0.82-1.01) | 0.075 |
| Definite evening | 1.04 (0.99-1.08) | 0.086 | 0.96 (0.84-1.10) | 0.540 | 0.84 (0.51-1.36) | 0.471 | 0.92 (0.78-1.07) | 0.269 |
| Sleep duration |  |  |  |  |  |  |  |  |
| 7 hours | 1.00 (ref) | Ref | 1.00 (ref) | Ref | 1.00 (ref) | Ref | 1.00 (ref) | Ref |
| ≤5 hours | 0.94 (0.89-0.99) | 0.014 | 1.00 (0.84-1.18) | 0.969 | 1.33 (0.74-2.41) | 0.337 | 0.95 (0.79-1.16) | 0.636 |
| 6 hours | 0.97 (0.94-1.00) | 0.032 | 1.11 (1.00-1.24) | 0.042 | 1.44 (1.01-2.07) | 0.045 | 1.12 (1.00-1.26) | 0.056 |
| 8 hours | 1.03 (1.00-1.05) | 0.033 | 1.03 (0.94-1.13) | 0.516 | 1.13 (0.82-1.55) | 0.458 | 1.01 (0.91-1.12) | 0.847 |
| ≥9 hours | 1.04 (0.99-1.08) | 0.084 | 1.30 (1.16-1.45) | <0.001 | 1.79 (1.21-2.65) | 0.004 | 1.35 (1.18-1.53) | <0.001 |
| Insomnia |  |  |  |  |  |  |  |  |
| Never | 1.00 (ref) | Ref | 1.00 (ref) | Ref | 1.00 (ref) | Ref | 1.00 (ref) | Ref |
| sometimes | 1.01 (0.98-1.03) | 0.595 | 1.11 (1.00-1.22) | 0.039 | 1.25 (0.90-1.74) | 0.182 | 1.16 (1.04-1.30) | 0.010 |
| Usually | 1.03 (1.00-1.06) | 0.068 | 1.19 (1.07-1.33) | 0.001 | 1.06 (0.73-1.52) | 0.772 | 1.28 (1.13-1.44) | <0.001 |

Pancancer incidence: Adjusted for age, sex, assessment center, top 10 genetic principal components, genotyping array, body mass index, employment status, Townsend deprivation index, smoking status, drinking status and mental health issues, vegTable Sand fruit intake, sedentary behavior, comorbidity, total physical activity, education, ethnicity, family history, and other sleep traits; Mortality: Adjusted for age, sex, assessment center, top 10 genetic principal components, genotyping array, body mass index, employment status, Townsend deprivation index, smoking status, drinking status and mental health issues, vegTable Sand fruit intake, sedentary behavior, comorbidity, total physical activity, education, ethnicity, family history, number of self-reported cancers, operation, treatments and other sleep traits. Statistical significance was defined as P < 0.05.

**Table S31. Multivariable Cox regression analysis for pancancer incidence and mortality associated with sleep traits among cancer patients after removing participants who reported extremely sleep duration (<4h or >11h).**

|  | **Pancancer incidence**  **(n=325,164)** | | **All-cause mortality**  **(n=23,832)** | | **5-year cancer mortality**  **(n=4,937)** | | **Cancer mortality**  **(n=23,029)** | |
| --- | --- | --- | --- | --- | --- | --- | --- | --- |
| Sleep traits | Hazard ratio (95%) | P value | Hazard ratio (95%) | P value | Hazard ratio (95%) | P value | Hazard ratio (95%) | P value |
| Chronotype |  |  |  |  |  |  |  |  |
| Definite morning | 1.00 (ref) | Ref | 1.00 (ref) | Ref | 1.00 (ref) | Ref | 1.00 (ref) | Ref |
| More morning | 0.99 (0.96-1.01) | 0.315 | 0.91 (0.84-0.99) | 0.037 | 0.96 (0.71-1.29) | 0.764 | 0.92 (0.83-1.01) | 0.078 |
| More evening | 0.99 (0.96-1.02) | 0.449 | 0.95 (0.87-1.04) | 0.301 | 0.84 (0.60-1.17) | 0.309 | 0.91 (0.82-1.01) | 0.088 |
| Definite evening | 1.03 (0.99-1.07) | 0.149 | 0.93 (0.81-1.06) | 0.288 | 0.87 (0.54-1.40) | 0.569 | 0.90 (0.77-1.05) | 0.177 |
| Sleep duration |  |  |  |  |  |  |  |  |
| 7 hours | 1.00 (ref) | Ref | 1.00 (ref) | Ref | 1.00 (ref) | Ref | 1.00 (ref) | Ref |
| ≤5 hours | 0.94 (0.90-0.99) | 0.025 | 1.00 (0.85-1.18) | 0.976 | 1.35 (0.76-2.38) | 0.304 | 0.98 (0.81-1.18) | 0.823 |
| 6 hours | 0.97 (0.94-1.00) | 0.037 | 1.13 (1.02-1.25) | 0.019 | 1.49 (1.06-2.11) | 0.023 | 1.13 (1.01-1.27) | 0.034 |
| 8 hours | 1.03 (1.00-1.05) | 0.021 | 1.02 (0.93-1.11) | 0.696 | 1.13 (0.82-1.54) | 0.451 | 1.00 (0.90-1.10) | 0.987 |
| ≥9 hours | 1.05 (1.01-1.09) | 0.023 | 1.27 (1.13-1.42) | <0.001 | 1.81 (1.23-2.67) | <0.001 | 1.32 (1.16-1.51) | <0.001 |
| Insomnia |  |  |  |  |  |  |  |  |
| Never | 1.00 (ref) | Ref | 1.00 (ref) | Ref | 1.00 (ref) | Ref | 1.00 (ref) | Ref |
| sometimes | 1.01 (0.99-1.04) | 0.387 | 1.10 (1.00-1.21) | 0.042 | 1.27 (0.92-1.75) | 0.149 | 1.15 (1.03-1.28) | 0.015 |
| Usually | 1.03 (1.00-1.06) | 0.034 | 1.18 (1.07-1.31) | 0.001 | 1.12 (0.78-1.60) | 0.537 | 1.26 (1.12-1.42) | <0.001 |

Pancancer incidence: Adjusted for age, sex, assessment center, top 10 genetic principal components, genotyping array, body mass index, employment status, Townsend deprivation index, smoking status, drinking status and mental health issues, vegTable Sand fruit intake, sedentary behavior, comorbidity, total physical activity, education, ethnicity, family history, and other sleep traits; Mortality: Adjusted for age, sex, assessment center, top 10 genetic principal components, genotyping array, body mass index, employment status, Townsend deprivation index, smoking status, drinking status and mental health issues, vegTable Sand fruit intake, sedentary behavior, comorbidity, total physical activity, education, ethnicity, family history, number of self-reported cancers, operation, treatments and other sleep traits. Statistical significance was defined as P < 0.05.

**Table S32. Multivariable Cox regression analysis for pancancer incidence and mortality associated with sleep traits among age > 50 cancer patients.**

|  | **Pancancer incidence**  **(n=231,556)** | | **All-cause mortality**  **(n=16,450)** | | **5-year cancer mortality**  **(n=4,325)** | | **Cancer mortality**  **(n=15,768)** | |
| --- | --- | --- | --- | --- | --- | --- | --- | --- |
| Sleep traits | Hazard ratio (95%) | P value | Hazard ratio (95%) | P value | Hazard ratio (95%) | P value | Hazard ratio (95%) | P value |
| Chronotype |  |  |  |  |  |  |  |  |
| Definite morning | 1.00 (ref) | Ref | 1.00 (ref) | Ref | 1.00 (ref) | Ref | 1.00 (ref) | Ref |
| More morning | 0.99 (0.96-1.01) | 0.353 | 0.96 (0.87-1.06) | 0.383 | 1.00 (0.73-1.37) | 0.997 | 0.98 (0.88-1.10) | 0.791 |
| More evening | 0.99 (0.96-1.02) | 0.552 | 0.99 (0.89-1.10) | 0.899 | 0.92 (0.65-1.31) | 0.641 | 0.98 (0.86-1.10) | 0.696 |
| Definite evening | 1.06 (1.01-1.10) | 0.012 | 1.03 (0.88-1.20) | 0.718 | 1.05 (0.65-1.70) | 0.853 | 1.04 (0.87-1.24) | 0.672 |
| Sleep duration |  |  |  |  |  |  |  |  |
| 7 hours | 1.00 (ref) | Ref | 1.00 (ref) | Ref | 1.00 (ref) | Ref | 1.00 (ref) | Ref |
| ≤5 hours | 0.94 (0.90-1.00) | 0.039 | 1.11 (0.93-1.33) | 0.249 | 1.62 (0.93-2.28) | 0.090 | 1.07 (0.87-1.33) | 0.508 |
| 6 hours | 0.98 (0.95-1.01) | 0.131 | 1.14 (1.02-1.29) | 0.024 | 1.38 (0.95-2.01) | 0.088 | 1.14 (0.99-1.30) | 0.062 |
| 8 hours | 1.03 (1.01-1.06) | 0.016 | 1.03 (0.93-1.14) | 0.544 | 1.13 (0.81-1.58) | 0.483 | 1.01 (0.90-1.14) | 0.833 |
| ≥9 hours | 1.03 (0.99-1.07) | 0.188 | 1.34 (1.19-1.53) | <0.001 | 1.97 (1.32-2.95) | 0.001 | 1.41 (1.22-1.63) | <0.001 |
| Insomnia |  |  |  |  |  |  |  |  |
| Never | 1.00 (ref) | Ref | 1.00 (ref) | Ref | 1.00 (ref) | Ref | 1.00 (ref) | Ref |
| sometimes | 1.01 (0.99-1.04) | 0.347 | 1.07 (0.96-1.20) | 0.191 | 1.22 (0.87-1.72) | 0.250 | 1.09 (0.97-1.24) | 0.154 |
| Usually | 1.04 (1.00-1.07) | 0.024 | 1.17 (1.04-1.32) | 0.008 | 1.10 (0.76-1.60) | 0.622 | 1.24 (1.08-1.41) | 0.002 |

Pancancer incidence: Adjusted for age, sex, assessment center, top 10 genetic principal components, genotyping array, body mass index, employment status, Townsend deprivation index, smoking status, drinking status and mental health issues, vegTable Sand fruit intake, sedentary behavior, comorbidity, total physical activity, education, ethnicity, family history, and other sleep traits; Mortality: Adjusted for age, sex, assessment center, top 10 genetic principal components, genotyping array, body mass index, employment status, Townsend deprivation index, smoking status, drinking status and mental health issues, vegTable Sand fruit intake, sedentary behavior, comorbidity, total physical activity, education, ethnicity, family history, number of self-reported cancers, operation, treatments and other sleep traits. Statistical significance was defined as P < 0.05.

**Table S33. The analysis of survival probability of sleep traits on pancancer patients (age>50 years) among the UKB cohort.**

**
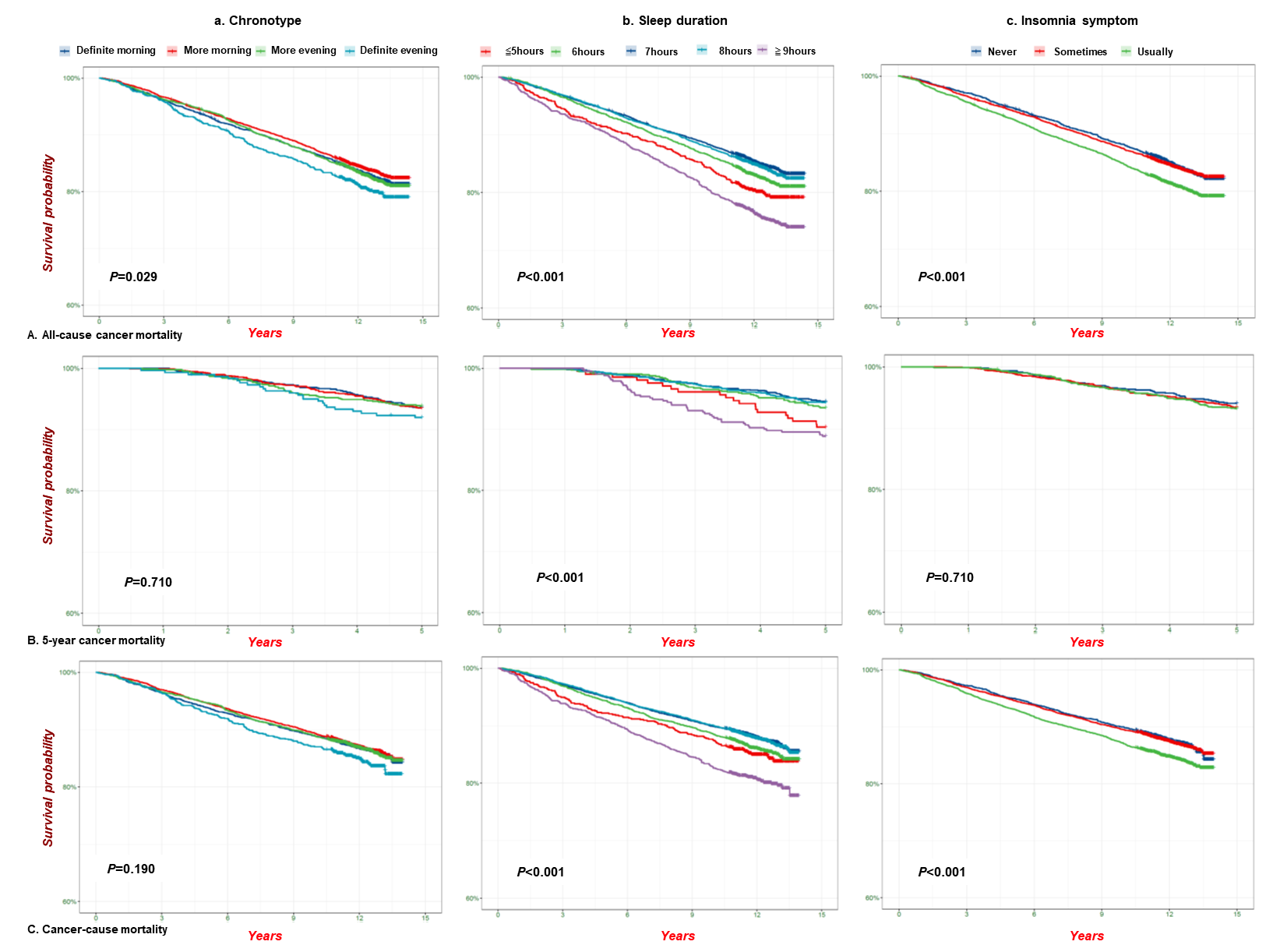
**

Kaplan–Meier plots for the survival probability of cancer patients (age＞50 years ) in different group of sleep traits (chronotype, sleep duration and insomnia symptom) among the UKB cohort. P calculated by the log-rank test, and P < 0.05 for trend across different groups. All-cause mortality (n=16,450); 5-year cancer mortality (n=4,325); Cancer mortality (n=15,768).

**Table S34. The analysis of survival probability of sleep traits on pancancer patients (age>50 years) among the UKB cohort (additional adjustment of full covariates).**


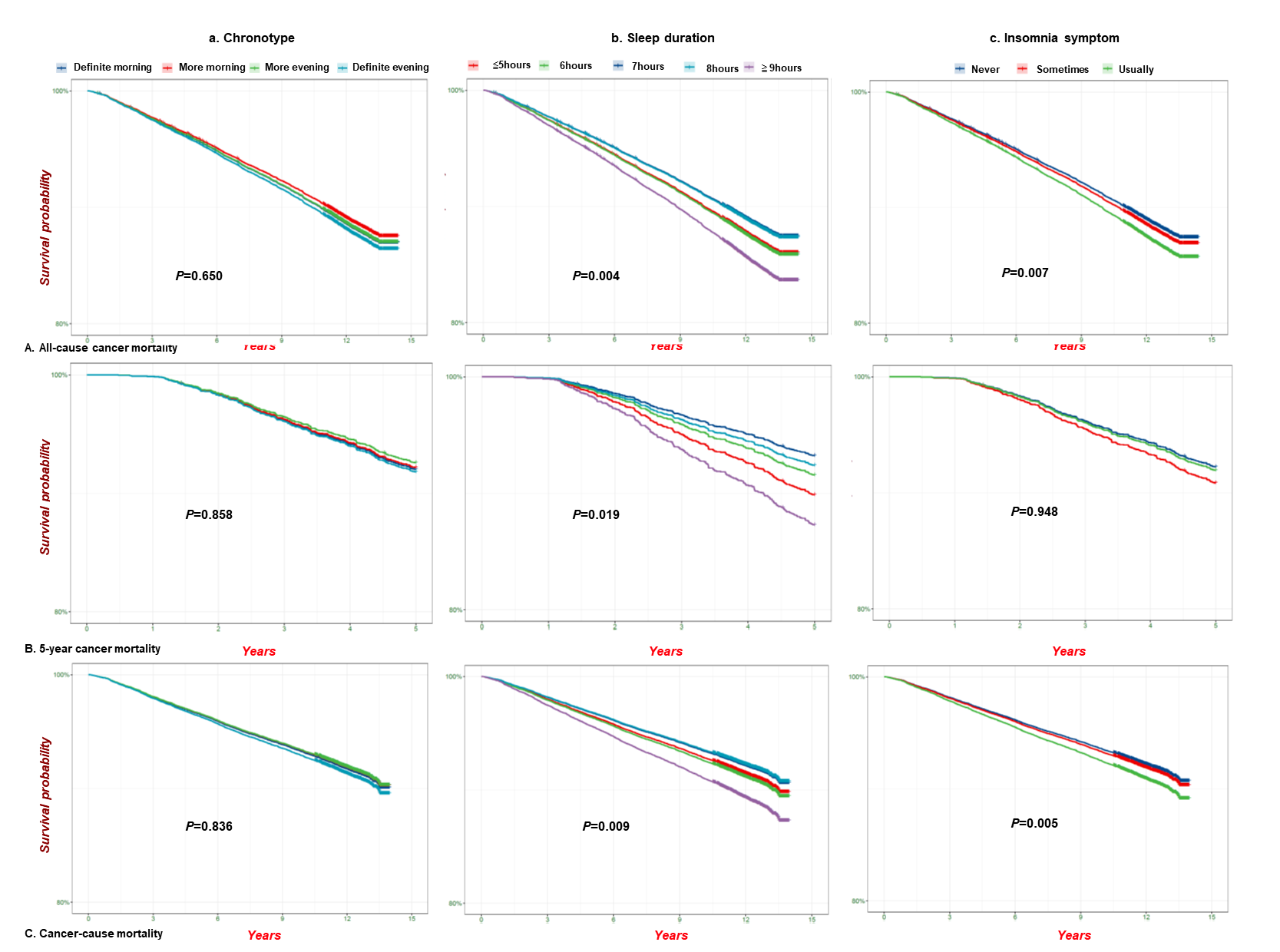


Kaplan–Meier plots for the survival probability of cancer patients (age＞50 years ) in different group of sleep traits (chronotype, sleep duration and insomnia symptom) among the UKB cohort. P calculated by the log-rank test, and P < 0.001 for trend across different groups. Adjusted for age, sex, assessment center, top 10 genetic principal components, genotyping array, body mass index, employment status, Townsend deprivation index, smoking status, drinking status and mental health issues, vegTable Sand fruit intake, sedentary behavior, comorbidity, total physical activity, education, ethnicity, family history, number of self-reported cancers, operation, treatments and other sleep traits. All-cause mortality (n=16,450); 5-year cancer mortality (n=4,325); Cancer mortality (n=15,768).

**Table S35. The test for a quadratic fit of the results on association between sleep duration and all-cause mortality among cancer patients (Figure 2b).**

A. Basic model


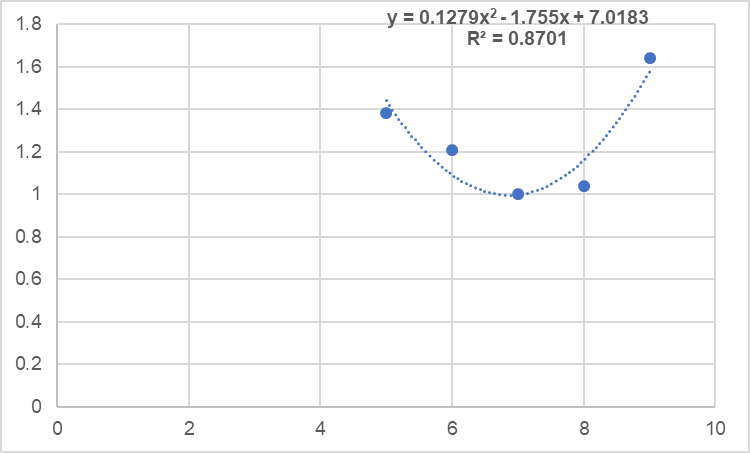


B. Further adjusted model


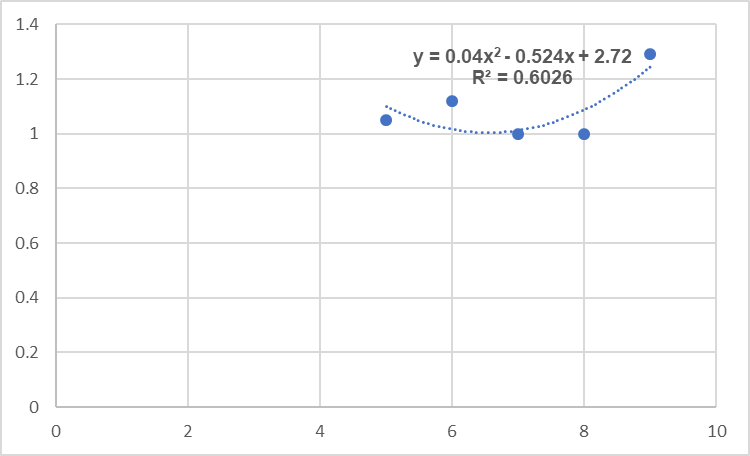


C. Full model


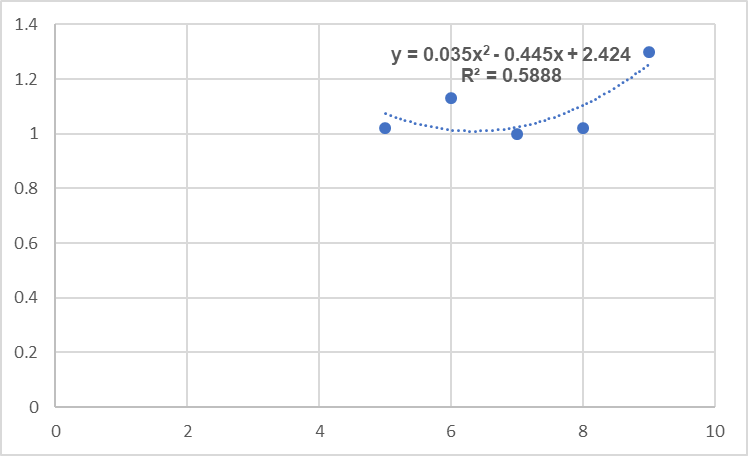


**Table S36. The test for a quadratic fit of the results on association between sleep duration and 5-year cancer mortality among cancer patients (Figure 2c).**

A. Basic model


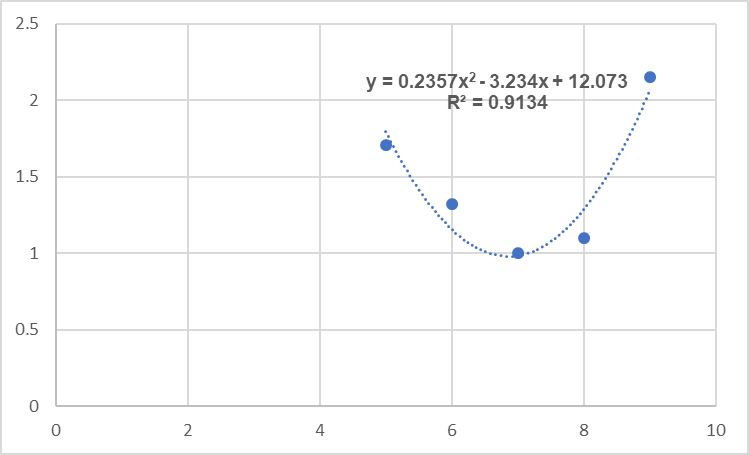


B. Further adjusted model


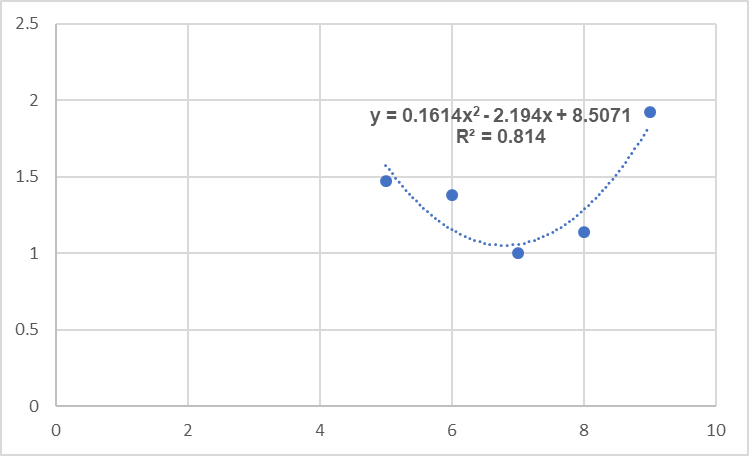


C. Full model


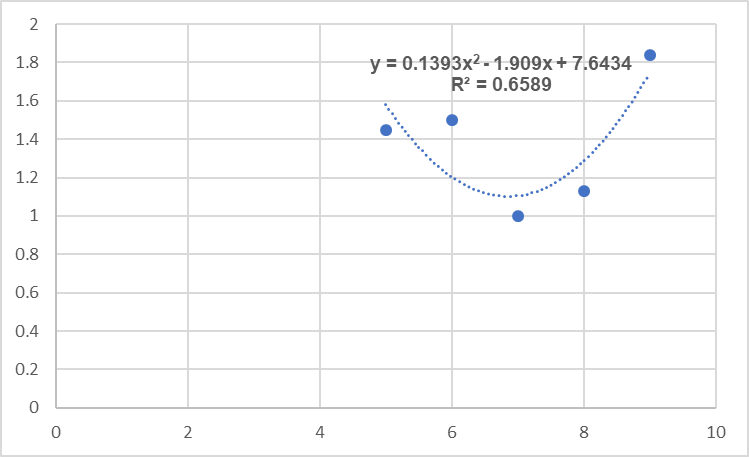


**Table S37. The test for a quadratic fit of the results on association between sleep duration and cancer-cause mortality among cancer patients (Figure 2d).**

A. Basic model


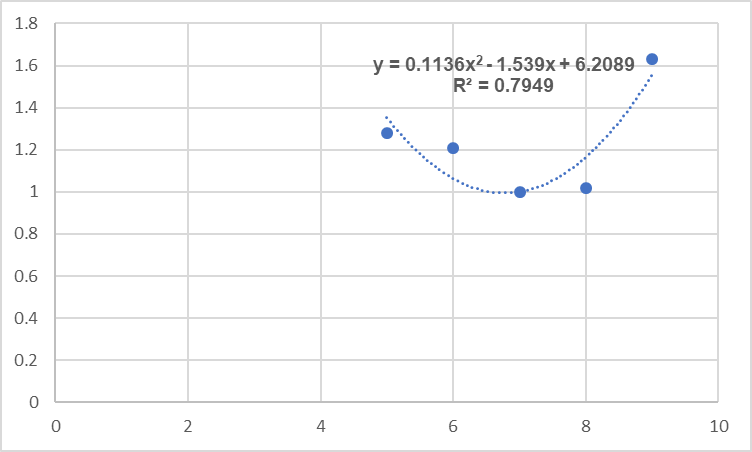


B. Further adjusted model


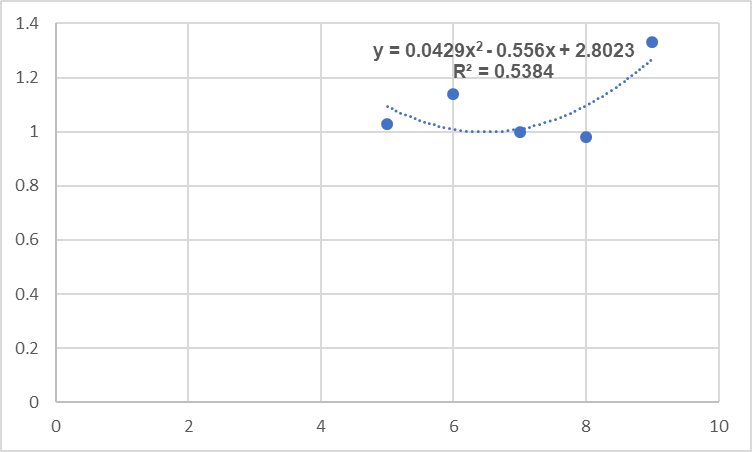


C. Full model


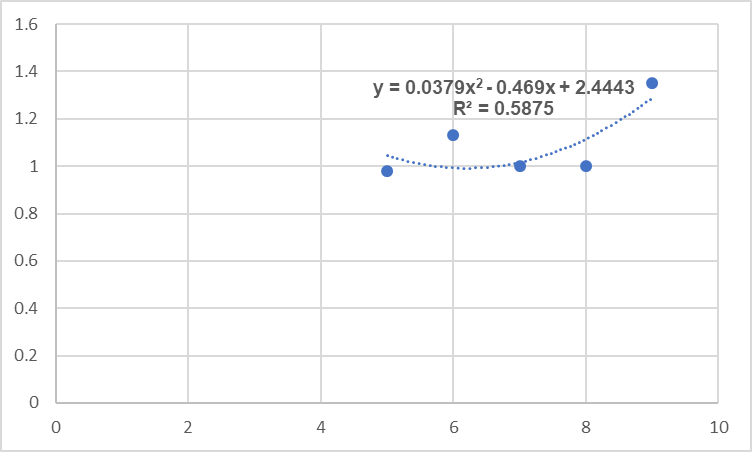


**Table S38. Scatter plot of individual SNP-sleep traits and SNP effect on pancancer incidence associations with overlay of causal estimate from each MR test using.**


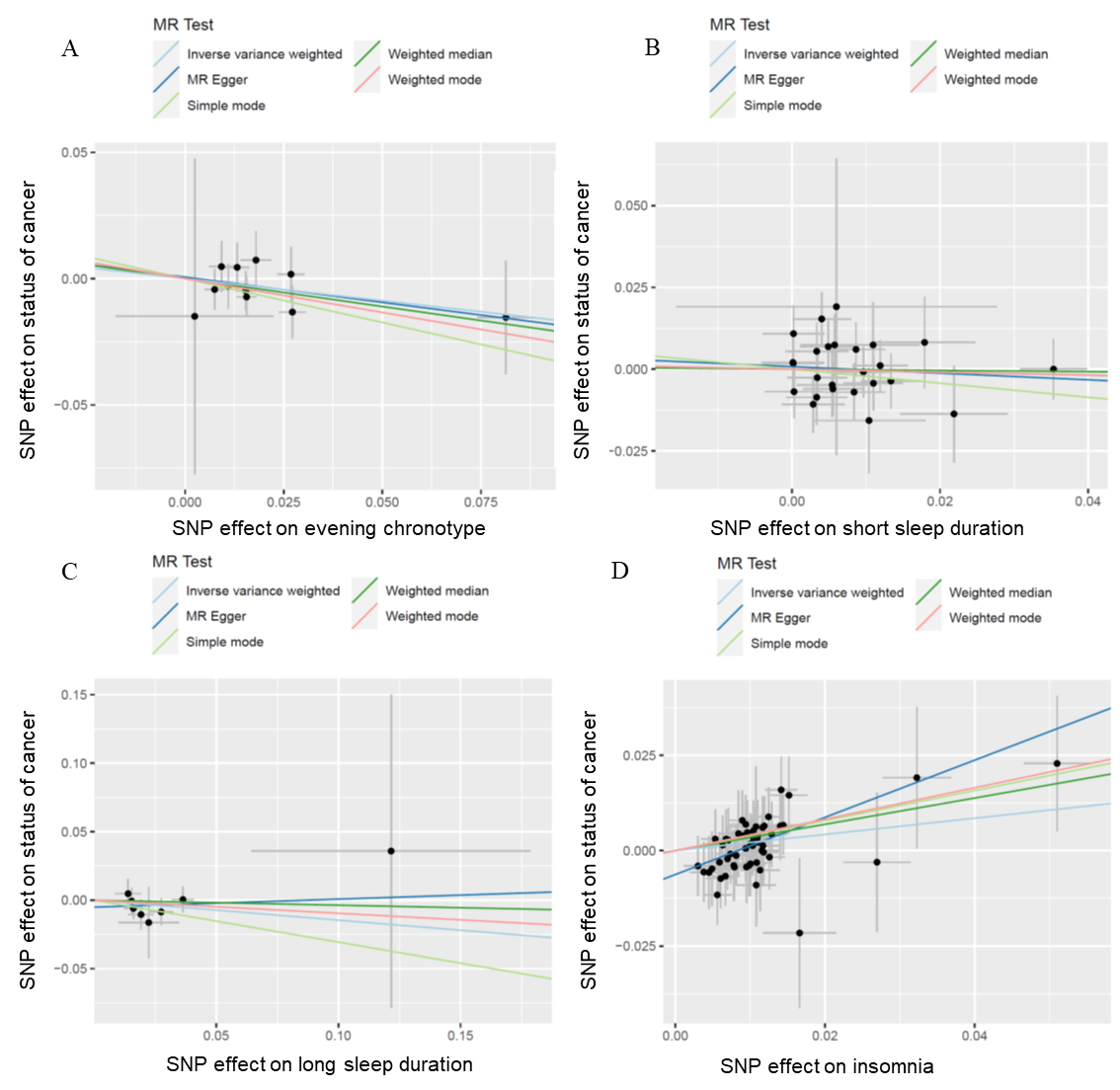


**Table S39. Scatter plot of individual SNP-sleep traits and SNP effect on all-cause mortality of cancer patient associations with overlay of causal estimate from each MR test using.**


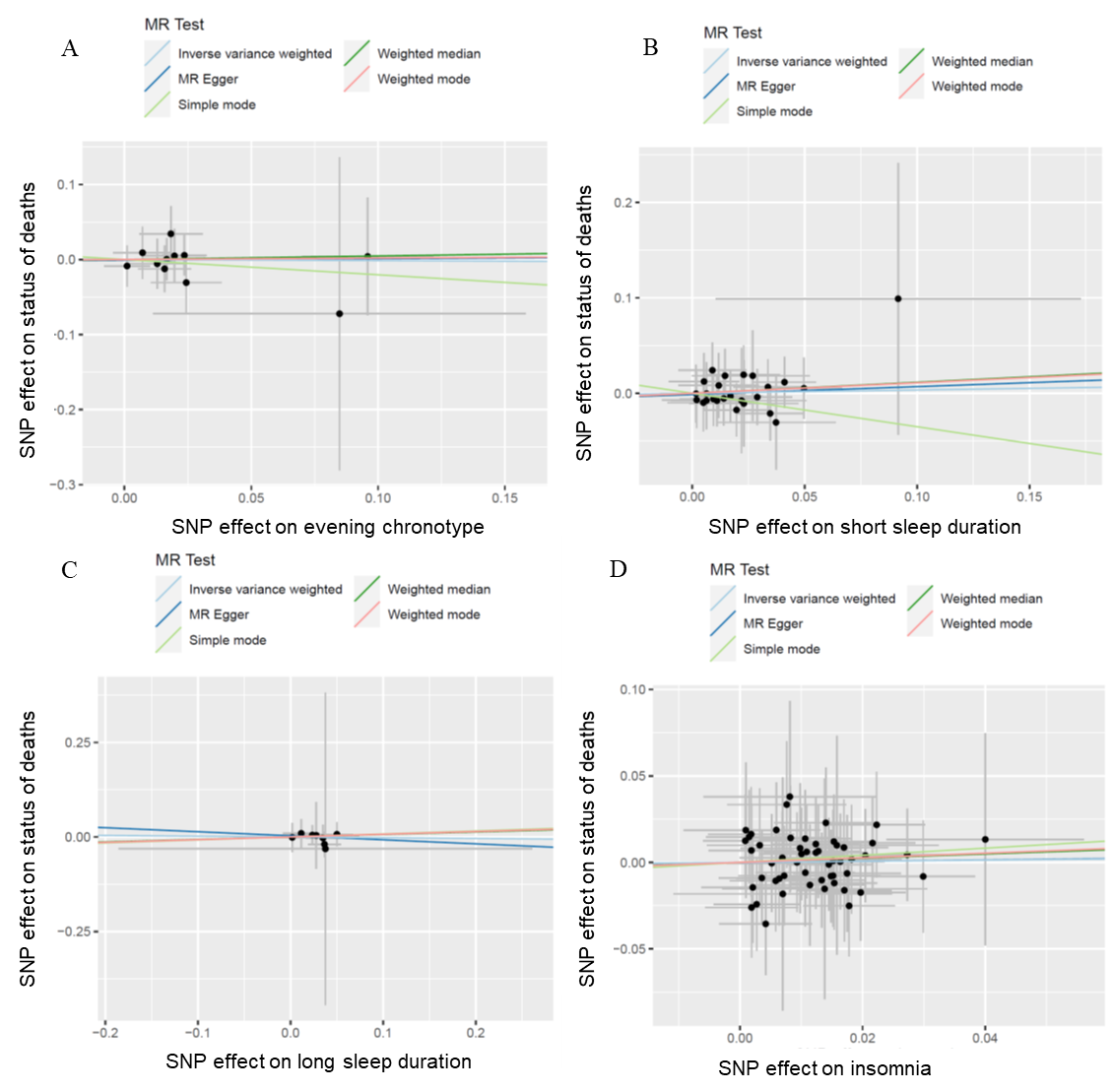


**Table S40. Scatter plot of individual SNP-sleep traits and SNP effect on 5-year cancer mortality of cancer patient associations with overlay of causal estimate from each MR test using.**


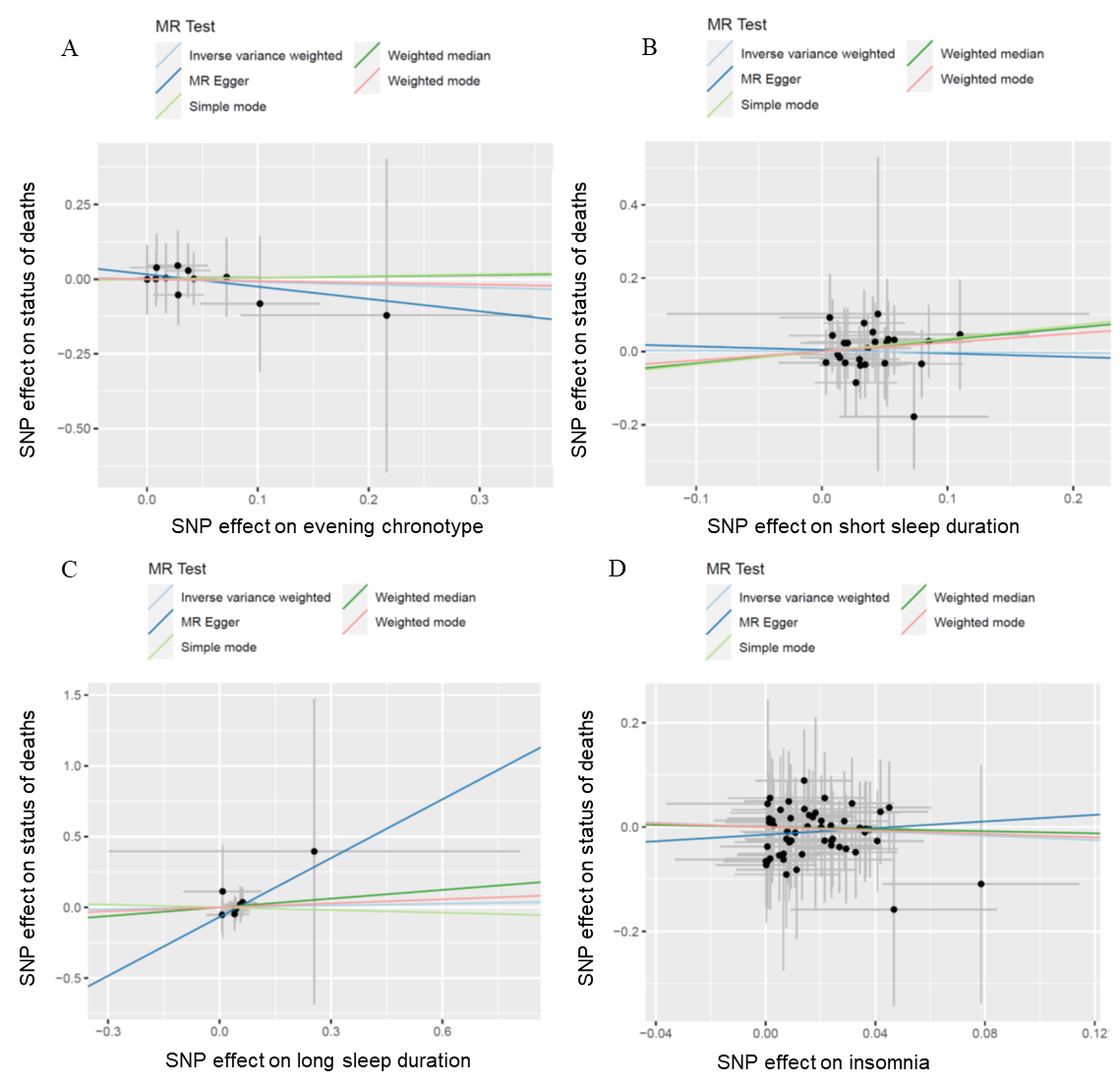


**Table S41. Scatter plot of individual SNP-sleep traits and SNP effect on cancer mortality of cancer patient associations with overlay of causal estimate from each MR test using.**


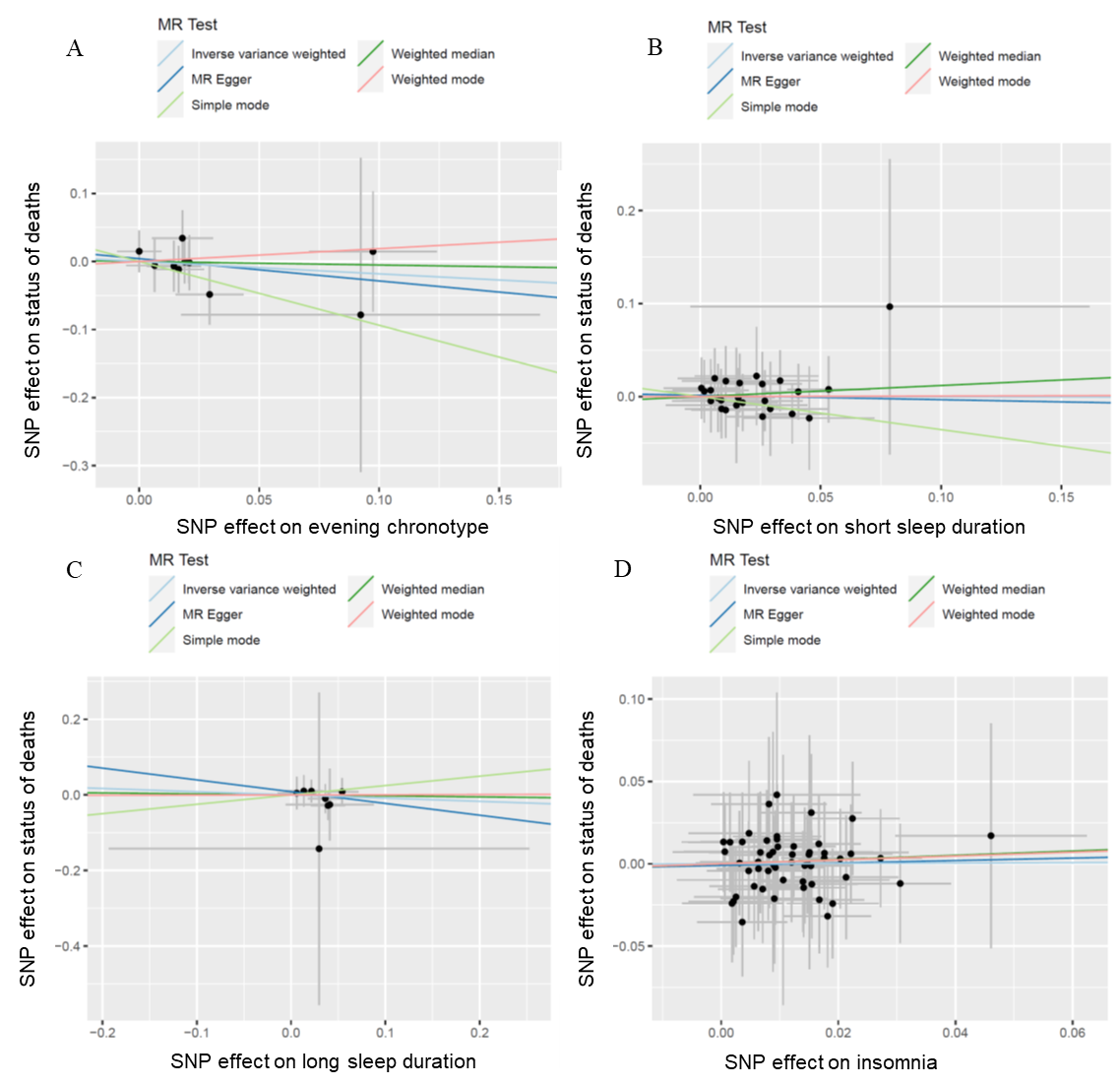


**Table S42. Radial MR plots for sleep traits in pancancer incidence.**


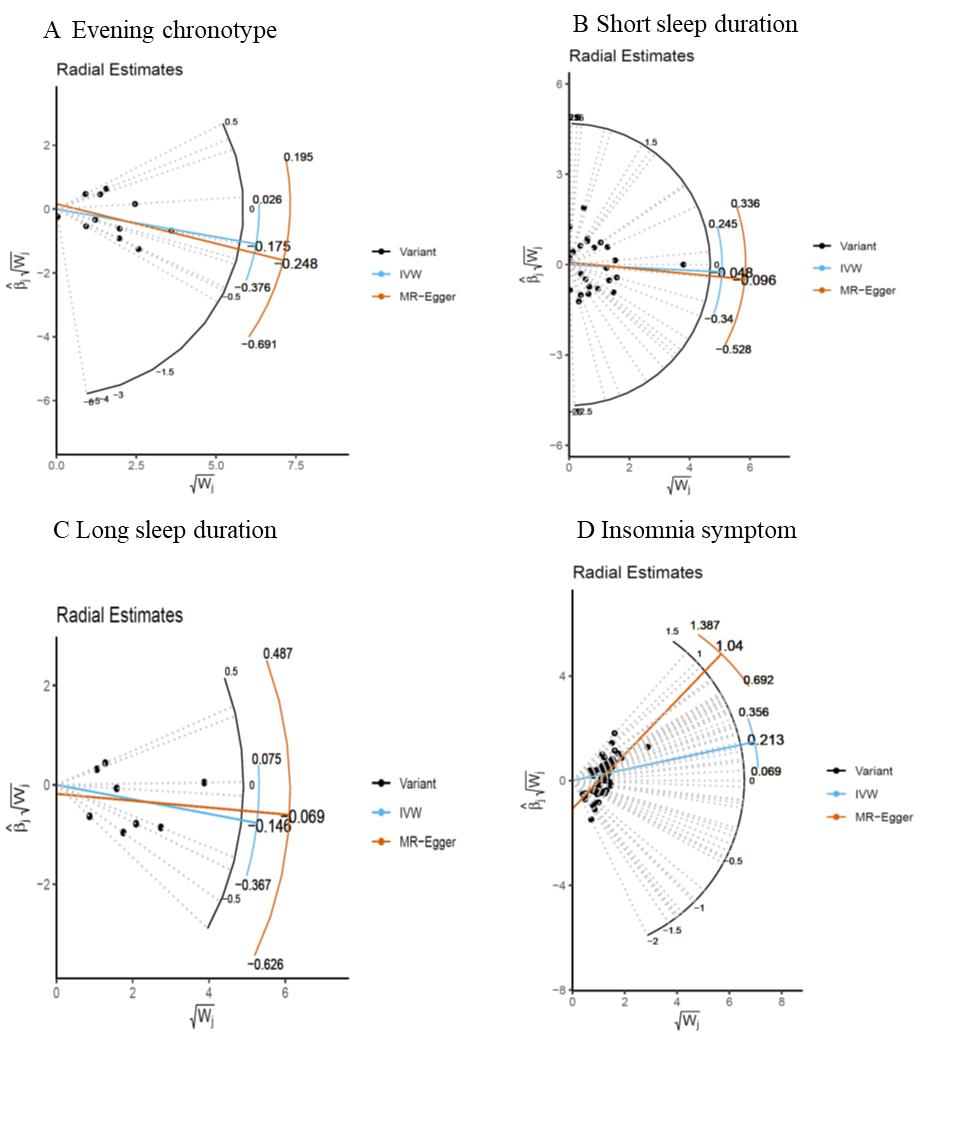


Radial curve means the ratio estimate for each SNP. No outlying genetic variants were found.

**Table S43. Radial MR plots for sleep traits in all-cause mortality among cancer patients.**


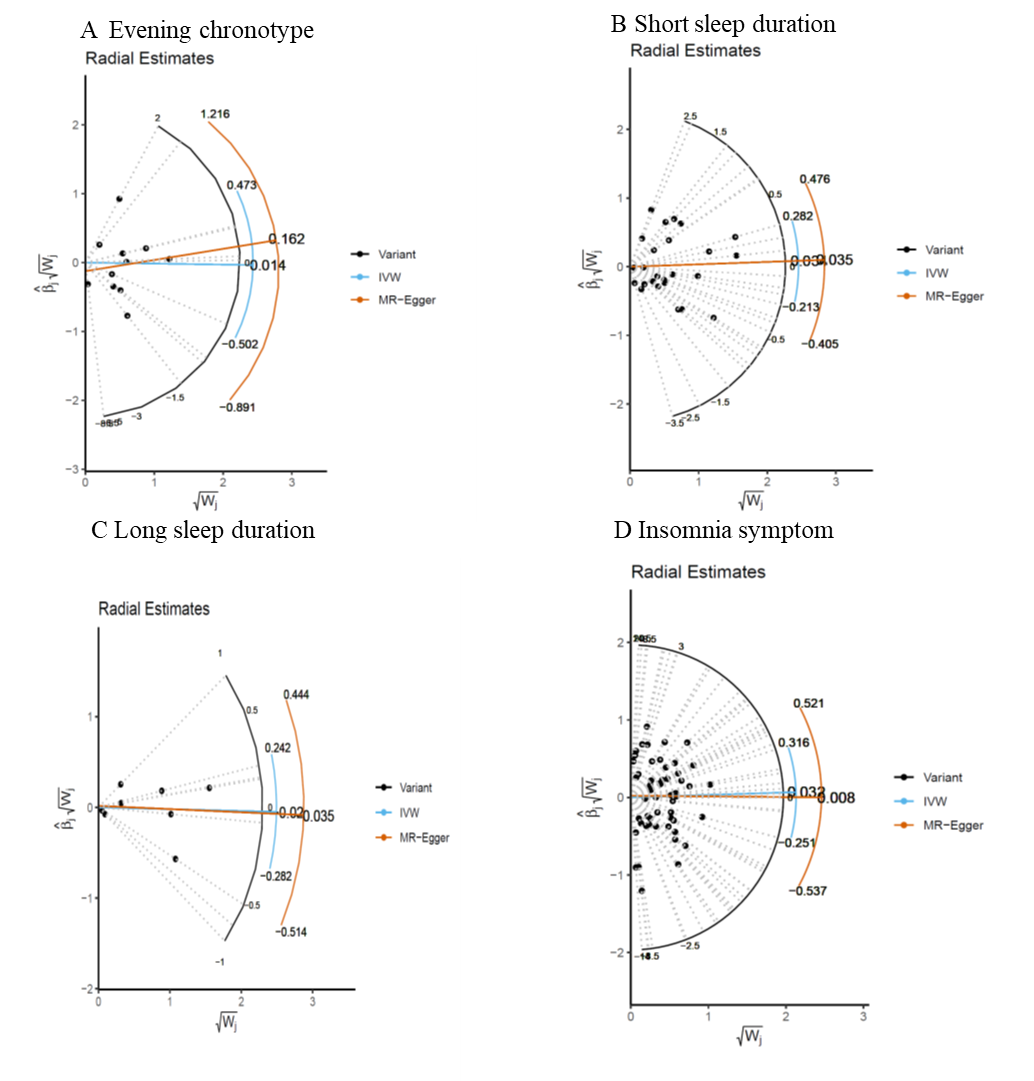


Radial curve means the ratio estimate for each SNP. No outlying genetic variants were found.

**Table S44. Radial MR plots for sleep traits in 5-year cancer mortality among cancer patients.**


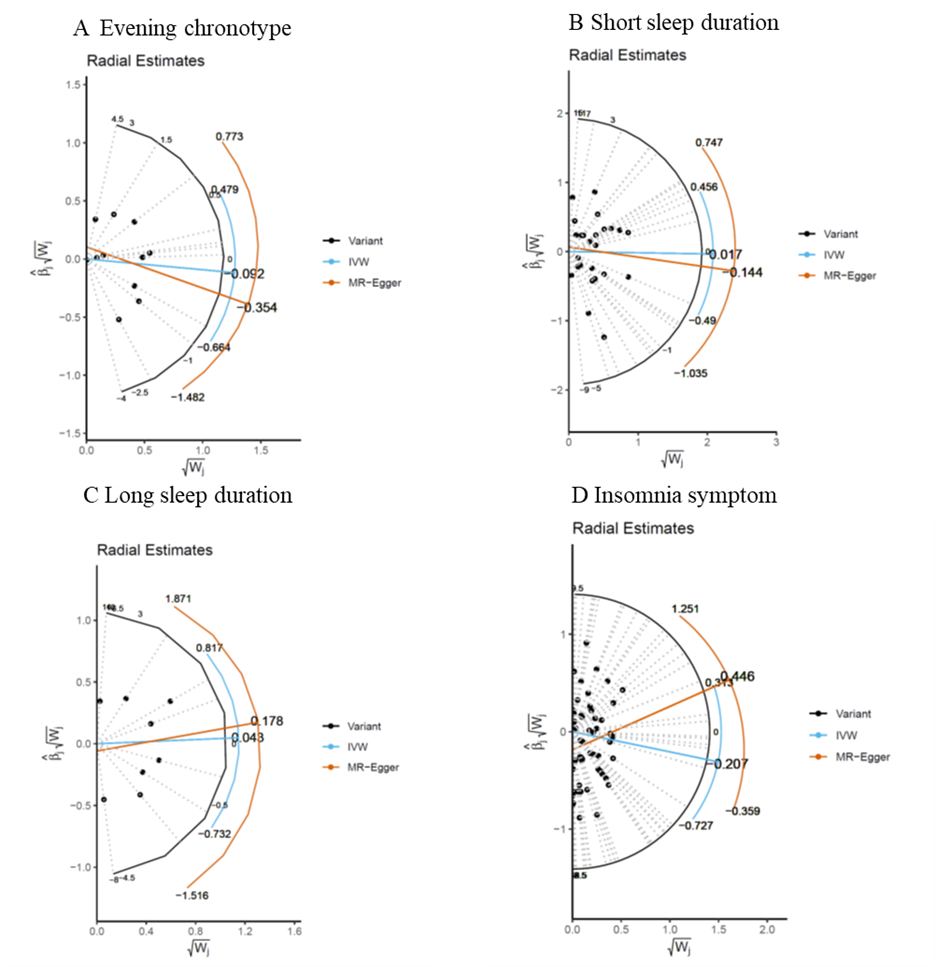


Radial curve means the ratio estimate for each SNP. No outlying genetic variants were found.

**Table S45. Radial MR plots for sleep traits in cancer-cause mortality among cancer patients.**

**
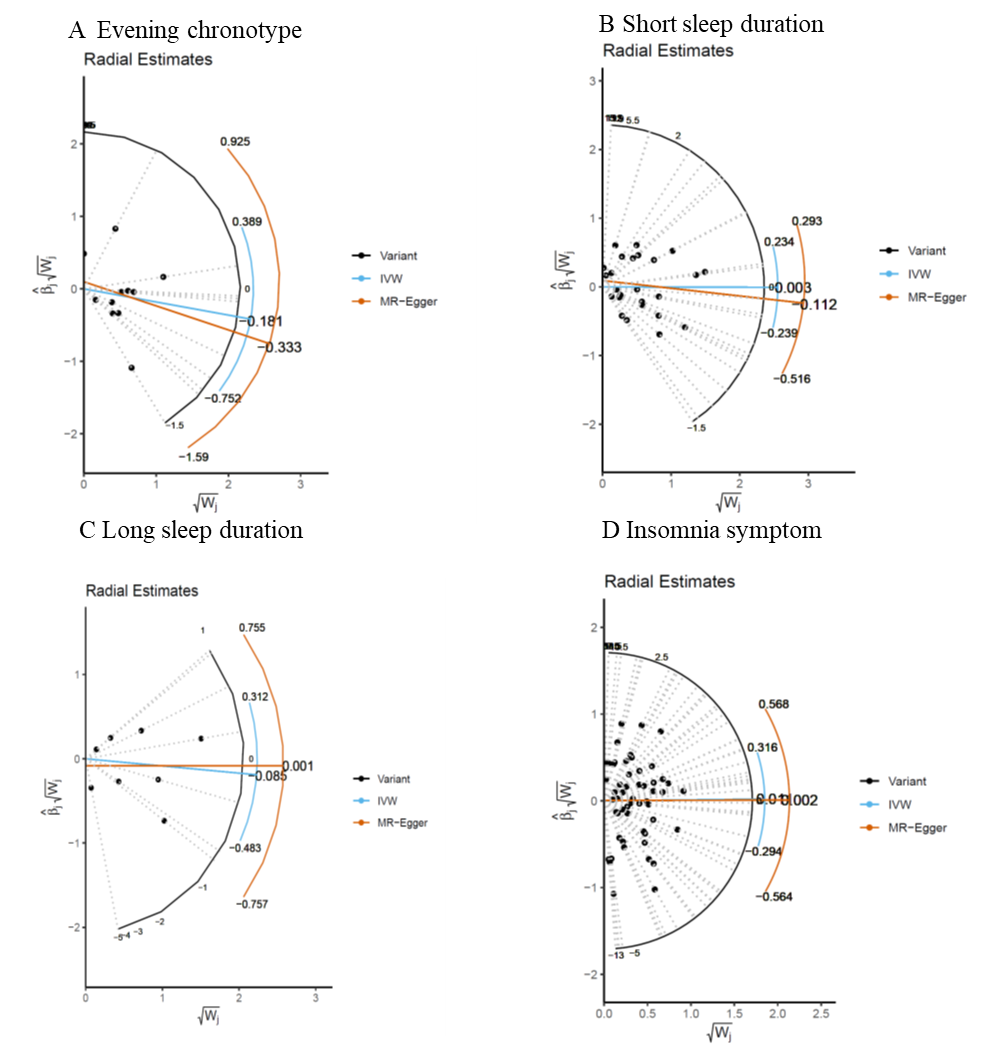
**

Radial curve means the ratio estimate for each SNP. No outlying genetic variants were found in these cancers.

**Table S46. MR leave-one-out sensitivity analysis for the effect of the evening chronotype SNPs on outcomes.**


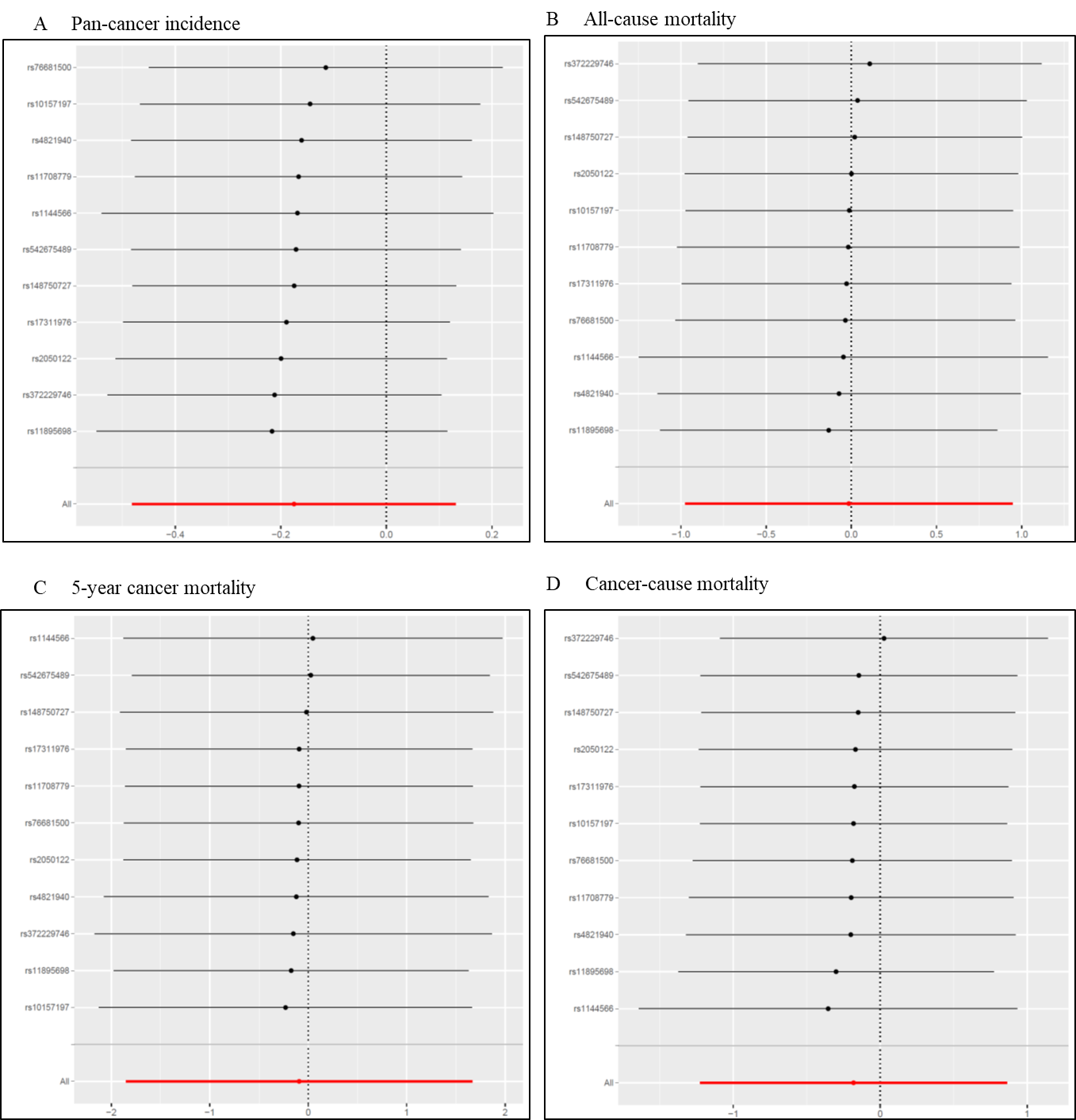


A. MR leave−one−out sensitivity analysis for 'evening chronotype' on 'pancancer incidence'. B. MR leave−one−out sensitivity analysis for 'evening chronotype' on 'all-cause deaths' among cancer patients. C. MR leave−one−out sensitivity analysis for 'evening chronotype' on '5-year cancer deaths' among cancer patients. D. MR leave−one−out sensitivity analysis for 'evening chronotype' on 'cancer-cause deaths' among cancer patients.

**Table S47. MR leave-one-out sensitivity analysis for the effect of the short sleep duration SNPs on outcomes.**


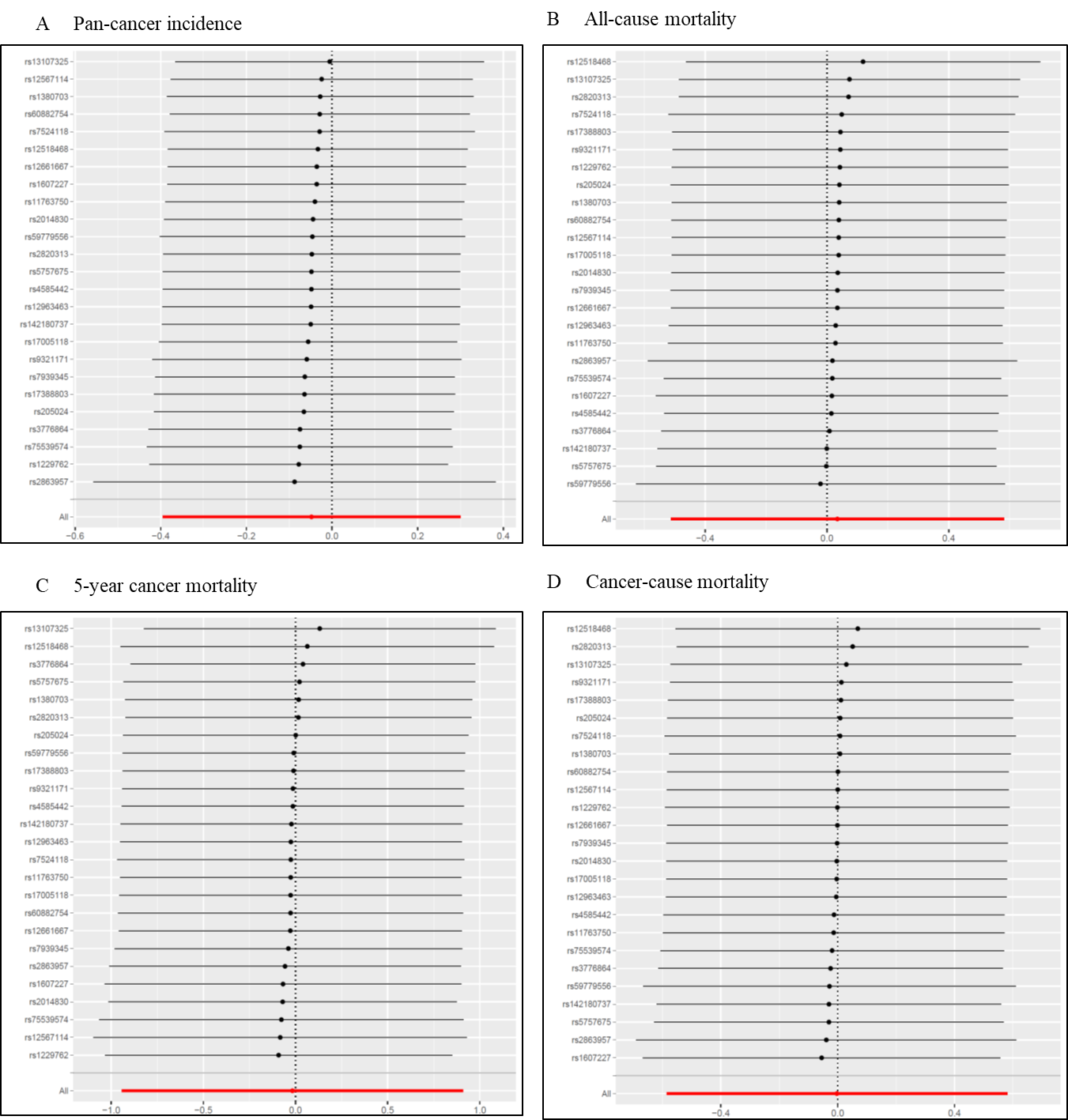


A. MR leave−one−out sensitivity analysis for 'short sleep duration' on 'pancancer incidence'. B. MR leave−one−out sensitivity analysis for 'short sleep duration' on 'all-cause deaths' among cancer patients. C. MR leave−one−out sensitivity analysis for 'short sleep duration' on '5-year cancer deaths' among cancer patients. D. MR leave−one−out sensitivity analysis for 'short sleep duration' on 'cancer-cause deaths' among cancer patients.

**Table S48. MR leave-one-out sensitivity analysis for the effect of the long sleep duration SNPs on outcomes.**


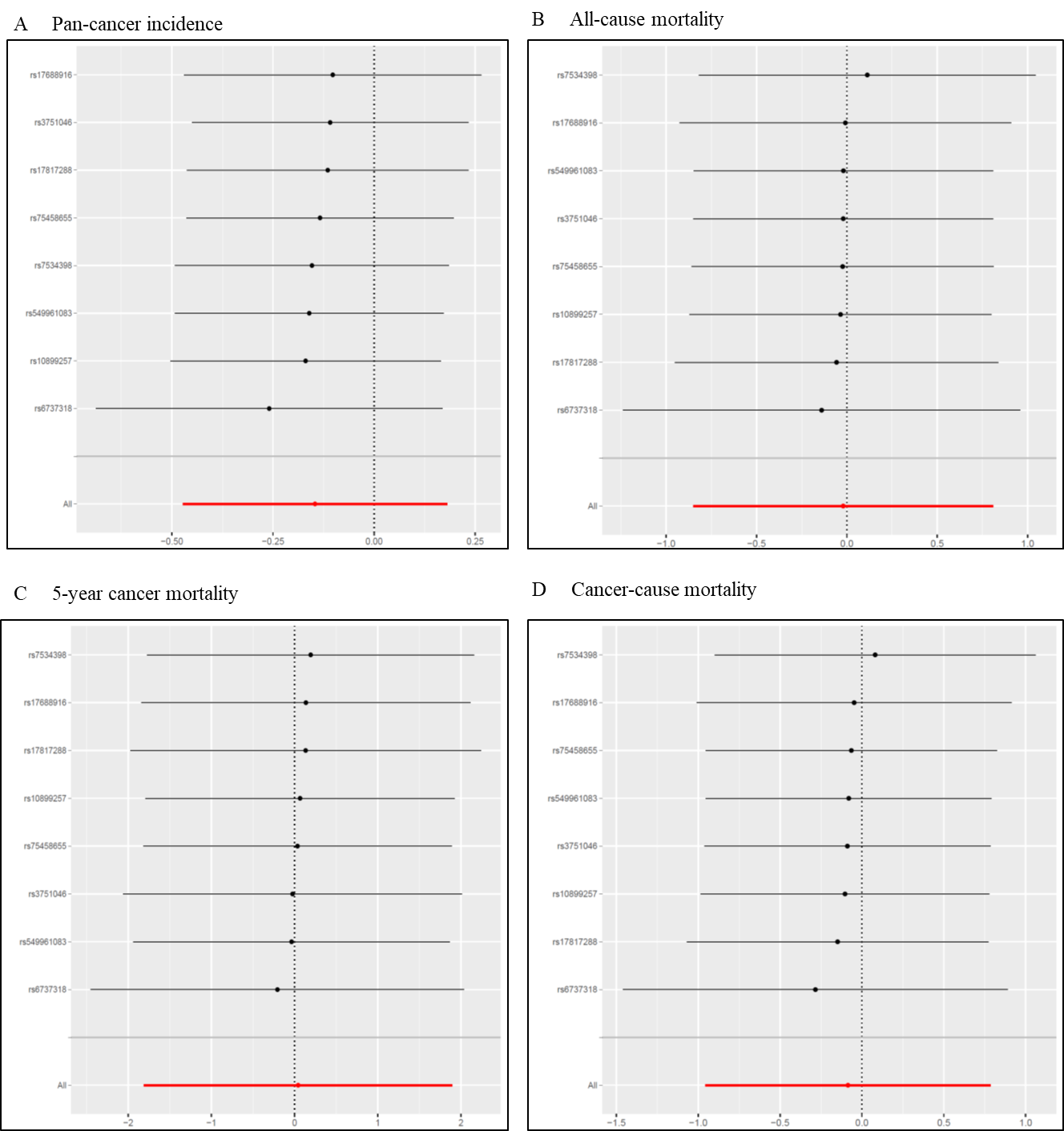


A. MR leave−one−out sensitivity analysis for 'long sleep duration' on 'pancancer incidence'. B. MR leave−one−out sensitivity analysis for 'long sleep duration' on 'all-cause deaths' among cancer patients. C. MR leave−one−out sensitivity analysis for 'long sleep duration' on '5-year cancer deaths' among cancer patients. D. MR leave−one−out sensitivity analysis for 'long sleep duration' on 'cancer-cause deaths' among cancer patients.

**Table S49. MR leave-one-out sensitivity analysis for the effect of the insomnia SNPs on outcomes.**


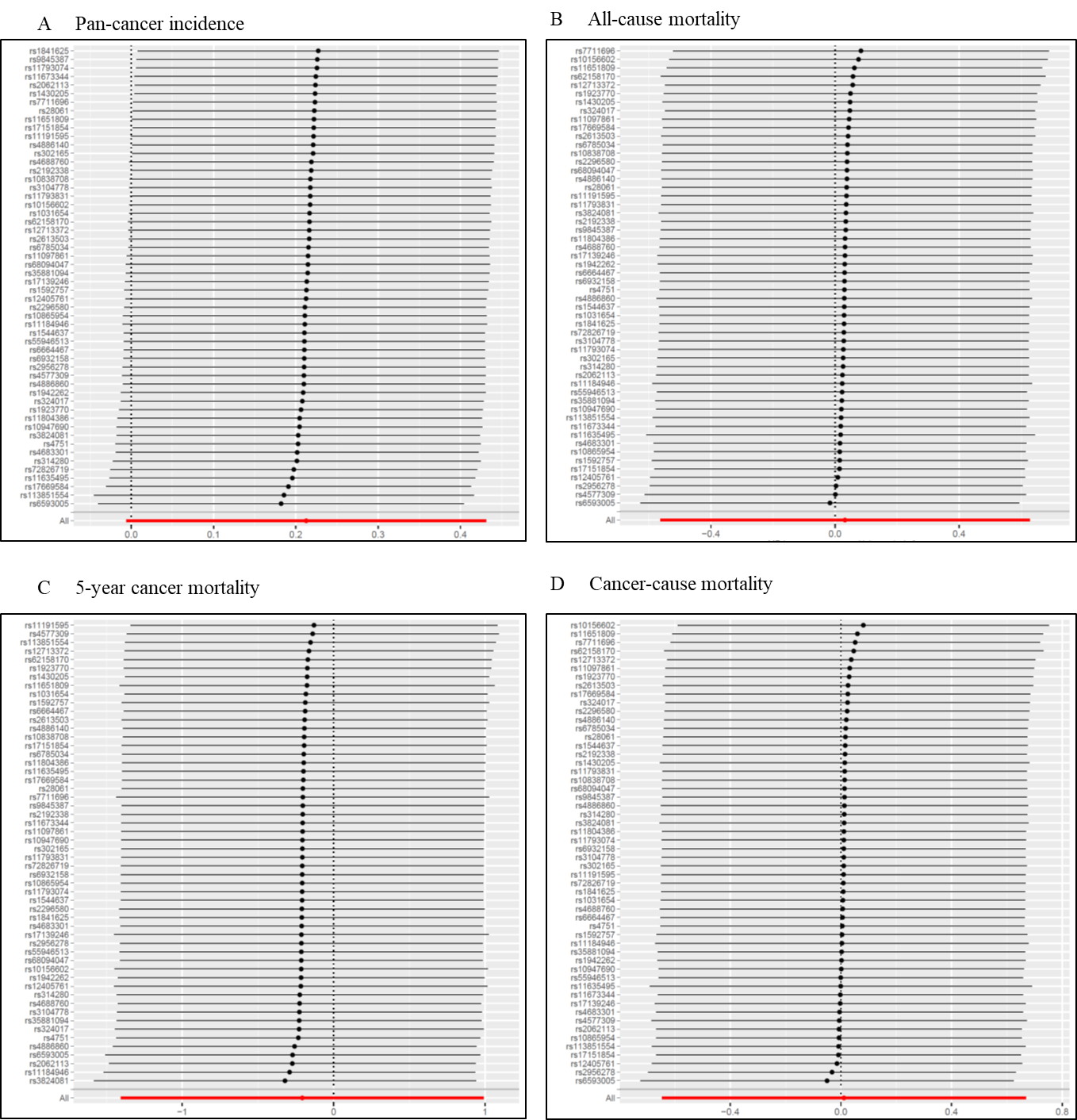


A. MR leave−one−out sensitivity analysis for 'insomnia' on 'pancancer incidence'. B. MR leave−one−out sensitivity analysis for 'insomnia' on 'all-cause deaths' among cancer patients. C. MR leave−one−out sensitivity analysis for 'insomnia' on '5-year cancer deaths' among cancer patients. D. MR leave−one−out sensitivity analysis for 'insomnia' on 'cancer-cause deaths' among cancer patients.

**Table S50. Sensitivity analysis for evening chronotype and pancancer incidence and mortality of cancer patients using the Weighted median and MR-Egger methods.**

| **Outcomes** | **Weighted median** | | **MR-Egger** | | | |
| --- | --- | --- | --- | --- | --- | --- |
|  | **Beta (se)** | ***P* value** | **Beta (se)** | ***P* value** | **Intercept*** | ***P* value** |
| Evening chronotype |  |  |  |  |  |  |
| Pancancer incidence | -0.221 (0.202) | 0.273 | -0.200 (0.283) | 0.497 | -0.161 (0.377) | 0.679 |
| All-cause mortality of cancer patients | 0.047 (0.626) | 0.940 | 0.019 (0.778) | 0.981 | -0.125 (0.286) | 0.674 |
| 5-year Cancer mortality | -0.412 (1.379) | 0.772 | 0.045 (1.286) | 0.972 | 0.104 (0.167) | 0.550 |
| Cancer mortality | -0.051 (0.752) | 0.946 | -0.328 (0.839) | 0.705 | 0.098 (0.315) | 0.763 |

***** MR-Egger intercept was used to quantify the effect of directional pleiotropy. Values that significantly differ from zero indicated potential pleiotropy, which suggested exposure-associated genetic variables may influence the outcome through other pathways rather than through exposure.

**Table S51. Sensitivity analysis for short/long sleep duration and pancancer incidence and mortality of cancer patients using the Weighted median and MR-Egger methods.**

| **Outcomes** | **Weighted median** | | **MR-Egger** | | | |
| --- | --- | --- | --- | --- | --- | --- |
|  | **Beta (se)** | ***P* value** | **Beta (se)** | ***P* value** | **Intercept*** | ***P* value** |
| Short sleep duration |  |  |  |  |  |  |
| Pancancer incidence | -0.019 (0.234) | 0.934 | -0.101 (0.250) | 0.691 | 0.075 (0.236) | 0.752 |
| All-cause mortality of cancer patients | 0.115 (0.402) | 0.775 | 0.083 (0.469) | 0.861 | -0.001 (0.153) | 0.995 |
| 5-year Cancer mortality | 0.321 (0.673) | 0.632 | -0.096 (0.849) | 0.911 | 0.064 (0.182) | 0.728 |
| Cancer mortality | 0.119 (0.402) | 0.768 | -0.047 (0.492) | 0.925 | 0.091 (0.131) | 0.494 |
| Long sleep duration |  |  |  |  |  |  |
| Pancancer incidence | -0.037 (0.204) | 0.857 | 0.059 (0.445) | 0.899 | -0.182 (0.482) | 0.719 |
| All-cause mortality of cancer patients | 0.066 (0.606) | 0.913 | -0.106 (0.890) | 0.909 | 0.016 (0.164) | 0.926 |
| 5-year Cancer mortality | 0.205 (1.744) | 0.906 | 1.386 (2.300) | 0.569 | -0.059 (0.259) | 0.828 |
| Cancer mortality | -0.026 (0.648) | 0.968 | -0.311 (0.951) | 0.755 | -0.083 (0.246) | 0.748 |

***** MR-Egger intercept was used to quantify the effect of directional pleiotropy. Values that significantly differ from zero indicated potential pleiotropy, which suggested exposure-associated genetic variables may influence the outcome through other pathways rather than through exposure.

**Table S52. Sensitivity analysis for insomnia and pancancer incidence and mortality of cancer patients using the Weighted median and MR-Egger methods.**

| **Outcomes** | **Weighted median** | | **MR-Egger** | | | |
| --- | --- | --- | --- | --- | --- | --- |
|  | **Beta (se)** | ***P* value** | **Beta (se)** | ***P* value** | **Intercept*** | ***P* value** |
| Insomnia symptom |  |  |  |  |  |  |
| Pancancer incidence | 0.345 (0.164) | 0.036 | 0.749 (0.270) | 0.008 | -0.007 (0.002) | <0.001 |
| All-cause mortality of cancer patients | 0.121 (0.312) | 0.776 | 0.038 (0.569) | 0.947 | 0.022 (0.118) | 0.856 |
| 5-year Cancer mortality | -0.100 (0.306) | 0.839 | 0.316 (0.982) | 0.749 | -0.187 (0.089) | 0.042 |
| Cancer mortality | 0.129 (0.463) | 0.781 | 0.074 (0.621) | 0.905 | 0.004 (0.114) | 0.971 |

***** MR-Egger intercept was used to quantify the effect of directional pleiotropy. Values that significantly differ from zero indicated potential pleiotropy, which suggested exposure-associated genetic variables may influence the outcome through other pathways rather than through exposure.
